# Genome‐Wide Population Structure in a Marine Keystone Species, the European Flat Oyster (*Ostrea edulis*)

**DOI:** 10.1111/mec.17573

**Published:** 2024-11-12

**Authors:** Homère J. Alves Monteiro, Dorte Bekkevold, George Pacheco, Stein Mortensen, Runyang Nicolas Lou, Nina O. Therkildsen, Arnaud Tanguy, Chloé Robert, Pierre De Wit, Dorte Meldrup, Ane T. Laugen, Philine S. E. zu Ermgassen, Åsa Strand, Camille Saurel, Jakob Hemmer‐Hansen

**Affiliations:** ^1^ National Institute of Aquatic Resources Technical University of Denmark Silkeborg Denmark; ^2^ Section for Evolutionary Genomics, Faculty of Health and Medical Sciences The Globe Institute, University of Copenhagen Copenhagen K Denmark; ^3^ Department of Biosciences, Centre for Ecological and Evolutionary Synthesis University of Oslo Oslo Norway; ^4^ Institute of Marine Research Bergen Norway; ^5^ Department of Natural Resources and the Environment Cornell University Ithaca New York USA; ^6^ Department of Integrative Biology University of California Berkeley Berkeley California USA; ^7^ Sorbonne Université CNRS, UMR 7144, Station Biologique de Roscoff Roscoff France; ^8^ Department of Marine Sciences, Tjärnö Marine Laboratory University of Gothenburg Strömstad Sweden; ^9^ Department of Biological and Environmental Sciences University of Gothenburg Gothenburg Sweden; ^10^ Department of Ecology Swedish University of Agricultural Sciences Uppsala Sweden; ^11^ Department of Natural Sciences Centre for Coastal Research, University of Agder Kristiansand Norway; ^12^ Changing Oceans Group School of Geosciences, University of Edinburgh Edinburgh UK; ^13^ Department of Environmental Intelligence IVL Swedish Environmental Research Institute Fiskebäckskil Sweden; ^14^ National Institute of Aquatic Resources Danish Shellfish Centre, Technical University of Denmark Nykøbing Mors Denmark

**Keywords:** low coverage genome resequencing, natural resource management, nature restoration, *Ostrea edulis*, oyster, population genomics, structural variants

## Abstract

*Ostrea edulis,* the European flat oyster, was once a widespread economically and ecologically important marine species, but has suffered dramatic declines over the past two centuries. Consequently, there has been a surge in European restoration efforts, many of which focus on restocking as a conservation measure. In this study, we used whole‐genome sequencing (WGS) data to investigate the population structure, demographic history, and patterns of local adaptation of *O. edulis* across its natural distribution with increased sampling densities at Scandinavian localities. Results revealed seven distinct genetic clusters, including previously undescribed complex population structure in Norway, and evidence for introgression between genetic clusters in Scandinavia. We detected large structural variants (SVs) on three pseudo‐chromosomes. These megabase long regions were characterised by strong linkage disequilibrium and clear geographical differentiation, suggestive of chromosomal inversions potentially associated with local adaptation. The results indicated that genomic traces of past translocations of non‐native *O. edulis* were still present in some individuals, but overall, we found limited evidence of major impacts of translocations on the scale of contemporary population structure. Our findings highlight the importance of considering population structure and signatures of selection in the design of effective conservation strategies to preserve and restore wild native European flat oyster populations, and we provide direct knowledge safeguarding sustainable mitigation actions in this important species.

## 1 Introduction

Ecosystem restoration is recognised globally as important for achieving sustainability, specifically to address SDG14 (UN 2015) including the implementation of internationally binding targets (e.g., under the EU nature restoration law; European Union 2024). It has been highlighted that restoration efforts should account for the genetic diversity among and within populations, to secure ecosystem resilience to human‐induced pressures on natural systems, including climate change (Balvanera et al. 2022; Coleman et al. 2020). Adaptive genetic variation also plays a crucial role for natural population persistence and evidence of small‐scale patterns of genetic differentiation mediated by marine reproductive barriers is now recognised as more common than previously believed (Miller et al. 2020; Selkoe, Henzler, and Gaines 2008). Understanding the interaction of effects from local selection and gene flow is perhaps all the more important in marine species, because they are often characterised by high levels of dispersal and gene flow, which can both counteract the effects of local environmental selection (Nielsen et al. 2009) and facilitate dispersal of locally adapted genetic variation (Bontrager and Angert 2019). Consequently, knowledge of population genetic structure, local adaptation and gene flow and connectivity can provide valuable information for management strategies, e.g. through prioritisation of conservation efforts and to inform the spatial scales at which management, restoration and conservation actions are likely to have an impact, particularly in response to climate change (Xuereb et al. 2021) and habitat fragmentation (see Zarri et al. 2022, for an example in anadromous fishes). Yet, intraspecific genetic diversity is rarely considered under current natural resource management frameworks (Reiss et al. 2009; Norderhaug et al. 2024).

Recent advances in molecular biology techniques facilitate full genomic scale studies and offer numerous advantages for management and conservation, far surpassing what is possible with more reduced‐representation genetic/genomic data (see the reviews from Bernatchez et al. 2017; Supple and Shapiro 2018). For example, full genomic scale studies can increase statistical power for the detection of population structure and the examination of adaptive and neutral processes (Benestan et al. 2016; Clucas et al. 2019; Xuereb et al. 2021), provide improved demographic inferences (Atmore et al. 2022; North, McGaughran, and Jiggins 2021) and yield better predictions of population vulnerability to climate change (Fuller et al. 2020; Rellstab, Dauphin, and Exposito‐Alonso 2021). High‐throughput genomic methods can further reveal signatures of selection and adaptive divergence in localised parts of the genome (Lowry et al. 2017; Tiffin and Ross‐Ibarra 2014). Such patterns are widespread across the tree of life and can underpin local adaptation in (marine) invertebrates (Le Moan et al. 2023; Mérot et al. 2021) and fishes (Han et al. 2020; Therkildsen et al. 2019). Importantly, full genomic scale studies are now also possible in non‐model marine species (Devlin‐Durante and Baums 2017; Wood et al. 2020), facilitating in‐depth studies on a large range of taxa of conservation concern. Consequently, genomic data can make a practical contribution to nature restoration (Gruenthal et al. 2014; Wood et al. 2020) of relevance for reaching global sustainability goals.

The native European flat oyster, *Ostrea edulis*, serves as a valuable model for demonstrating the added value of genomic data in management and conservation. This keystone species was once widespread along the European coasts and has been subject to historical fishing and cultivation practices (Astrup et al. 2019; Matthiessen 2001; Thurstan et al. 2024). Intense fishing pressure, in combination with other factors such as disease outbreaks, have resulted in local extirpations and an increasingly patchy distribution across the former range of the species (Thurstan et al. 2024; Zu Ermgassen et al. 2023). Although historical records document European flat oyster reefs extending for hectares, current occurrences rarely extend beyond a few m^2^ (zu Ermgassen et al. 2023). As a result of the dramatic decline in population distribution and population size, the European flat oyster ecosystem has recently been classified as “collapsed” under the IUCN Red list of Ecosystems Framework (Zu Ermgassen et al. 2023). Because of population declines, remnant wild populations have likely experienced a reduction of connectivity and genetic diversity, potentially leading to reduced adaptive potential to environmental perturbations (Kardos et al. 2021). Against this backdrop, the species has been subject to intense translocations to support local production (Bromley et al. 2016). Although historical records of translocations are incomplete, it is clear that millions of individuals have been translocated between several countries on a European scale (Bromley et al. 2016). A recent study found clear genetic signatures of translocations from the Atlantic in the contemporary populations in the Mediterranean Sea (Lapègue et al. 2022), suggesting that past translocations can leave permanent genetic signatures in recipient populations. Thus, the practice can obscure natural population structure and lead to misconceptions about adaptive drivers (Simon et al. 2020; Waples, Punt, and Cope 2008). Currently, restoration projects are undertaken in several parts of the species' range to counter local declines, involving aquaculture‐based seed production (Colsoul et al. 2021; Rodriguez‐Perez et al. 2020) and occasionally sourcing non‐native populations (Sas et al. 2020; zu Ermgassen et al. 2020a) that may be genetically maladapted to the recipient environments. A better resolved map of the genomic diversity in the remaining wild populations has been identified as a critical need to support management actions with biological conservation objectives, such as the (re)introduction of oysters for restoration efforts (zu Ermgassen et al. 2020b).

Genetic studies on *O. edulis* diversity, employing limited numbers of markers (Diaz‐Almela et al. 2004; Johannesson, Rödström, and Aase 1989; Lallias et al. 2010; Lapègue et al. 2022; Saavedra et al. 1993; Saavedra, Zapata, and Alvarez 1995; Vera et al. 2016), or thousands of single‐nucleotide polymorphisms (SNPs; Sambade et al. 2022; Vera et al. 2019) and the full mitochondrial genome (Hayer et al. 2021), demonstrate the presence of four geographically distinct genetic clusters: North Sea, Atlantic Ocean, Western Mediterranean Sea and Eastern Mediterranean Sea extending to the Black Sea (Lapègue et al. 2022; Vera et al. 2019). In addition, earlier studies have included single sampling localities from Scandinavia, and have often found these to be divergent from other sampling sites (Launey 2002; Sobolewska and Beaumont 2005; Beaumont et al. 2006; Vera et al. 2019). Recently, Robert et al. (2024; manuscript in preparation) used genome resequencing and a dense sampling grid with a particular focus on the Swedish and Norwegian coasts, and found evidence of population structure on local geographical scales in the species. With the additional information provided by four recent reference genome assemblies (Adkins and Mrowicki 2023; Boutet et al. 2022; Gundappa et al. 2022; Li et al. 2022), it has become apparent that a structural variant located at the end of pseudo‐chromosome 8 may play a role in resilience against the widespread lethal disease bonamiosis (Sambade et al. 2022) caused by *Bonamia ostreae*, which affects both wild and cultured populations (Holbrook et al. 2021; Mortensen and Skår 2019). Nonetheless, studies with full genome coverage across sampling sites spanning the species' distribution are still lacking.

To address the knowledge gap regarding the genetic diversity of *O. edulis*, we adopted a whole‐genome sequencing approach, leveraging millions of genome‐wide markers to investigate the population structure and connectivity of *O. edulis* across its current distribution range. We aimed to provide novel insights into the evolution of natural populations and to generate a robust baseline for conservation management and restoration programs. The sampling regime reflected the current distribution range of the remaining wild populations of the species and a particular focus on previously under‐explored Scandinavian (northern species range) localities. By incorporating a comprehensive genomic approach, we provide insights and recommendations for the conservation, management and restoration of this keystone marine species, which is heavily exposed to anthropogenic pressures.

## 2 Materials and Methods

### 2.1 Sampling

**FIGURE 1:**
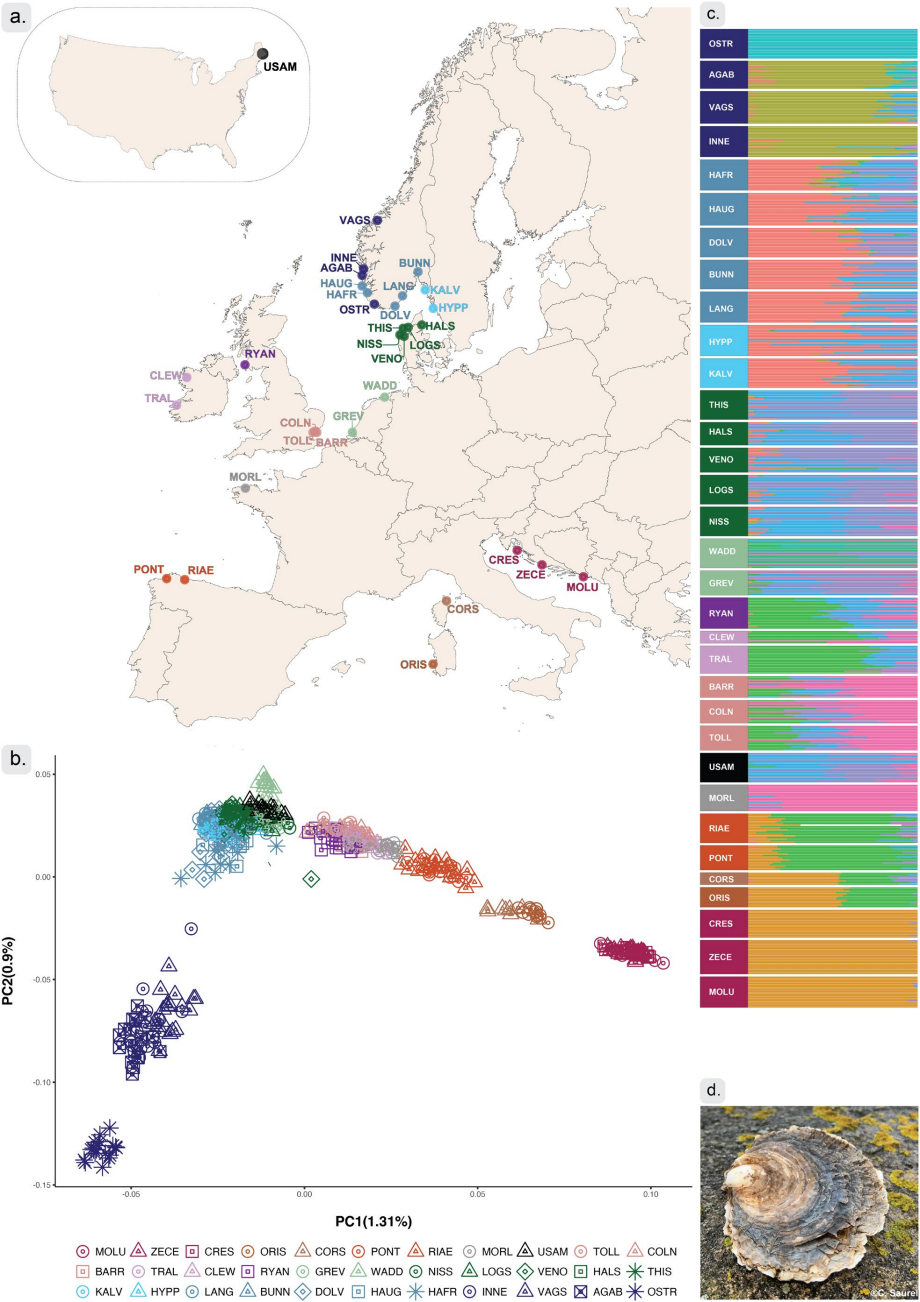
Sampling sites and genetic clusters. (a) Sampling sites for *O. edulis*; colours match the clustering in the PCA. (b) PCA plot of the first two principal components using 1,404,180 LD‐pruned genome‐wide SNPs. (c) Individual admixture proportions by sampling site, using LD‐pruned genome‐wide SNPs, for ten ancestral (K) clusters (NB. colours differ between (b) and the admixture proportions in (c). (d) *Ostrea edulis* adult, Limfjorden, Denmark (2024) (Photo credit: Camille Saurel).

We collected 582 samples of wild oysters representing 33 sampling sites in Europe and in North America (Table 1, Figure 1a). Gill and mantle tissues were sampled and preserved in 96% ethanol. Genomic DNA was extracted using the DNeasy Blood Tissue kit (Qiagen) for all sample sites except for USAM and MORL, where sequence data were already available prior to our study. DNA concentration was measured using the Broad Range protocol of the Qubit version 2.0 and standardised to 20 ng/μL. DNA extracts included samples from ten sites (CLEW, CORS, CRES, GREV, MOLU, PONT, RYAN, TRAL, WADD and ZECE) included in previous genetic studies (Table 1) and resequenced for this study.

**TABLE 1:** Sampling sites.

Geographical origin	Water body	Sampling site	Year	No. Low coverage	No. MtDNA	No. High coverage	Longitude (decimal degrees)	Latitude (decimal degrees)	Status	Tag	Included in previous genetic study	References
Croatia	Adriatic Sea	Molunat	2018	20	20		18.21	42.54	Wild	MOLU	Yes	https://doi.org/10.3389/fmars.2020.00084
		Zečevo	2017	22			15.87	43.67	Wild	ZECE	Yes	https://doi.org/10.3389/fmars.2020.00084
		Cres	2018	18	17		14.52	44.68	Wild	CRES	Yes	https://doi.org/10.3389/fmars.2020.00084
Italy (Sardinia)	Mediterranean Sea	Golfo di Oristano	2021	13	14	1	8.29	39.54	Wild	ORIS	No	—
France (Corsica)		Étang de Diane	Unknown	8	7		9.54	42.71	Cultured	CORS	No	—
Spain	Atlantic Ocean	Pontedeume	2013	16	15	2	−8.18	43.41	Wild	PONT	Yes	https://doi.org/10.1111/eva.12832
		Ría Eo	2013	19	18		−7.04	43.52	Wild	RIAE	Yes	https://doi.org/10.1111/eva.12832
Ireland		Tralee Bay	2013	18	17		−9.93	52.29	Wild	TRAL	Yes	https://doi.org/10.1111/eva.12832
		Clew Bay	2013	8	5	1	−9.61	53.8	Wild	CLEW	Yes	https://doi.org/10.1007/s00227‐016‐3012‐x
U.S.A		State of Maine	Unknown	19			Unknown	Unknown	Cultured	USAM	No	—
France (Brittany)	English Channel	Morlaix	2020	17	14		−3.85	48.63	Wild	MORL	No	—
UK (England)		Tollesbury	2020	16	14		0.87	51.75	Wild/Cultured	TOLL	No	—
		Barrow Deep	2020	14	12		1.16	51.75	Wild/Cultured	BARR	No	—
		River Colne	2020	15	12		1	51.81	Wild/Cultured	COLN	No	—
UK (Scotland)	Irish sea/Loch Ryan	Lochryan	2020	20	16		−5.03	54.95	Wild	RYAN	Yes	https://doi.org/10.1111/eva.12832
The Netherlands	North Sea	Lake Grevelingen	2020	16	16		3.91	51.76	Wild/Cultured	GREV	Yes	https://doi.org/10.1111/eva.13446
		Wadden Sea	2020	19		1	6.59	53.49	Wild	WADD	Yes	https://doi.org/10.1111/eva.13446
Denmark	Limfjorden	Venø	2020	16			8.69	56.53	Wild	VENO	No	—
		Nissum	2020	19	17		8.33	56.63	Wild	NISS	No	—
		Thisted	2020	19	15		8.69	56.95	Wild	THIS	No	—
		Løgstør	2020	19	17		9.14	56.97	Wild	LOGS	No	—
	Kattegat	Hals	2021	15	12		10.37	57	Wild	HALS	No	—
Sweden	Kattegat	Hyppeln	2020	20	16	1	11.61	57.76	Wild/Cultured	HYPP	No	—
	Skagerrak	Kalvö	2020	19	15		11.15	58.77	Wild	KALV	No	—
Norway		Bunnefjorden	2021	19	17		10.72	59.74	Wild	BUNN	No	—
		Langessand	2021	20	15		8.95	58.62	Wild	LANG	No	—
		Dolsvågskilen	2021	19	19		8.15	58.13	Wild	DOLV	No	—
	North Sea	Ostretjønn	2020	19	19		6.25	58.32	Wild/Cultured	OSTR	No	—
		Hafrsfjord	2020	20	15		5.66	58.93	Wild	HAFR	No	—
		Haugevågen	2020	21	21		5.19	59.29	Wild	HAUG	No	—
		Aga Bømlo	2020	18	14		5.24	59.84	Wild/Cultured	AGAB	No	—
		Innerøyen lagoon	2020	20	19		5.41	60.17	Wild/Cultured	INNE	No	—
	Norwegian Sea	Vågstranda	2020	21		1	7.17	62.62	Wild/Cultured	VAGS	No	—

*Note:* Summary of sampling sites with geographical coordinates, including number of low coverage and high coverage sequenced individuals, individuals used for the mitochondrial DNA analysis, population status (wild, cultured, or unknown status), sampling site code (“Tag”), and reference to previous genetic studies, where appropriate. The status “Wild/Cultured” denotes a natural population but with historical or current breeding practices (often of unknown scale and quantity) recorded in the vicinity of the sampling site.

### 2.2 Sequencing

We sequenced 546 individuals sampled from 31 sites using low‐coverage whole‐genome sequencing (lcWGS) at a target of ~1.5× per individual. The 19 “USAM” (Maine, USA) and the 17 “MORL” (Morlaix, France) individuals were sequenced at 15× depth. A further subset of 7 individuals from six sites were also resequenced at higher coverage (~18× per individual) for a demographic history analysis (see below).

lcWGS sequencing library batches of 96 individuals were randomised with respect to sample sites to limit batch effects (Lou and Therkildsen 2022), and contained two technical replicates each. We prepared a separate, dual‐indexed library for each individual with a highly cost‐effective protocol, as described by Therkildsen and Palumbi (2017). The libraries were sequenced with paired‐end 150‐bp reads on an Illumina NovaSeq 6000 platform.

### 2.3 Sequence Filtering and Alignment

We used FastQC v0.11.8 (Andrews 2010) to check read quality and Trimmomatic v0.38 (Bolger, Lohse, and Usadel 2014) to remove adapters (see Supporting Information and Methods for additional details). The trimmed reads were mapped against the indexed *Roscoff_O.edulis‐V1* genome (Boutet et al. 2022) using the *bwa‐mem* algorithm of bwa v.0.7.17 (Li 2013) with default settings. We removed duplicate reads using the Picard v2.25.2 MarkDuplicates tool (http://broadinstitute.github.io/picard/) and clipped redundant sequences from the overlapping ends of each mapped read pair using the bamutil v1.0.14 clipOverlap tool (Jun et al. 2015) with default settings. Finally, reads were realigned around indels using the GATK v.3.8 IndelRealigner tool (McKenna et al. 2010), creating target intervals across all individuals, and using the default settings for realignment. We calculated sequencing depth statistics using the Samtools v.1.14 (Li et al. 2009) depth tool. We performed the same data processing steps for the seven individuals sequenced at > 18×. Since USAM and MORL were sequenced with a 15× depth target, we subsequently down sampled sequences for these individuals directly within raw fastq files, to 1.5–2× using bbmap 38.90 (Bushnell 2014) with the option *samplerate = 0.2* to ensure the congruity with sequencing data from the other 31 sampling sites.

### 2.4 Genotype Likelihood Estimation

Prior to genotype likelihood estimation, we used ANGSD v0.940 (Korneliussen, Albrechtsen, and Nielsen 2014) with *‐doDepth* 1 to obtain a read depth distribution (Figure S1) to set up the ANGSD filters for genotype likelihood estimation. Subsequently, we used ANGSD to estimate genotype likelihoods using the Samtools model (*−GL* 1) for the low‐coverage dataset. To identify polymorphic loci we established reasonable depth filters given the average depth per individual in the raw data (*‐setMinDepthInd* 1, *‐setMinDepth* 600, *‐setMaxDepth* 1200) on top of the filters based on aspects of read‐quality (*‐uniqueOnly* 1, *−remove_bads* 1, *−trim* 0, *‐C* 50 and *‐baq* 1), base quality and mapping quality (*−minQ* 20, *−minMapQ* 20) for subsequent analysis based on the genotype likelihood estimation. SNPs were identified across all individuals. We established the *p*‐value cutoff of 10^−6^ under the likelihood ratio (*‐SNP_pval* 1e‐6) on top of the above‐described depth and quality filters to only retain SNPs. Major and minor alleles were inferred from genotype likelihoods across all 582 individuals (*−doMaf* 1 *‐doMajorMinor* 1) and sites were retained if the minor global allele frequency was above 5% (*−minMaf* 0.05). Sites with more than two inferred alleles were purged using *‐rmTriallelic* 0.05 flag. SNPs retrieved from this step are compiled in Dataset I (see Table S1).

### 2.5 Linkage Disequilibrium Pruning

Linkage disequilibrium (LD) pruning was applied to remove correlated variants that may cause bias when estimating population structure. LD was calculated using ngsLD v1.1.1 (Fox et al. 2019) on Dataset I. We ran ngsLD with *‐‐max_kb_dist 100* to detect SNPs in LD within 100 kb distance, and subsequently performed pruning using the *prune_ngsLD.py* script from the same program, after LD‐decay visualisation by plotting LD estimates against physical distance (Figure S2; see Supporting Information Materials and Methods for additional details). The generated dataset is Dataset II (see Table S1).

### 2.6 Population Structure and Population Admixture

We generated two files with ANGSD, using Dataset II as input with the option *–sites*, an allele frequencies file (using *‐doMaf* 1) and a genotype likelihoods file (using *‐GL* 1 and *‐doGlf* 2), using samples that passed both sample and site filtering. We performed an individual‐level principal component analysis (PCA) using the covariance matrix calculated with the option *‐doCov* 1. To examine the potential impact of missing data on our lcWGS approach, we calculated per‐individual missing data in the SNP panel and examined whether they were significantly associated with genetic differentiation in the PCA (Figure S3). The analyses showed a significant relationship between individual location on the first PC axis, but that missingness only explained 6% of the variation along PC1 (Figure S3). There was no association between missingness and individual PC2 coordinates. These results indicate limited effects of missingness on downstream analyses. Likewise, sequencing batch did not show a clear relationship with individual clustering in the PCA (Figure S3). We then estimated admixture proportions for individuals using NGSadmix (Skotte, Korneliussen, and Albrechtsen 2013) on genotype likelihoods of Dataset II, using 2000 iterations from *K = 2* to *K = 10* until convergence, which we defined as a maximum difference of two log‐likelihood units between the top three maximum likelihood results. For each converged run of *K*, we subsequently evaluated the model fit using evalAdmix v.0.962 (Garcia‐Erill and Albrechtsen 2020).

Our low‐coverage (of the nuclear genome) sequencing allowed us to recover a high‐confidence full mitochondrial genome sequence for most of the individuals (Lou et al. 2018; Therkildsen and Palumbi 2017). We mapped reads from a subset of 428 individuals, representing all major genetic cluster in the data, to the Danish‐Limfjord mitochondrial reference genome (GenBank acc. no. *MT663266*; Hayer et al. 2021), and used ANGSD (*‐doCounts* 1, *‐dumpCounts* 4) for each individual to count observed alleles at each position of the mitochondrial genome. Then, we converted allele count data output from ANGSD into a data frame containing the consensus sequences for each of the 428 individuals, with the major allele chosen to be the consensus allele for each position if the sequencing depth was ≥ 4× and if the major allele frequency was ≥ 0.75 (otherwise the position was given an N). A Tight Span Walker haplotype network was produced from these consensus sequences using PopART (Leigh and Bryant 2015).

### 2.7 Population Genetic Summary Statistics, Relatedness and Demographic History

To estimate several key population genetic summary statistics, the fraction of heterozygous sites (*H*_*o*_), nucleotide diversity (*π*), Watterson's *θ* (*θ*_*w*_) and Tajima's D per sampling site, we calculated the Site Frequency Spectrum (SFS) for the 33 sampling sites with ANGSD v0.940 (Korneliussen, Albrechtsen, and Nielsen 2014; Dataset III; see Supporting Information Materials and Methods for additional details). The observed fraction of heterozygous sites (*H*_*o*_) was calculated as the ratio of the number of heterozygotes to the total number of sites with information, expressed as a percentage. Nucleotide diversity (*π*) and Watterson's *θ* (*θ*_*w*_) are both measures of the overall level of genetic variation within a population. Tajima's D allows us to evaluate deviations from neutral evolution, with a positive genome‐wide Tajima's D indicative of a sudden population contraction. To calculate heterozygosity and express it as a percentage for all individuals, we re‐ran ANGSD *‐doMaf* followed by *‐doSaf* to estimate allele frequencies at all variant and invariant sites using the genotype likelihoods. This generated Dataset IV, a minimum allele frequency and Site Frequency Spectrum for all the individuals at all sites (see Table S1).

We assessed the relatedness within each of the 33 sampling sites using NgsRelate (Korneliussen and Moltke 2015), with allele frequency and genotype likelihoods estimated for each population with ANGSD. This process resulted in Dataset V (allele frequencies per individual for each sampling site for relatedness calculation, see Table S1 and Supporting Information Materials and Methods for additional details), which included allele frequencies per individual for the relatedness calculation. The frequency column from the allele frequency file was then extracted and used in conjunction with the genotype likelihood file by NgsRelate (Korneliussen and Moltke 2015). We employed the Hedrick and Lacy method for estimating pairwise relatedness between pairs of individuals within the same population (Hedrick and Lacy 2015).

We reconstructed the demographic history of the seven *O. edulis* individuals included in Dataset VII (with minimum sequence coverage above 18×) to investigate the dynamics of effective population size (*N*_*e*_) over the past several thousand years using PSMC (Li and Durbin 2011). To estimate historical *N*_*e*_, we used the following parameters: *‐N25 ‐t15 ‐r5 ‐p ‘4 + 25* * *2 + 4 + 6’*. To plot results, we set a generation time (*g*) to 1 year for all individuals, keeping in mind that sexual maturity varies across latitude (Colsoul et al. 2021). Mutation rate (*μ*) was fixed to the value previously reported for estuarine oysters (*Crassostrea ariakensis*): 0.3 × 10^−8^ per basepair per year (Li et al. 2021).

### 2.8 F_ST_ ‐Based Analysis (With Dataset I as Primary Input)

Weighted pairwise F_ST_ was estimated between sampling sites using ANGSD to determine the degree of genetic differentiation among geographic regions and between sampling sites, using the 2D site frequency spectrum for each pair of sampling sites (*realSFS*) and calculated as the average pairwise weighted F_ST_ (*realSFS fst*; see Supporting Information Materials and Methods for additional details). This generated Dataset VI (F_ST_ for each pair of sampling sites). Next, to evaluate patterns of genomic differentiation, we created Manhattan plots of pairwise F_ST_ in nonoverlapping 15 kb windows using ANGSD (*realSFS fst stats2*). A Mantel test was performed to test for isolation by distance, which is the expected relationship when genetic differentiation increases with geographic distance. The Mantel test was performed with the *mantel.randtest()* function using of the *adegenet* R package (Jombart 2008). The pairwise geographic distances were measured in kilometres and calculated using sampling site GPS coordinates. The genetic distances (GD) for isolation by distance analyses were calculated using genome‐wide average F_ST_ estimates as GD=FST1−FST, following Rousset (1997).

### 2.9 Genome Scans and Identification of Large Structural Variants (With Dataset I as Primary Input)

We used PCAngsd (Meisner, Albrechtsen, and Hanghøj 2021) with the *‐‐selection* flag in a population‐blind approach, to identify SNPs that show significantly different covariance structure and therefore could be under selection (“selection scan”). To discern potential structural chromosomal rearrangements and their geographical occurrences, we performed a local PCA analysis employing the R package *lostruct*, which detects genomic regions of abnormal population structure that may be caused by structural variants (Li and Ralph 2019; following scripts outlined in https://github.com/therkildsen‐lab/genomic‐data‐analysis/blob/master/lcwgs_data_analysis.md#localpca; see Supporting Information Materials and Methods for additional details).

### 2.10 Genotype Frequencies of Large Structural Variants (With Dataset I as Primary Input)

To explore the spatial distribution of three large genomic regions suggested to harbour structural variants (SVs, see Results), we characterised their sampling site‐specific genotype frequencies. A segregating polymorphic SV will display a typical PCA pattern with two homozygote groups for the SV and an intermediate, heterozygous group. We first utilised the “Local PCA” analysis results to estimate the breakpoints of three large structural variants. Then, to explore the LD patterns in the pseudo‐chromosomes with putative SVs, we ran ngsLD for the SNPs at pseudo‐chromosomes 4, 5 and 8, with *‐max_kb_dist* disabled and visualised results using the *LDheatmaps* package in R. Subsequently, we performed PCAs using a covariance matrix for each set of SNPs inside each delimited SV region for all 33 sampling sites. We used clustering patterns in the PCA to define individual genotypes for the SVs, then calculated the genotype frequencies for each sampling site and SV and tested for deviations from Hardy–Weinberg equilibrium (HWE) using exact tests implemented in the *genepop* R package (Rousset, Lopez, and Belkhir 2020). We also used the allele frequencies of the SV alleles to calculate pairwise F_ST_ though the formula F_ST_ = (H_T_‐H_S_)/H_T_, following Nei (1973). The pairwise F_ST_ matrices for the SVs were used in Mantel tests to test correlations between genetic and geographic distance (isolation by distance) and between population structure observed with genome wide data and for the individual SVs.

To facilitate a direct comparison to results in previous studies identifying putative SVs in *O. edulis*, we used blastn from the BLAST+ application (Camacho et al. 2009) with default parameters to map SNP sequences from Lapègue et al. (2022) to the reference genome used in the present study.

Finally, to assess absolute genetic differentiation (d_xy_) and heterozygosity (see the above 2.7. *Population genetics estimates* section) among SV homozygote genotypes, d_xy_ was calculated on a per‐SNP basis (inside and outside SVs) with the *calcDxy.R* script (https://github.com/mfumagalli/ngsPopGen/blob/master/scripts/calcDxy.R). Our hypothesis posited that individuals with the more diverse homozygous genotype for each structural variant (“α”, see results) would exhibit a higher d_xy_ and average heterozygosity, reflecting the ancestral allelic state.

## 3 Results

### 3.1 Sequencing Results

After removing 14 individuals with genome coverage of < 30%, 582 individuals were retained in analyses. The mean sequencing coverage per individual was ~1.3×, with on average 60% of the genome covered per individual (see Figure S4). The mean sample size per sampling site was 17.6 (ranging from 8 to 22) individuals. For the variant identification using ANGSD, a total of 840,354,420 sites were analysed for the 582 individuals, with 5,684,643 SNPs retained after filtering (Table S1, Dataset I). After LD pruning, 1,404,180 sites were retained (Table S1, Dataset II). The high‐coverage dataset comprised seven genomes at a mean sequencing coverage per individual of ~31.4x and with 87% of the genome covered per individual (see Figure S5).

### 3.2 Population Structure, Relatedness, Mitochondrial Haplotype Network and Population Genetics Estimates

The PCA suggested seven geographically distinct clusters for the 33 sampling sites (Figure 1b). This included (1) an Adriatic Sea cluster with Croatian sampling sites; (2) a Mediterranean Sea cluster with French‐Corsican and Italian‐Sardinian sampling sites; (3) an Atlantic Ocean cluster with Spanish‐Galician sampling sites, (4) an English Channel, Ireland and British Isles cluster, including sampling sites from French Brittany, England, Ireland, and Scotland and (5) a Netherlands‐Wadden Sea/Scandinavian cluster encompassing samples from the Netherlands, Denmark, Sweden, South Norway, some of the Norwegian west‐coast and the North American site. Finally, there was (6) a distinct cluster composed of some of the sampling sites from the west coast of Norway and (7) a cluster only represented by the Norwegian sampling site Ostretjønn (OSTR; Figure 1b; Figure S6a). The PCA also showed divergence, roughly segregating sampling sites from Scandinavian and the Netherlands on PC1 (explaining 1.31% of the observed variation, Figure 1b). PC1 also indicated a clear geographical separation, following a transect from the East Mediterranean to Northern Europe (Figure 1b) The second principal component (PC2, which explained 0.9% of the observed variation) separated four Norwegian sites (INNE, VAGS, AGAB and OSTR) from the remaining Scandinavian sites (Figure 1b). It was noteworthy that some individuals from Scandinavia (from sampling sites DOLV and HAFR, and to a lesser extent from HAUG, INNE and VAGS) appeared to be intermediate between the Scandinavian genetic cluster and the divergent Norwegian clusters on PC2, a pattern which was confirmed in a geographically focused analysis including only Northern European sampling sites (Figure S7). The Wadden Sea sample (WADD), although part of the “Scandinavian” cluster on PC1, appeared to be separated into three distinct groups on other PCs (Figure 1b; Figure S6b). We note that patterns in Figure 1 appeared to be robust to the inclusion of some SNPs that may not have been filtered out during LD pruning considering the large size of the putative structural variants (see below) since patterns were reproduced with a dataset excluding SNPs in pseudo‐chromosome 4, 5 and 8 (Figure S6c). The relatively low proportion of variance explained by the PCA axes suggest that only part of the genomic variability is associated with population of origin. However, the pairwise F_ST_ estimates (ranging from 0.01 to 0.22, Figure S8a) support the individual based analyses in the PCA and suggest the existence of substantial population structure.

The isolation‐by‐distance analysis for the 32 sampling sites found a significant correlation (*r* ^2^ = 0.6415, *p* < 0.0001) between genetic and geographic distances (Figure S8b). The same analysis, excluding sampling sites in the Mediterranean, also showed a significant correlation between genetic and geographic distances (*r* ^2^ = 0.2895, *p* = 0.0245, Figure S8c).

Analyses of relatedness showed an absence of related individuals for most sampling sites, except for the sampling sites from the Wadden Sea (WADD and GREV) where some related individuals were observed (Figure S9).

The findings regarding population structure were further supported by results of the population admixture analysis. The model with ten ancestral source populations (K = 10) fit the data well without excessive correlation of residuals (Figure S10), although it should be noted that models with other numbers (including more than the maximum of 10 analysed in this study) of ancestral sources could potentially also be informative (Figure S10). Individual genotypes from most sampling sites aligned with admixture proportion expectations for their respective geographical regions. The single North American site exhibited an admixture profile similar to those found in Scandinavia and to one of the samples from the Netherlands (GREV).

The mitochondrial haplotype network revealed two major clades separated by 96 mutations. The first clade was “cosmopolitan” and included samples from the Mediterranean, Atlantic Ocean, North Sea and Scandinavia, whereas the other was largely restricted to Scandinavian sampling sites (Figure S11).

Genetic diversity estimates (heterozygosity, nucleotide diversity (π) and Watterson's *θ* (*θw*)) varied among sampling sites, with reduced estimates of diversity indicated for a few locations (OSTR, CLEW). Estimates of Tajima's *D* were positive for all sampling sites, with higher estimates observed for CLEW, WADD and OSTR (Figure S12). While diversity estimates were significantly related to sequencing depth, differences in sequencing depth did not seem to explain the extreme values for these sampling sites (Figure S12c).

### 3.3 Demographic History

**FIGURE 2:**
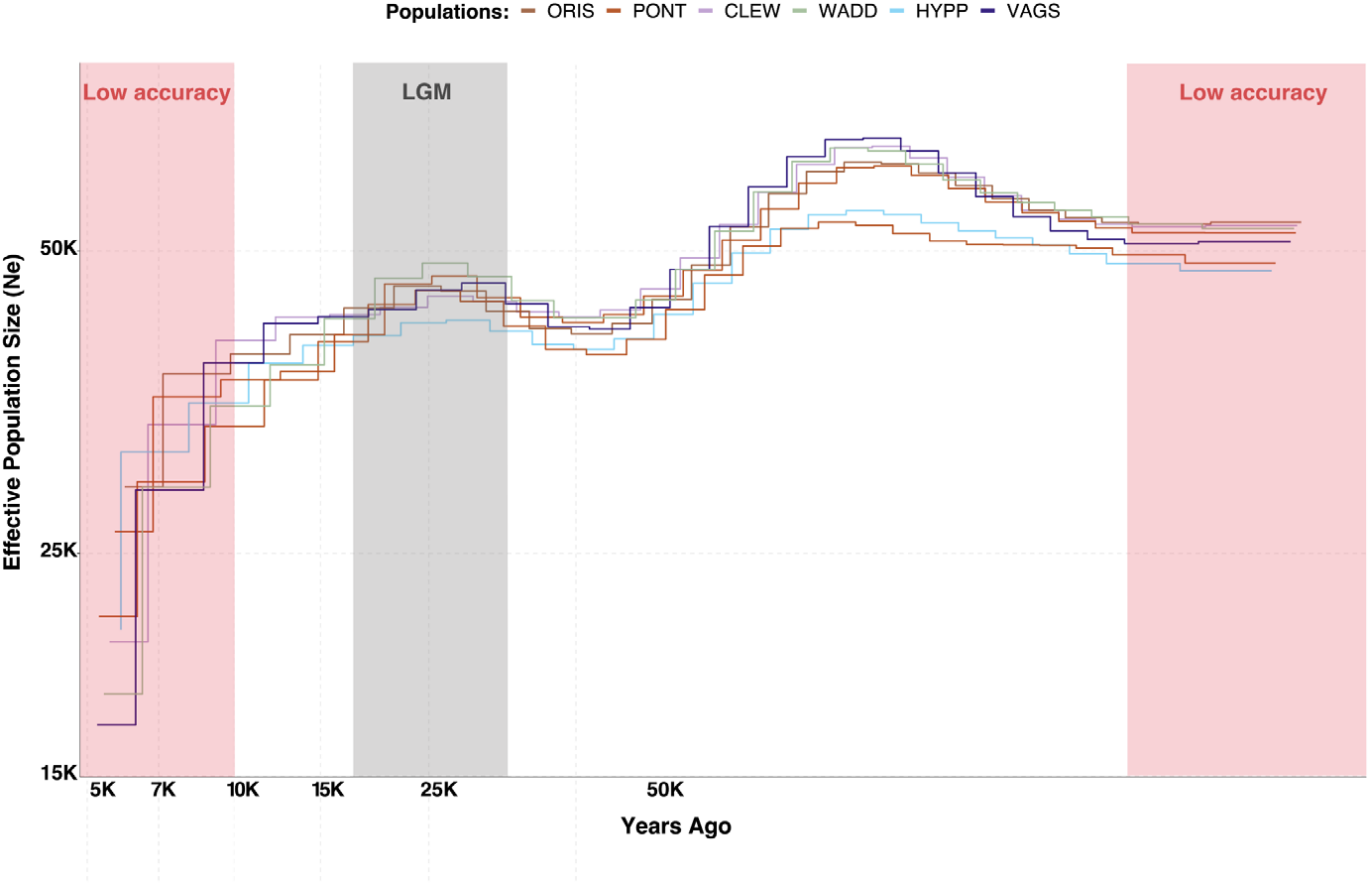
Demographic histories of *O. edulis* populations. The inferred demographic histories inferred from seven *O. edulis* individuals with the Last Glacial Maximum (LGM) period highlighted in grey. Areas shaded in red indicate periods where PSMC estimates of effective population size (Ne) are less reliable (Li and Durbin 2011; Mather, Traves, and Ho 2020; Nadachowska‐Brzyska et al. 2016): The recent past (up to 10,000 years ago) and the distant past (beyond 100,000 years ago).

Overall, all individuals showed similar demographic trends. The results indicated a slight decrease in the effective population size (*N*_*e*_) towards the end of the Würm glaciation period (WG), between 10,000 and 120,000 years ago (Figure 2). A slight recovery followed during the last glacial maximum (LGM), which occurred between 10,000 to 50,000 years ago, and a steep decrease after the LGM.

### 3.4 Genomic Regions With Elevated Genetic Differentiation and Linkage Disequilibrium

**FIGURE 3:**
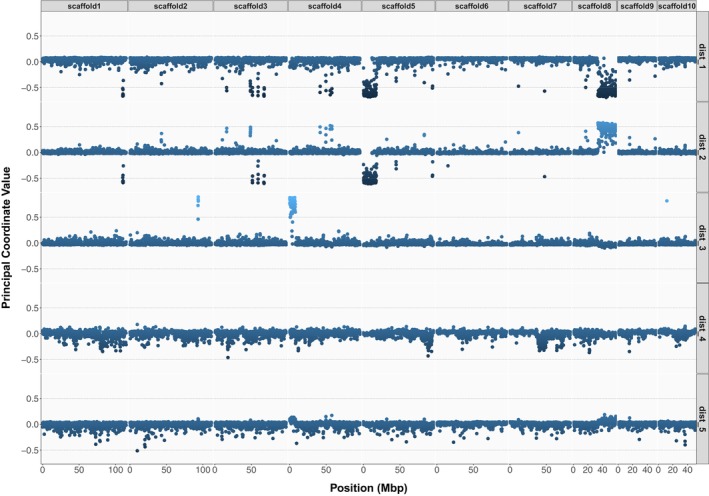
Local_PCA genome scan. Multidimensional scaling of local PCAs. For each MDS axis (1–5), the *y*‐axis represents the MDS value of each local PCA matrix based on windows of 1000 SNPs. The *x*‐axis is the position of the window along the 10 pseudo‐chromosomes. Each data point represents one SNP window's principal coordinate value on the corresponding axis, with the colour gradient indicating the magnitude of the value (extremely positive value in light blue, “baseline” in blue and extreme negative value in dark blue).

We identified several outlier regions along the first five MDS axes in the ‘local PCA’ analysis (Figure 3). In particular, large sections of highly clustered outlier windows were visible for pseudo‐chromosome 5 and 8 on MDS axes 1 and 2, and for pseudo‐chromosome 4 on MDS axis 3 (Figure 3). Notably, MDS has previously been employed to detect non‐recombining haplotypic blocks in various studies (Huang et al. 2020; Li and Ralph 2019; Mérot et al. 2021). In our case, these regions were also characterised by elevated LD (*r* ^2^ > 0.8) over large and continuous pseudo‐chromosome sections (Figure S13), suggesting the presence of structural variants (SVs), such as inversions, in the genome. These putative SVs formed large contiguous blocks of highly elevated differentiation, spanning almost half the length of a pseudo‐chromosome for the largest one (Figure 3; Figure S14). We label these variants as “Chr04:22Kb_sv”, “Chr05:172Kb_sv” and “Chr08:33800Kb_sv” to indicate their approximate genomic start coordinates. We found 459 annotated genes in “Chr04:22Kb_sv”, whereas “Chr05:172Kb_sv” and “Chr08:33800Kb_sv” contained respectively 937 and 1116 genes (Table 2), using the genome annotation described by Boutet et al. (2022).

**TABLE 2:** Structural variant characteristics.

SV ID	Pseudo‐chromosome (Boutet et al. 2022)	No. of genes	No. of SNPs	Length	Start genomic position	Stop genomic position
“Chr04:22Kb_sv”	scaffold4	459	85,239	7.8 Mbp	22,500	7,807,500
“Chr05:172Kb_sv”	scaffold5	937	171,821	11.2 Mbp	172,500	11,362,500
“Chr08:33800Kb_sv”	scaffold8	1116	191,366	24.5 Mbp	33,832,500	58,357,500

*Note:* Estimation of length, number of genes, number of SNPs and putative breakpoints of the megabase‐sized chromosomal SVs in *O. edulis* genomes.

The PCAngsd selection scan captured outlier loci from PC2 to PC10 (Figure S15). However, there was no overlap in outlier loci across PCs, indicating that the outlier loci detected might have occurred in different genomic regions in each population. Nevertheless, we re‐identified the three large regions, “Chr04:22Kb_sv”, “Chr05:172Kb_sv” and “Chr08:33800Kb_sv”, that behaved differently from the rest of the genome (Figure S15). Linked and highly divergent SNPs identified by Lapègue et al. (2022), were all, except for a few cases, mapped to genomic locations inside or in proximity to the SVs “Chr05:172Kb_sv” and “Chr08:33800Kb_sv” identified in the present study (Table S2). This suggests that the linkage blocks “LDG_182” and “LDG_202” identified by Lapègue et al. (2022) correspond to “Chr08:33800Kb_sv” and “Chr05:172Kb_sv”, respectively.

### 3.5 Genotype Frequencies of Putative SVs

To identify patterns of genetic variation consistent with SV polymorphisms, we characterised spatial population structure with PCAs of the SVs. In the SV located in pseudo‐chromosome 4, “Chr04:22Kb_sv”, there were 85,239 SNPs. The SV located in pseudo‐chromosome 5, “Chr05:172Kb_sv”, contained 171,821 SNPs and there were 191,366 SNPs in the SV “Chr08:33800Kb_sv” at pseudo‐chromosome 8 (Table 2).

**FIGURE 4:**
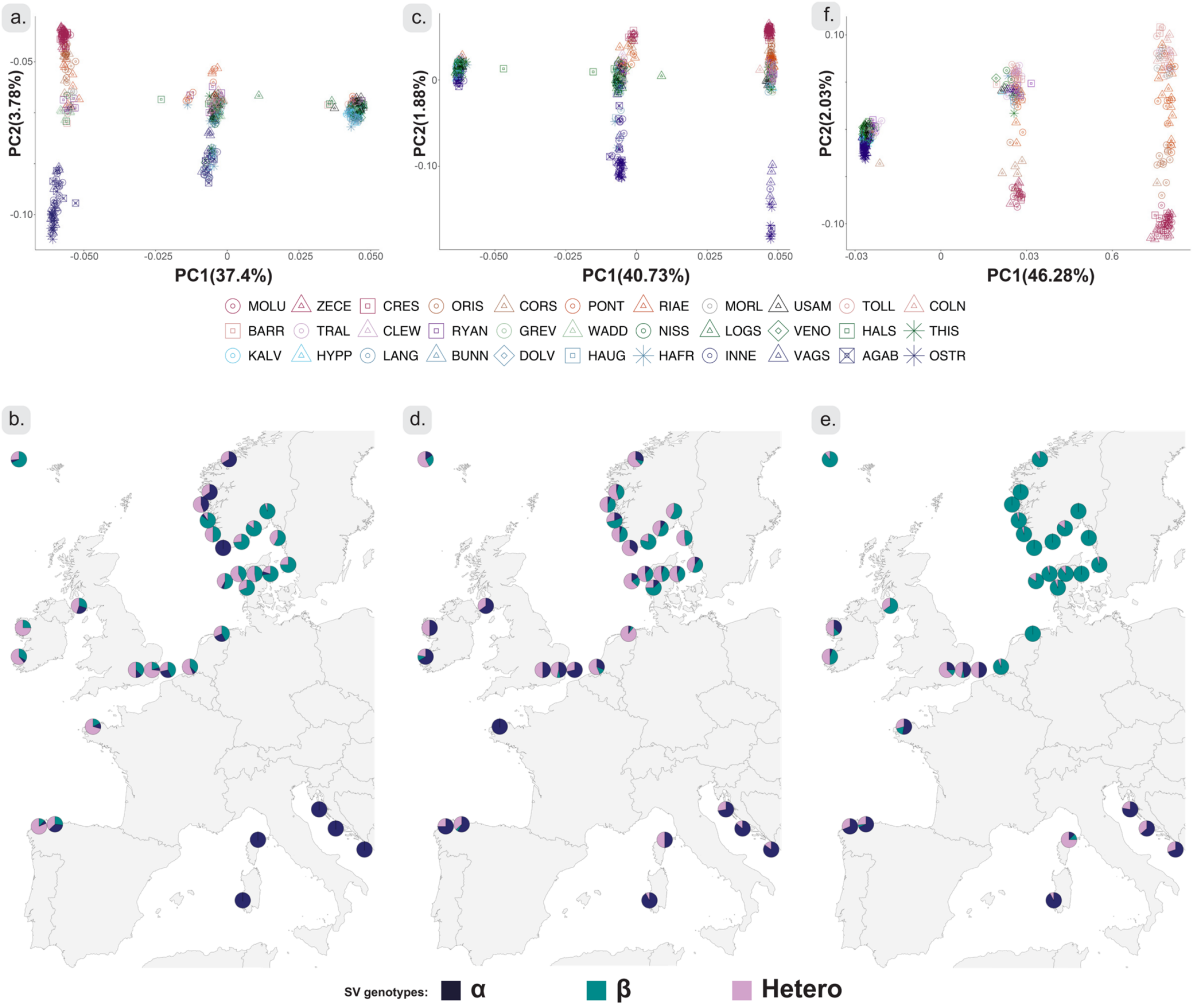
PCAs and genotype frequency distributions for three putative SVs. Clustering of individuals by PCA of SNPs located in putative SVs for (a) pseudo‐chromosome 4 (“Chr04:22Kb_sv”), (c) pseudo‐chromosome 5 (“Chr05:172Kb_sv”) and (e) pseudo‐chromosome 8 (“Chr08:33800Kb_sv”), and geographical distribution of SV genotype frequencies for (b) pseudo‐chromosome 4 (“Chr04:22Kb_sv”), (d) pseudo‐chromosome 5 (“Chr05:172Kb_sv”) and (f) pseudo‐chromosome 8 (“Chr08:33800Kb_sv”). For (a), (c), and (e), sampling site labels are placed at the central point, calculated as the statistical center of individuals from the same sampling sites. For pie charts in (b), (d) and (e), SV genotype “α” indicate the homozygous group with the most diversity following PC2, whereas “β” genotype is the less diverse homozygous genotype, for each SV respectively. “Hetero” SV genotype is assigned for the middle cluster on PC1. Framed pie charts in the top left corners correspond to the ‘USAM’ sampling site.

PCAs for each SV resulted in the formation of three distinct clusters on PC1, consistent with putative polymorphic chromosomal rearrangements and the genotypic combinations of two alleles (Figure 4). For each SV, three genotypes were inferred: a more diverse homozygous genotype, a heterozygous genotype and a less diverse homozygous genotype, as defined based on variation along PC2. We label the more diverse SV allele as “α” and the less diverse SV allele as “β” throughout. Along PC2, homozygous genotype clusters showed alternate strong differentiation between sampling sites (Figure 4). In “Chr04:22Kb_sv”, PC2 displayed strong differentiation between Norwegian sites “AGAB”, “INNE”, “VAGS”, “OSTR” and other sampling sites for the “α” homozygote genotype. A similar pattern was found for the “α” homozygote genotype in “Chr05:172Kb_sv”, although more nuanced as fewer Norwegian individuals had this genotype. The clear divergence of samples from the Norwegian west coast on PC2 for “Chr04:22Kb_sv” (Figure 4a) and “Chr05:172Kb_sv” (Figure 4c) allows for the identification of the presence of potential “non‐native” SV alleles in individual oysters. For “Chr04:22Kb_sv” “α/β” heterozygotes (central cluster on PC1), a number of individuals from other Scandinavian sampling sites grouped clearly with the divergent Norwegian populations on PC2 (green and blue individuals among the purple individuals in the central cluster, Figure 4a). For “Chr05:172Kb_sv” the reverse pattern was visible, i.e. individuals from the divergent Norwegian clusters grouping in the Scandinavian cluster (Figure 4c). Thus, while these individuals were clearly heterozygous for the SV alleles, they seemed to carry the alleles associated with the other genetic cluster. Finally, for both “Chr04:22Kb_sv” and “Chr05:172Kb_sv”, some heterozygous individuals were located between the two clusters on PC2 (Figure 4a,c), suggesting that they carried one copy from each of the genetic clusters. In all cases, individuals carrying the alternate allele were found in Scandinavian sampling sites. The patterns observed for the SV “Chr08:33800Kb_sv” was different as the “α” homozygotes were absent from Scandinavia and divergence along PC2 thus primarily observed among other European sampling locations, in particular a separation of Mediterranean sampling sites (Figure 4e). For this SV, divergence between Scandinavian sampling sites was less pronounced and observed for the “β” homozygote genotype (Figure 4e).

Further geographical structure was revealed in the geographical distribution of the SV genotypes. Thus, all Mediterranean sites harboured almost exclusively the “α” homozygote genotype for “Chr04:22Kb_sv” (Figure 4a,b). For “Chr08:33800Kb_sv” all Scandinavian sites harboured almost exclusively the “β” homozygous genotype while this was at lower frequencies in other geographical regions and almost absent (except from the Corsican sampling site) from the Mediterranean. For “Chr05:172Kb_sv”, both alleles were more frequent across geographic regions. For “Chr04:22Kb_sv” and “Chr05:172Kb_sv”, some Scandinavian sites shared genotypes with other European sampling sites, including those from the Mediterranean (Figure 4). No sampling site deviated significantly from HWE proportions for SVs (except “WADD” and “PONT” sampling sites for “Chr05:172Kb_sv” and “Chr04:22Kb_sv”, respectively (Table S3)).

Mantel tests showed that genetic structure was significantly associated with geographic distance for all SVs (Figure S8e,i,m). While genetic structure for the SVs was significantly correlated with that estimated with for genome wide data (Figure S8f,g,j,k,n,o), both the geographic distribution of genotype frequencies (Figure 4) and patterns of pairwise F_ST_ (Figure S8) were different for the three SVs. These differences were also reflected in different associations between SV and genome wide structure (Figure S8).

Absolute genetic divergence (d_xy_) was reduced for polymorphic sites inside SV regions in comparison to the rest of the pseudo‐chromosome (Figure S16). The general trend was that absolute divergence (d_xy_) and heterozygosity (Figure S17) estimation within and between “α” homozygous genotypes for each SV were similar to the ones found for “β” homozygous genotypes, preventing the clear identification of ancestral and derived arrangements between homozygous genotypes through the analyses of sequence divergence and heterozygosity data.

## 4 Discussion

By analysing variation across millions of SNPs for hundreds of *O. edulis* genomes spanning the species' geographical range, we provide the first detailed examination of the large‐scale genomic population structure of the species. Below, we discuss our main findings in detail with the aim of improving our understanding of *O. edulis* evolutionary patterns and our knowledge base for the management and restoration of the remnant wild European oyster populations.

### 4.1 Genome‐Wide Population Structure

The increased resolution from whole‐genome sequencing and the addition of sampling sites that have not been covered previously, confirms the genetic structure in *O. edulis* from earlier studies (Lapègue et al. 2022; Launey 2002; Vera et al. 2016), and also identifies previously undescribed genetic heterogeneity in Scandinavia, represented by samples collected from multiple sampling sites along the Norwegian coastline. Similar to results in our study, one of these sampling sites was recently found to be differentiated from other Scandinavian sampling sites in a regional study with high sampling density (Robert et al. 2024). Apart from the Scandinavian clusters, our samples display a geographical pattern of structuring with increased divergence from the innermost Mediterranean to the Atlantic, like described in previous work (Figure 1; Figures S6 and S8). The clinical genetic differentiation was reflected in significant patterns of isolation by distance, both in‐ and excluding the Mediterranean populations from analyses (Figure S8).

Our dense sampling in Scandinavia provides novel insights into the genetic relationships among *O. edulis* populations in the region. We found a close genetic relationship between the southern North Sea and samples from the species' northern distribution in Denmark, Sweden and Norway but also two highly distinct population clusters within Norway, showing genome‐wide differentiation from other genetic clusters (Figure 1; Figures S6, S8a, S14c,i). Interestingly, these two genetic clusters exhibit a patchy distribution along the Norwegian coast with the most distinct sampling site (OSTR) geographically interspaced among samples representing the other Scandinavia cluster. This distribution pattern resembles the mussel complex *Mytilus* spp. distribution observed along European coasts (Bierne et al. 2003; Simon et al. 2020). Specifically in *M. trossulus* introgression along the Norwegian west coast exhibits a patchy distribution similar to what we observe here in *O. edulis* (Wenne et al. 2020; Gustafsson et al. 2024). Nonetheless, more detailed geographical sampling is needed to confirm whether there is parallelism between the population structure of the two species' and to examine the extent to which patterns could be linked to past translocations among Norwegian coastal sites (Bromley et al. 2016; Mortensen et al. 2017). However, the apparent genetic break between the two clusters in southwestern Norway is co‐localised with previously documented genetic breaks in other marine species. For example, a major phylogeographic break in corkwing wrasse (*Symphodus melops*) was found between the west and south coast of Norway, and a second, weaker break at around 62° N on the Norwegian west coast (Blanco Gonzalez et al. 2019; Faust et al. 2021; Mattingsdal et al. 2020). The genetically highly distinct OSTR oysters (Figure S14i) were collected from an isolated lagoon (“poll”) with limited connection to the marine environment except under extreme weather conditions. It is thus possible that they represent a small and isolated population subject to strong genetic drift. Although population genetic estimators in an lcWGS framework should be interpreted with caution (Lou et al. 2021), this is further supported by low nucleotide diversity and Watterson's *θ* values, as well as high Tajima's *D*, compared to other sampling sites (Figure S12b). In addition, this sampling site displayed reduced genome‐wide heterozygosity (Figure S12a). The OSTR location has a history involving production and translocation activities, including sourcing of individuals for other Scandinavian localities (Mortensen et al. 2017; Mortensen et al. 2022; see also below). It is thus also possible that the establishment of the population was originally linked to human mediated activity associated with oyster production.

The high levels of divergence across short geographic distances suggest strong barriers to gene flow, as also supported by the absence of first‐generation hybrids among our sampled individuals from Norwegian sampling locations (Figure 1c). Yet, indications of introgression at some sampling sites (DOLV, HAFR and to a lesser extent HAUG), with intermediate positions between the Scandinavian cluster and the divergent Norwegian populations suggested potential signatures of admixture between these populations (Figure S7c). The sampling sites with indications of introgression were the ones in closest physical proximity, suggesting that patterns may indicate natural introgression between the genetically divergent genetic clusters.

The mitochondrial genomes showed highly distinct haplotype clusters (Figure S11). However, unlike for the genomic data, the mitochondrial structure was not clearly related to geography or to the clusters identified with nuclear genomes, showing mixed haplotype composition across most geographical regions. This pattern may indicate secondary contact and admixture of divergent populations. Similar broad scale discordant signals for genomic and mitochondrial data have been observed in nine‐spined stickleback in Scandinavia (Feng, Merilä, and Löytynoja 2022), and secondary contact has been inferred as potentially important for structuring *O. edulis* populations (Lapègue et al. 2022). Thus, the genomic data in our study may reflect evolutionary forces acting on more recent time scales among populations composed of mixed gene pools from once isolated lineages. Interestingly, the reconstructed demographic histories did not reveal major differences between the seven sequenced individuals representing the main genomic and mtDNA clusters (Figure S11). This result is in contrast to other studies of marine taxa from the same geographic regions (e.g., Mattingsdal et al. 2020; Jaspers et al. 2021), and it may indicate common demographic trajectories for all populations despite their isolation at both shorter and longer time scales. However, it is also possible that these results are affected by recent population admixture and gene flow, which violates assumptions underlying the applied methodology (Mather, Traves, and Ho 2020; Nadachowska‐Brzyska et al. 2016). Consequently, the demographic reconstruction should be interpreted with caution.

### 4.2 Large Structural Variants

The data indicated the presence of three large structural variants with characteristics suggestive of chromosomal inversions, as have also been observed in several other marine taxa (e.g., Akopyan et al. 2022; Hess et al. 2020; Han et al. 2020; Le Moan, Bekkevold, and Hemmer‐Hansen 2021; Le Moan et al. 2023; Wooldridge et al. 2024) including in an oyster species (*Crassostrea virginica*: Puritz et al. 2022). The putative structural variants in the European flat oyster are characterised by high LD (Figure S13) with well‐defined genotypes segregating among and within sampling sites. The *O. edulis* SVs are large (range of size: 7.8–24.5 Mb; Table 2) but still comparable to inversions in other marine species (e.g., Atlantic silverside: 0.4–12 Mb Akopyan et al. 2022, cod: 4‐17Mbp, Matschiner et al. 2022, and Atlantic herring: 7.8 Mbp, Pettersson et al. 2019).

The three putative SVs showed different geographical distribution patterns. Within Atlantic samples, there were clear differences between southern and northern populations, and patterns, although positively related (Figure S8), did not seem to be clearly aligned with those estimated from genome wide data. The patterns of SV allele frequency distributions can be explained by neutral and/or non‐neutral evolutionary processes. For example, the action of one or several interacting evolutionary forces could explain the patchy SV distribution along the Norwegian coast. Each of the putative SVs carry hundreds of genes (Table 2), and natural selection on any of these could be driving frequency changes of the SV alleles in local populations (Figure 4).

An alternative, and not mutually exclusive, hypothesis is that SVs are involved in intrinsic reproductive barriers to hybridization between divergent populations (Bierne et al. 2003; Simon et al. 2020), acting as “barrier loci” that may even become “trapped” in environmental transition zones and hence difficult to distinguish from loci involved in adaptation to local extrinsic environmental factors (Bierne et al. 2011). The shared allele frequency patterns for “Chr04:22Kb_sv”, and to some extent “Chr05:172Kb_sv”, between Northern and Southern edges of the species distribution (Figure 4) could indicate effects from secondary contact, as also suggested in a recent study comparing North Sea with Black Sea‐Eastern Mediterranean populations (Lapègue et al. 2022). Those sampling sites were, however, not included in our study. The outlier LD blocks in Lapègue et al. (2022) align to “Chr05:172Kb_sv” and “Chr08:33800Kb_sv”, in our study. In our study, a shared evolutionary ancestry only seems compatible with one of the three putative SVs, “Chr04:22Kb_sv”, whereas genotype distributions for “Chr05:172Kb_sv” and “Chr08:33800Kb_sv” in the Northern populations are more similar to other Atlantic populations, than to the eastern Mediterranean (Figure 4). Analyses of sequence divergence between the two distinct homozygous genotypes did not provide conclusive results, preventing a clear identification of ancestral and derived alleles for the SVs (Figure S16). The lack of clear difference in diversity for the SV alleles may also indicate that the three segregating SVs are old and have accumulated diversity since their origin.

In contrast to many other marine species where genomic regions outside SVs are characterised by low levels of structure indicative of high levels of gene flow (Han et al. 2020; Wilder et al. 2020; Matschiner et al. 2022), the highly divergent Norwegian population is characterised by genome‐wide divergence from neighbouring populations (Figure S14i), suggesting effects of genetic drift. Thus, a third hypothesis for the divergent SV patterns is exposure to genetic drift, which could also explain the discordant genotype distributions between SVs discussed above. Although genetic drift may be important, this hypothesis is of course not excluding effects from selection in response to intrinsic and extrinsic factors.

### 4.3 Genetic Impact of Translocations

We found that population structure overall follows expectations under an isolation by distance model coupled with single large genetic breaks in specific geographical areas. Thus, there was little evidence that genetic signatures of local populations deviated from local‐ to large‐scale expectations, suggesting that translocations with genetically divergent strains were generally not successful on a medium‐ to long time scale. We note, however, that we did not include samples from a Mediterranean locality previously found to display genetic signs of translocations (Lapègue et al. 2022). Large‐scale effects from the release of hatchery‐produced oysters could also be expected to result in the increased frequency of related individuals in the sampled oyster beds, something we only observed in a few localities (Figure S9).

Although there was limited evidence for large‐scale population effects of past translocation, the high divergence of alleles within SVs (second PC axes in Figure 4) allows for more detailed investigation of potential traces of introgression in individual genomes. For “Chr04:22Kb_sv” and “Chr05:172Kb_sv”, some individuals from Scandinavian sampling sites carried one or two alleles from the other major Scandinavian cluster. These results suggest genomic traces of introgression among the two major Scandinavian genetic clusters. However, signals of introgression for the SVs involved sampling sites not only in close physical proximity as expected for signatures associated with natural dispersal and introgression. For example, we observed traces of ancestry from the divergent Norwegian clusters in Danish and Swedish sampling sites (Figure 4). Such patterns are consistent with some of the known historical translocations of oysters, for example from Ostretjønn (“OSTR”) in Norway to Limfjorden in Denmark (Mortensen et al. 2017; Bøgwald and Mortensen 2024). Thus, although we do not observe large scale effects of translocations and we cannot exclude the occurrence of long‐distance natural dispersal and introgression or effects from shared ancestral variation, our data also indicate that previous translocations may have left signatures in recipient populations through genetic introgression and that translocations have the potential to alter natural genetic diversity in the species.

The production of *O. edulis* in North America is based on relatively few translocations of oysters from the Netherlands (Bromley et al. 2016). In the state of Maine, from where our U.S. sample originated, production is thus based on oysters introduced to Boothbay Harbour from the Netherlands (Oosterschelde) in 1949, supported by a local hatchery production (Loosanoff, 1955). This historical record is consistent with the genomic pattern we observed.

### 4.4 Management Implications

The overall structure in our data is consistent with natural migration‐drift dynamics, indicating limited large‐scale effects from past translocations, at least between the major genetic clusters identified in our study. This result is in contrast to earlier finding of substantial effects from past translocations in the species (Lapègue et al. 2022). Our results indicate that sourcing non‐local genetic variants for restoration is an ineffective procedure, perhaps due to mal‐adaptation. However, while we did not see clear population scale effects we found individuals with traces of introgression potentially linked to past translocations in natural oyster populations. Consequently, even if stocking is not efficient from a production point of view, it may still result in maladaptive genetic changes through introgression into native populations (Laikre et al. 2010). Effects could be further amplified through swamping effects, which may be exacerbated if hatchery produced sourcing material exhibits low genetic diversity (Alves Monteiro et al. 2022), and exhibits mismatch with natural genetic breaks (Waples et al. 2016). The genome‐wide based geographical delineation of populations provided here allows for the definition of conservation units with a more accurate resolution, compared with the studies based on the limited genome‐wide representation (Bernatchez et al. 2017). In the context of *O. edulis* conservation, our data are relevant to support decision‐making in the large number of restoration initiatives which typically face challenges related to the selection of source populations, especially in cases where local populations are extinct or at very low densities (Fariñas‐Franco et al. 2018; Hughes et al. 2023; Pogoda et al. 2019). Our delineation of natural genetic structure is useful for determining geographic regions where appropriate source populations can be used for restoration, to minimise negative effects of introducing maladapted source material into locally adapted recipient populations. To safeguard local evolutionary dynamics, any translocations should, at a minimum, be restricted to within each of the genetically distinct clusters. However, we also note that our sampling design was not very dense in some geographical regions. Thus, future work could extend the sampling coverage to provide finer scale delineation of genetic breaks in the species. Current biosecurity protocols in the species are highly focused on the prevention of disease spread and invasive species through translocations (zu Ermgassen et al. 2020b), and our results suggest that these guidelines should be supplemented with knowledge about the distribution of natural genetic diversity in *O. edulis*.

Genetic and evolutionary rescue has been increasingly recognised as viable strategies for increasing genetic diversity and population viability (Fitzpatrick et al. 2020; Frankham 2015; Oziolor et al. 2019). We found only limited evidence for drastically reduced diversity and no clear signal of severe inbreeding in the studied populations. While more focused analyses and temporal data would be useful for a thorough evaluation of inbreeding and climate change vulnerability, our results indicate that genetic rescue would not be immediately needed as a conservation strategy for the majority of populations included in this study. However, on longer time scales, for example in relation to buffering populations against the impacts of climate change, enhancing and supporting genetic diversity in local populations through restoration should be a relevant strategy and an integrated aim in hatchery management. As such, our results highlight the necessity to have genetic information regarding both receiving and donor populations for genetic management of *O. edulis* wild remnant populations.

The genetic basis of resistance to the harmful parasite *B. ostrea* is under scrutiny due to its severe impact on wild and cultured stocks (Culloty, Cronin, and Mulcahy 2004; Madsen, Kamp, and Mellergaard 2013; Madsen and Thomassen 2015; Sas et al. 2020). Resilience to the parasite has been linked to a genomic region (Sambade et al. 2022) associated with “Chr08:33800Kb_sv”, and consequently to variants we found to be almost fixed in Scandinavian sampling sites. However, while Norway and Sweden have not been impacted by severe disease outbreaks, the disease has been found in the Danish Limfjorden (Madsen and Thomassen 2015). In addition, the two sampling sites from the Netherlands, which both had high frequencies of the same SV allele, are considered to be, respectively, “naïve” to bonamiosis (Wadden Sea), and ‘long‐term affected’ (Grevelingen; Sambade et al. 2022). Thus, overall there is not a clear link between the contemporary distribution of putative resistance alleles and the disease status of Wadden Sea‐Scandinavia populations. As mentioned previously, it is also likely that neutral evolutionary forces and selection on any of the hundreds of genes in “Chr08:33800Kb_sv” in response to other drivers, could be responsible for shaping geographical patterns of diversity for this particular genomic region. Importantly, we found the “Chr08:33800Kb_sv” to segregate at most sites in the Atlantic. This suggests that important disease‐resistance related diversity may be present as standing variation to support resilient restoration approaches and perhaps provide the basis for marker assisted selection at local scales.

## 5 Conclusion

This study has shown the potential for integrating genomic information with practical management and conservation in a keystone species in coastal marine ecosystems. Future restoration in coastal ecosystems will benefit significantly from the detailed evolutionary and ecological insights provided by high resolution genomic data that will become increasingly available to management and restoration practitioners in the coming years. We recommend that conservation and restoration in *O. edulis* takes current knowledge about the natural distribution of genome‐wide diversity in the species into account and that future studies add further detail on spatially explicit genomic diversity to maximise genetic suitability of stocking material with local conditions. In addition, knowledge about genetic effects of production and restoration practices will also be important when implementing restoration protocols involving sourcing of hatchery produced material (Alves Monteiro et al. 2022). Data could come from controlled experimental set‐ups and from monitoring ongoing restoration projects to determine genetic effects on natural populations.

## Author Contributions

Funding acquisition: J.H.‐H., D.B., C.S., and Å.S. Conceptualization: J.H.‐H., D.B., C.S. and H.J.A.M. Sampling: H.J.A.M., C.S., S.M., P.D.W., A.T., A.L., Å.S.Molecular laboratory: D.M. and H.J.A.M. Bioinformatics: H.J.A.M. with inputs from J.H.‐H., G.P., D.B., N.R.L. and N.T. Methodology: H.J.A.M., J.H.‐H. and D.B. Manuscript writing first draft: H.J.A.M., J.H.‐H. and D.B. with inputs from all the coauthors. Manuscript proofing: All the coauthors.

## Conflicts of Interest

The authors declare no conflicts of interest.

## Supporting information


Figures S1.
Figure S2.
Figure S3.
Figure S4.
Figure S5.
Figure S6.
Figure S7.
Figure S8.
Figure S9.
Figure S10.
Figure S11.
Figure S12.
Figure S13.
Figure S14.
Figure S15.
Figure S16.
Figure S17.

Data S1.

Tables S1.
Tables S2.
Tables S3.

## Data Availability

Steps for the filtering and mapping to the genome are available in the repository https://github.com/HomereAMK/Shucking. lcWGS population genomic analyses: https://github.com/HomereAMK/EUostrea. High coverage (> 18×) population genomic analyses (PSMC): https://github.com/HomereAMK/HighCovOyster_preprocess. SRA data: BioProject PRJNA1029395.
