# Supplementary material for: Genome‐Wide Population Structure in a Marine Keystone Species, the European Flat Oyster (Ostrea edulis)
Source: Mol Ecol. 2024 Nov 12;34(23):e17573. doi: 10.1111/mec.17573 (PMC12684353; doi:10.1111/mec.17573)
Supplement: Supplementary file 1 — Figures S1. Figure S2. Figure S3. Figure S4. Figure S5. Figure S6. Figure S7. Figure S8. Figure S9. Figure S10. Figure S11. Figure S12. Figure S13. Figure S14. Figure S15. Figure S16. Figure S17. [file MEC-34-e17573-s002.docx]

**Supplementary figures**

Genome-wide Population Structure in a Marine Keystone Species, the European Flat oyster (*Ostrea edulis*).

Authors: Homère J. Alves Monteiro^1,2*^, Dorte Bekkevold^1^, George Pacheco^1,3^, Stein Mortensen^4^, R. Nicolas Lou^5,6^, Nina O. Therkildsen^5^, Arnaud Tanguy^7^, Chloé Robert^8^, Pierre De Wit^8, 9^, Dorte Meldrup^1^, Ane T. Laugen^10, 11^_,_ Philine S.E. zu Ermgassen^12^, Åsa Strand^13^_,_ Camille Saurel^14^, Jakob Hemmer-Hansen^1*^.

^1^National Institute of Aquatic Resources, Technical University of Denmark, Silkeborg, Denmark, Vejlsøvej 39, 8600, Silkeborg, Denmark

^2^ Section for Evolutionary Genomics, The Globe Institute, Faculty of Health and Medical Sciences, University of Copenhagen, Copenhagen K, Denmark

^3^Department of Biosciences, Centre for Ecological and Evolutionary Synthesis, University of Oslo, Oslo, Norway

^4^Institute of Marine Research, PO Box 1870 Nordnes, 5817 Bergen, Norway

^5^Department of Natural Resources and the Environment, Cornell University, Ithaca, New York, USA

^6^Department of Integrative Biology, University of California Berkeley, Berkeley, CA

^7^Sorbonne Université, CNRS, UMR 7144, Station Biologique de Roscoff, Roscoff, France

^8^Department of Marine Sciences, Tjärnö Marine Laboratory, University of Gothenburg, Laboratorievägen 10, 452 96 Strömstad, Sweden

^9^Department of Biological and Environmental Sciences, University of Gothenburg, Gothenburg, Sweden.

^10^Department of Ecology, Swedish University of Agricultural Sciences, Uppsala, Sweden

^11^Department of Natural Sciences, Centre for Coastal Research, University of Agder, Kristiansand, Norway

^12^Changing Oceans Group, School of Geosciences, University of Edinburgh, James Hutton Rd, King's Buildings, Edinburgh EH9 3FE, United Kingdom

^13^Department of Environmental Intelligence, IVL Swedish Environmental Research Institute, Kristineberg 566, 451 78 Fiskebäckskil, Sweden

^14^National Institute of Aquatic Resources, Technical University of Denmark, Danish Shellfish Centre, Øroddevej 80, 7900 Nykøbing Mors, Denmark

* Corresponding authors: jhh@aqua.dtu.dk; homerejalvesmonteiro@gmail.com

a.

b.

c.

**Fig. S1. LcWGS read depth per position for establishing SNP calling filters with ANGSD**

a. Our depth distribution exhibited a bell-shaped curve, with most positions having intermediate depths, and only a few positions having very high or very low depths.

b. We attempted to fit the standard deviation of the first and second peaks of the distribution to a normal distribution, but we did not arrive at a conclusive result.

c. Consequently, we applied a manual cut-off to the first and second peaks to set up the -setMinDepth **600**, setMaxDepth **1200** and filters for variant identification with ANGSD.


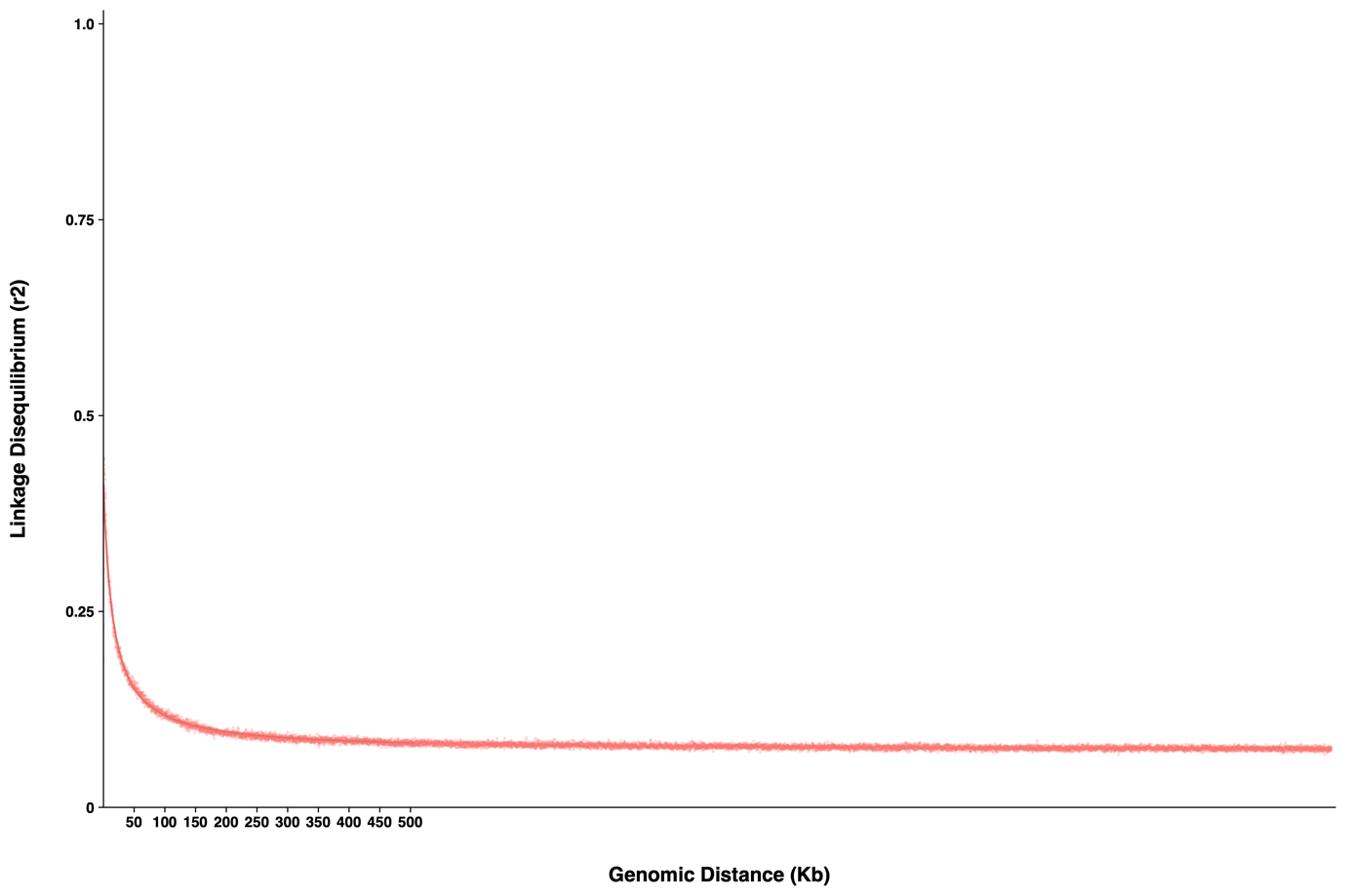


**Fig. S2. LD-decay curve.**

Genome-wide LD estimation and LD decay. LD is flattening around 100Kb.

a.

b.

c.


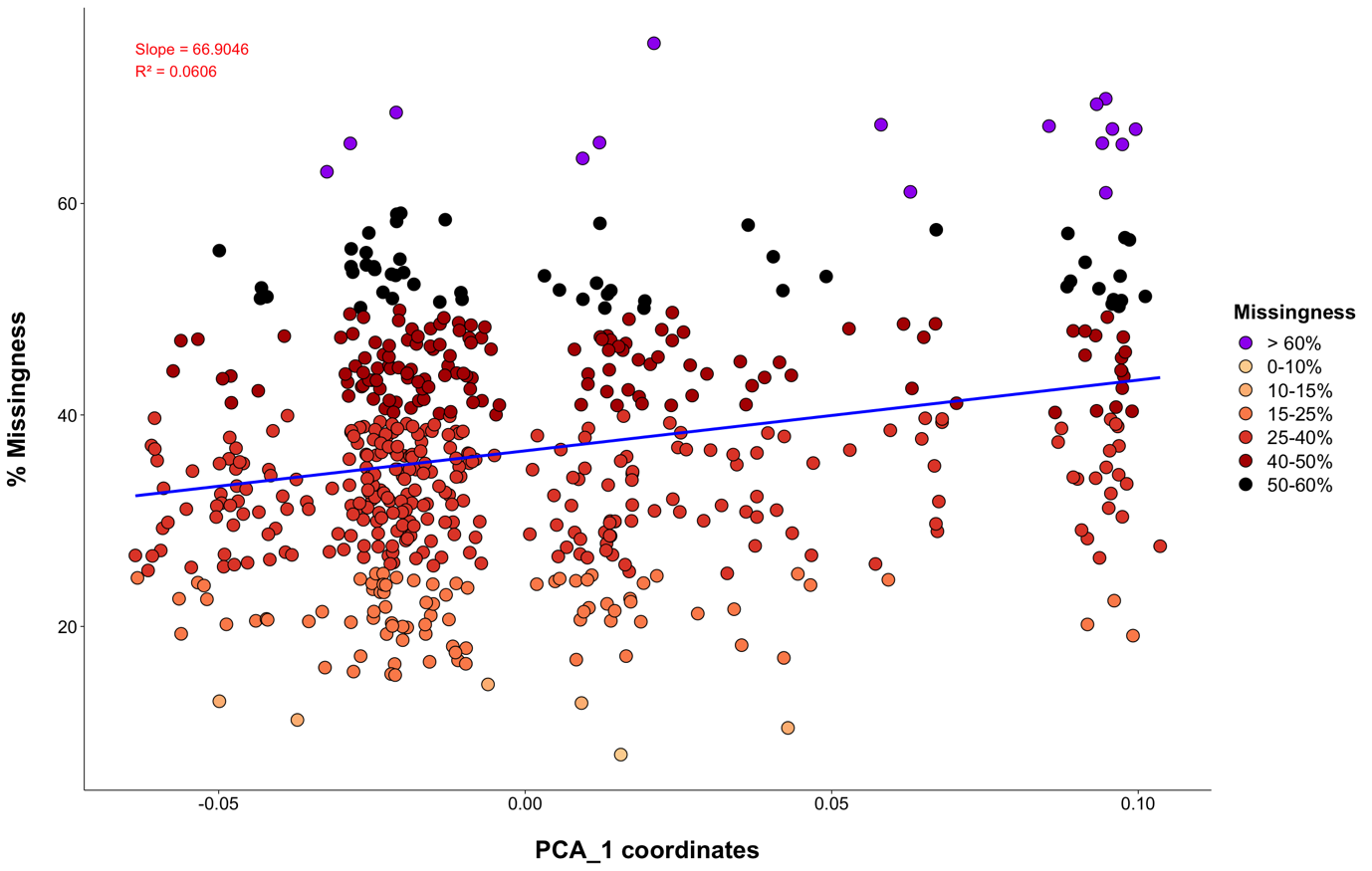


d.


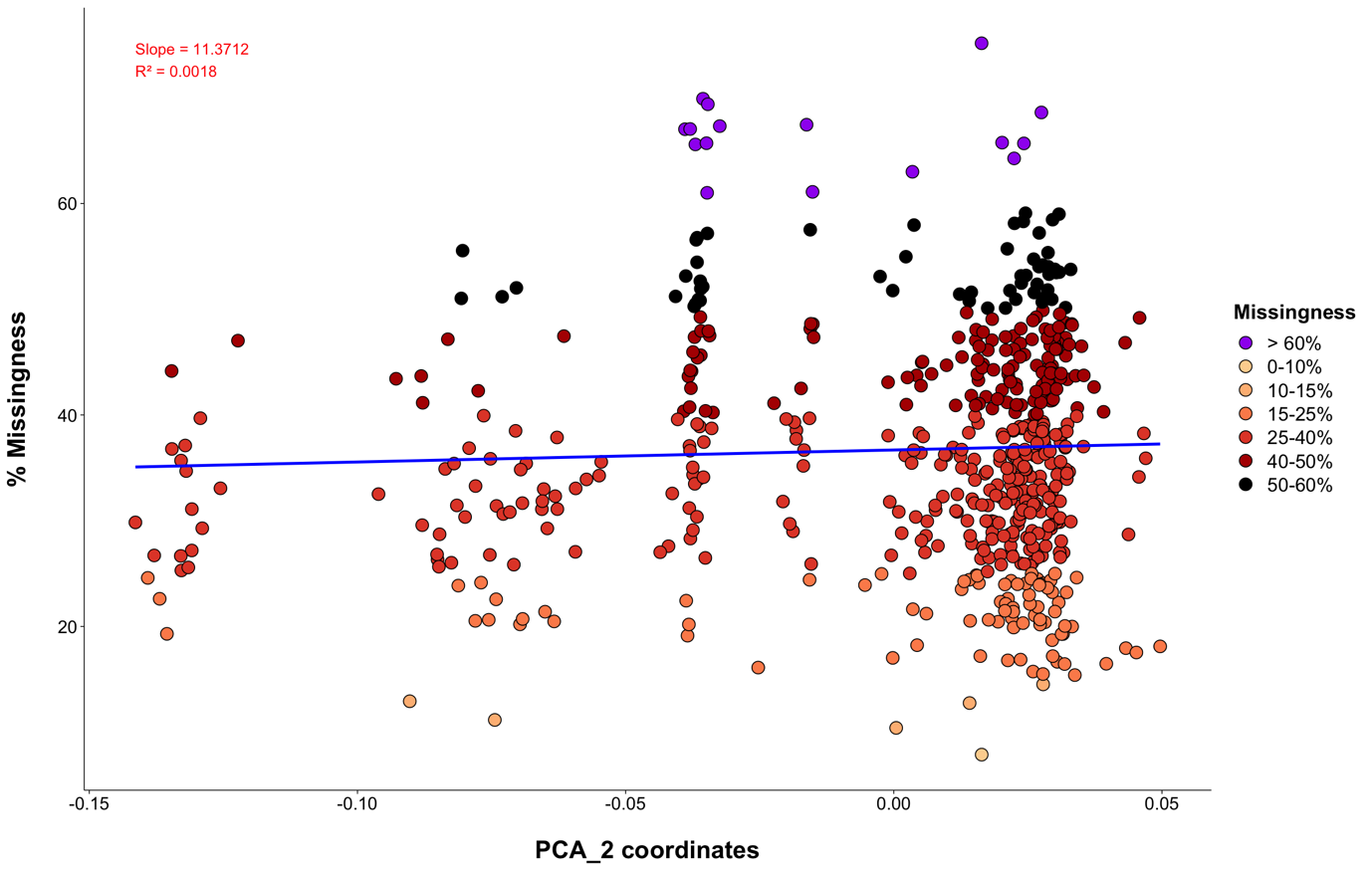


e.

**Fig. S3 Effects of missing data and batch effects on the PCA.**

*Rationale*: Principal component analyses with the distribution of missing data or sequencing batch across individuals to examine potential influences on the PCA analysis. Results showed no clear effects of missing data or batch effects on the PCA clustering.

a. PCA axes 1 and 2 with missingness shown per individual.

b. PCA axes 1 and 3 with missingness shown per individual.

c. Linear regression of PCA axis 1 coordinates and percentage of missing data across samples (R^2^=0.0606, *P* <0.001).

d. Linear regression of PCA axis 2 coordinates and percentage of missing data across samples (R^2^=0,0018, P=0.313).

e. PCA axes 1 and 2 with individuals colored by sequencing library batch of origin. “NA” represents individuals from “USAM” and “MORL” sequenced prior to our study and thus not included in any of the sequencing batches in the current study.

a.

b.

c.

d.

**Fig. S4. LcWGS Sequencing data processing and read depth.**

*Rationale*: Following data filtering and processing, we observed a loss of approximately 50% of the reads. The average depth of coverage was 1.3x, encompassing about 60% of the genome. Fourteen individuals were excluded from the study due to insufficient coverage, defined as less than 30% of their genome being covered.

a. Raw bases in the fastq files per sampling site.

b. Raw bases in the bam files after genome mapping, which included the removal of duplicated reads and realignment around indels, per sampling site.

c. Mean depth of coverage per sampling site.

d. Proportion of the genome covered per sampling site.

**
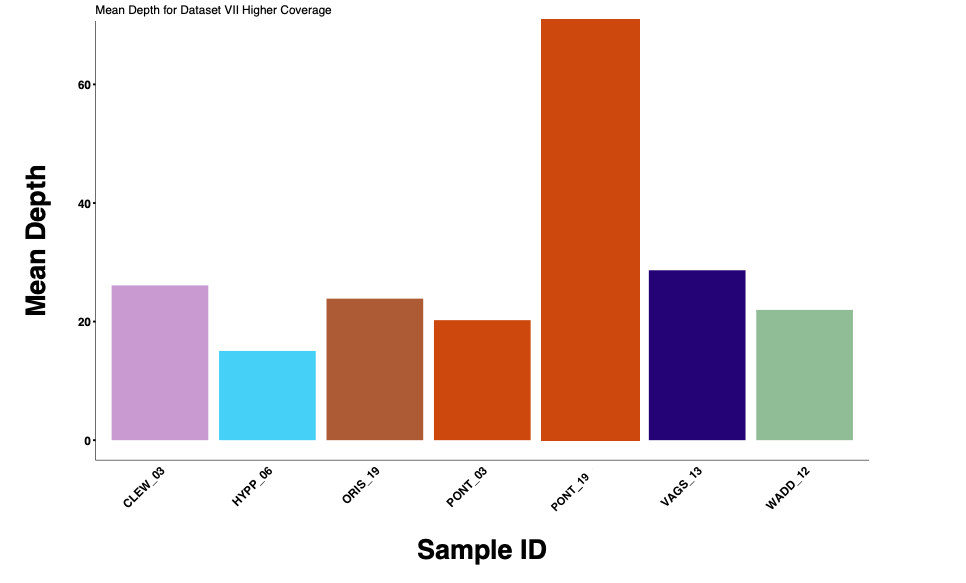
**

**Fig. S5. High coverage WGS sequencing mean read depth per individual.**

a.


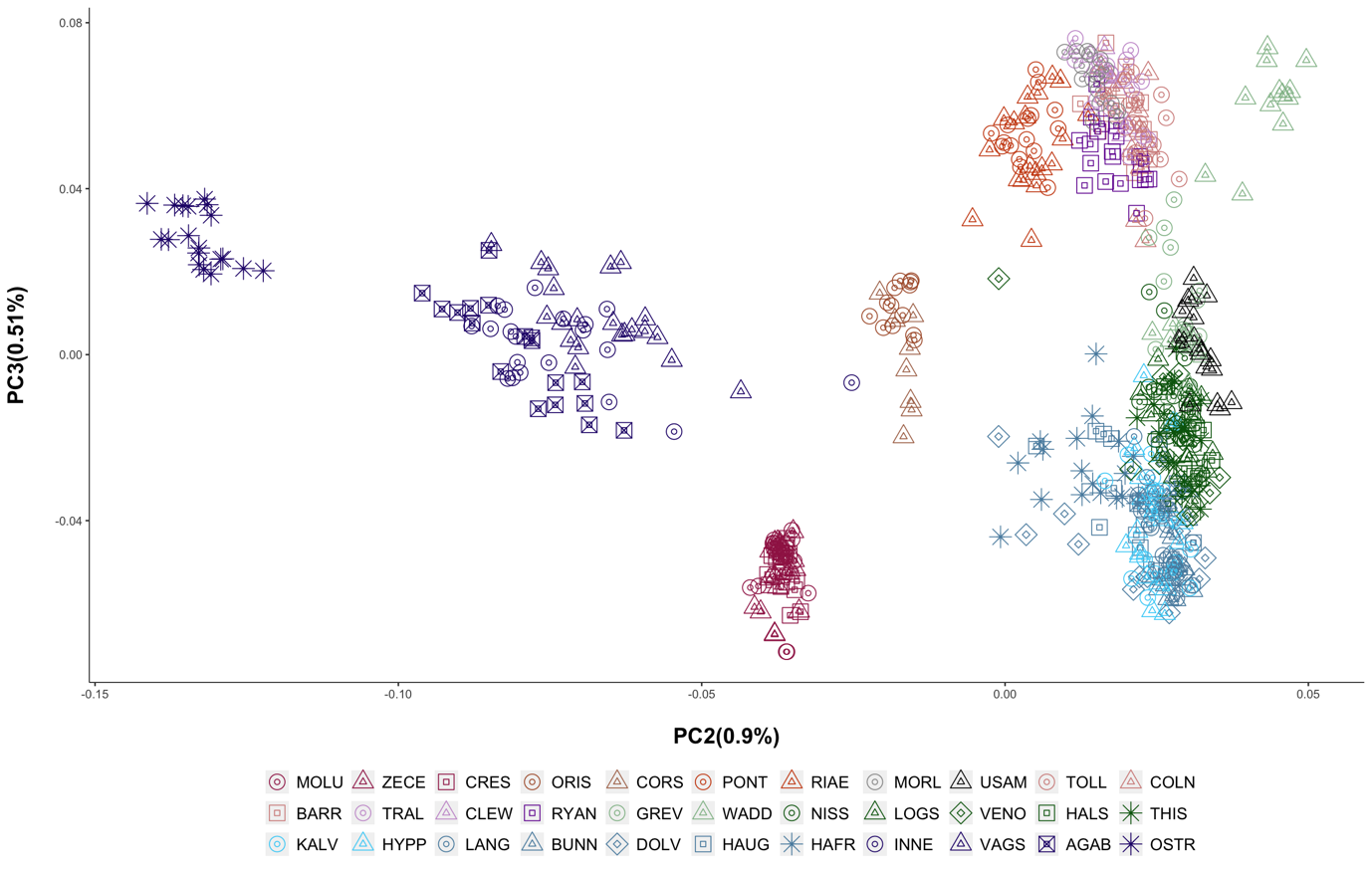


b.


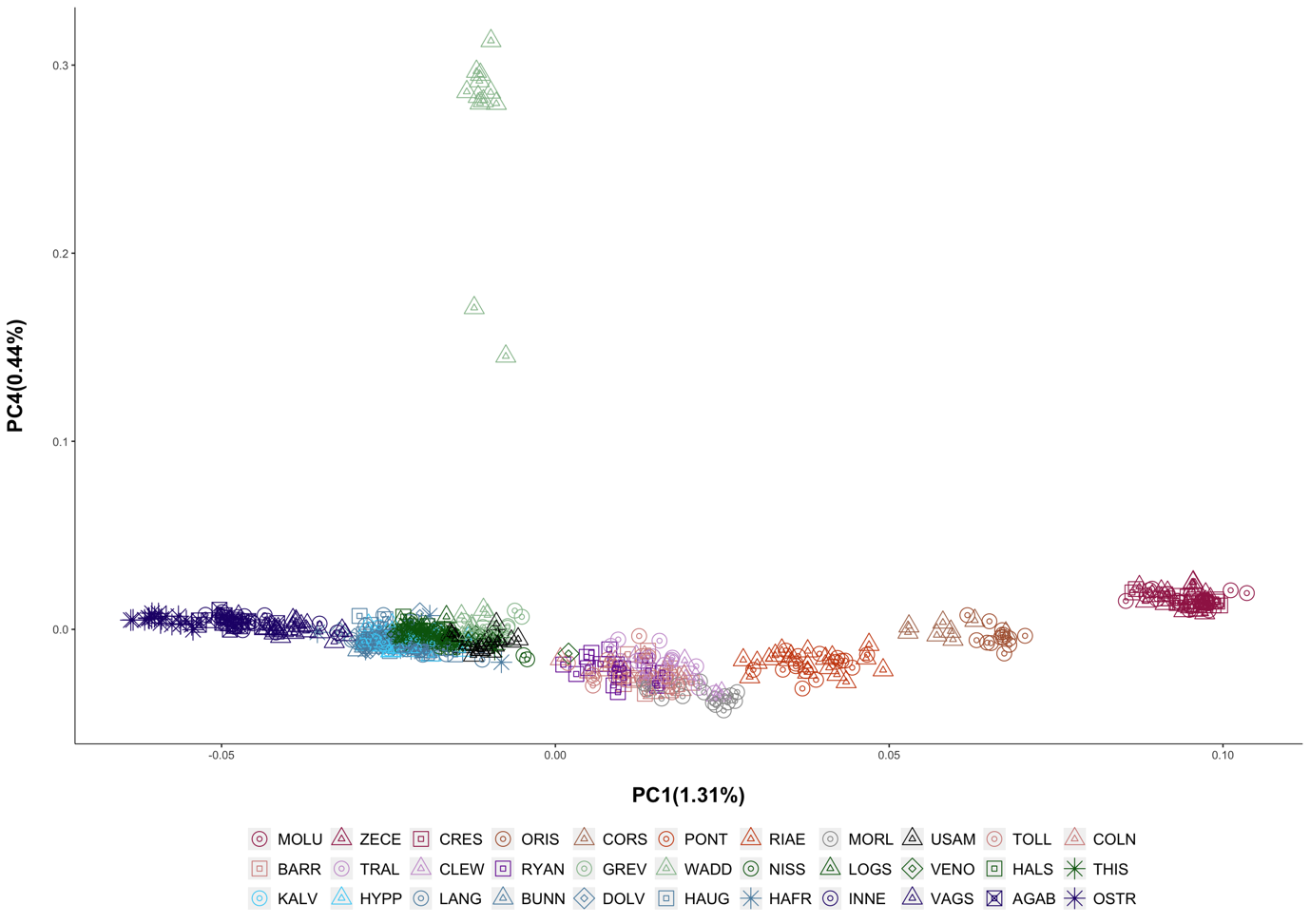


c.

**Fig. S6. LD-pruned SNPs PCA of PC1 vs PC4 and PCA without chr4, 5 and 8.**

*Rationale*: This figure presents the Principal Component Analysis (axis 1 vs. 4) for the 33 sampling sites, using the LD-pruned SNPs dataset (Dataset II, LD pruned SNPs).

a. LD-pruned SNPs PCA of PC2 vs. PC3.

b. LD-pruned SNPs PCA of PC1 vs. PC4.

c. LD-pruned SNPs PCA of PC1 vs. PC2 without SNPs from pseudo-chromosome 4, 5 and 8.

a.

b.

c.

**Figure S.7 PCA and Admixture plots for all sampling sites (K=2-10) and for Scandinavian sites only (K=2-8).**

a. LD-pruned dataset (Dataset II) PCA with Scandinavian sampling sites only, PC1 vs. PC2.

b. LD-pruned dataset (Dataset II) Admixture plot for all 33 sampling sites (K=2-10).

c. LD-pruned dataset (Dataset II) Admixture plot with Scandinavian sampling sites only (K=2-8).

a.

b.


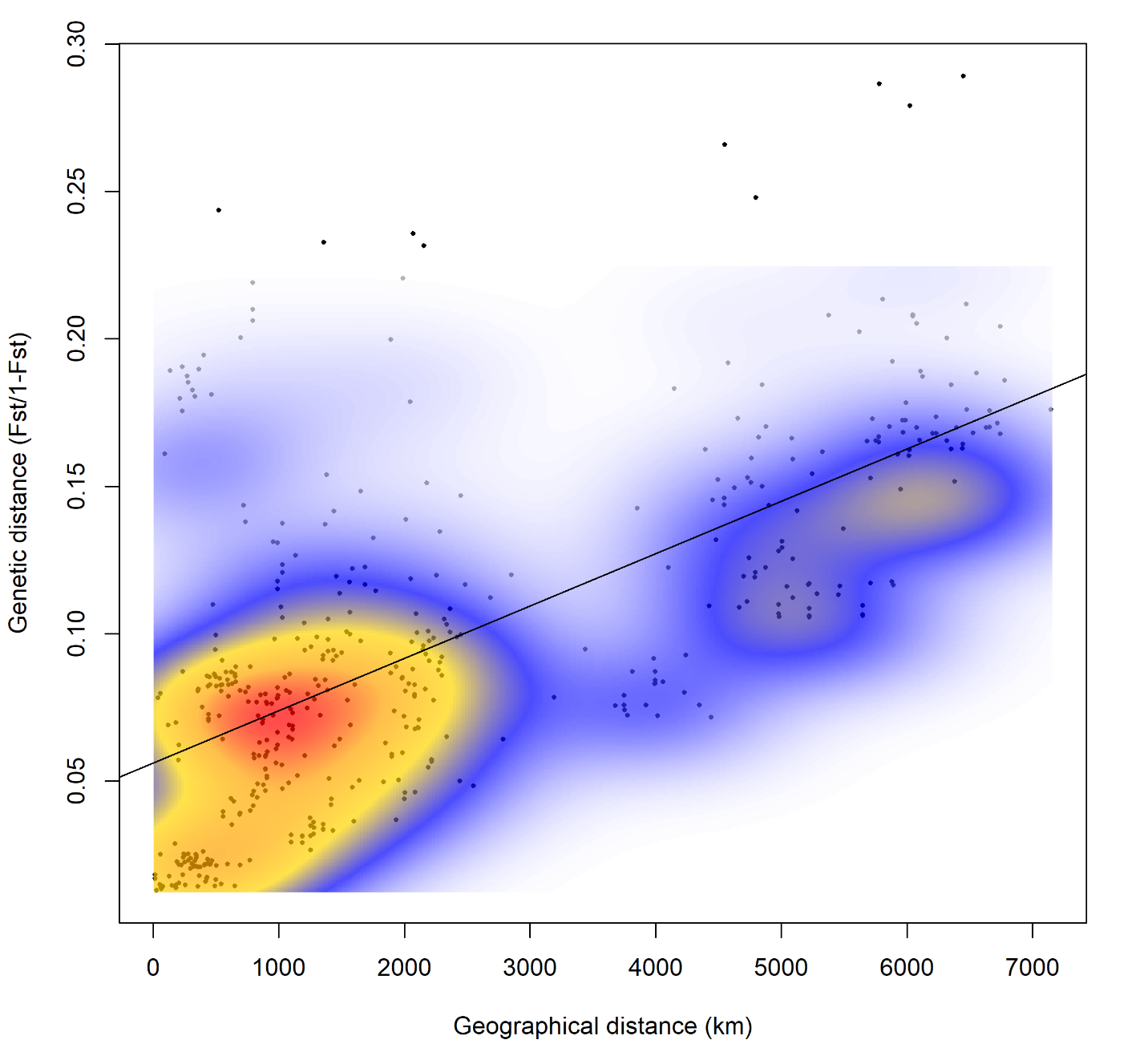


c.


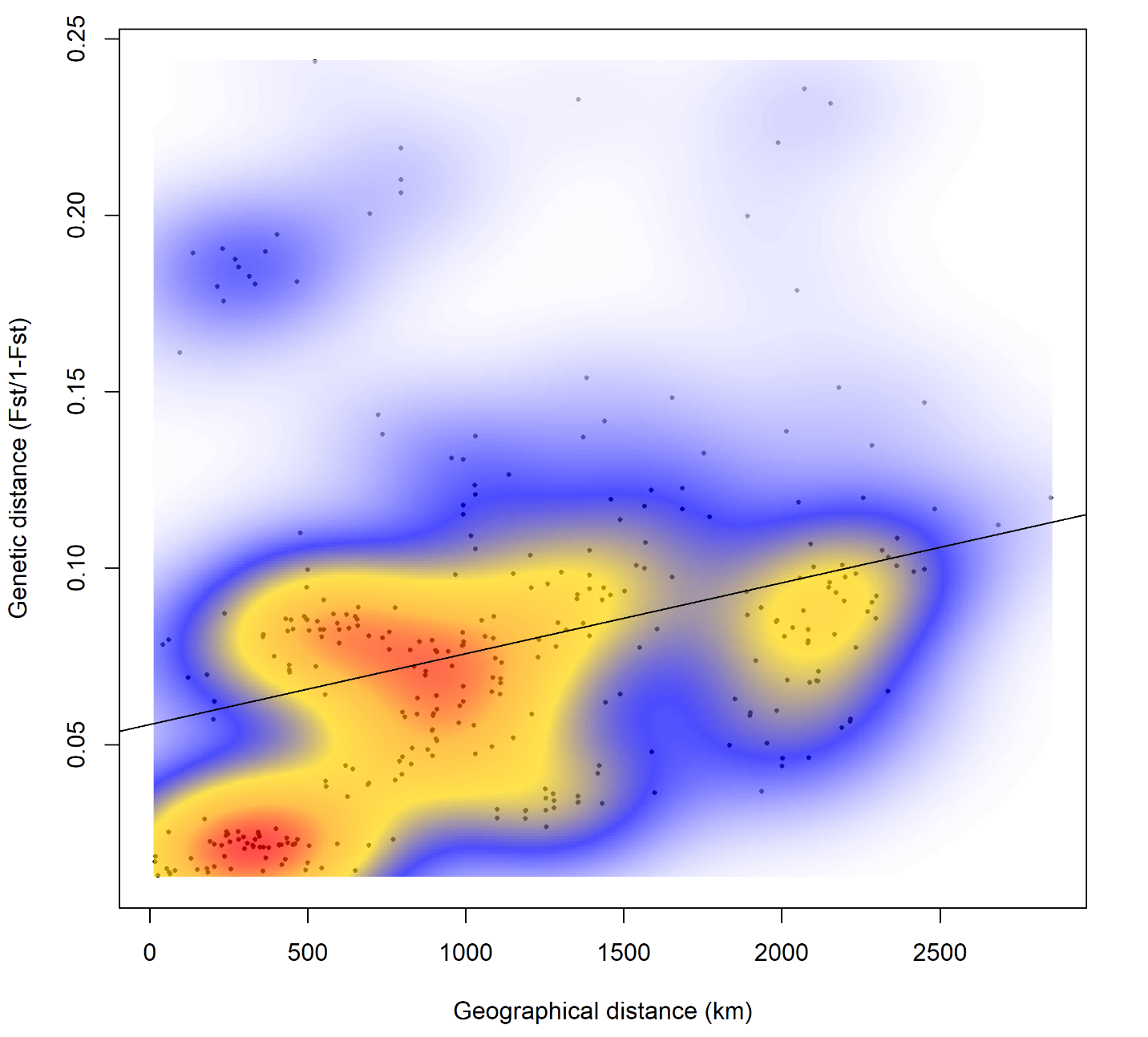


d.


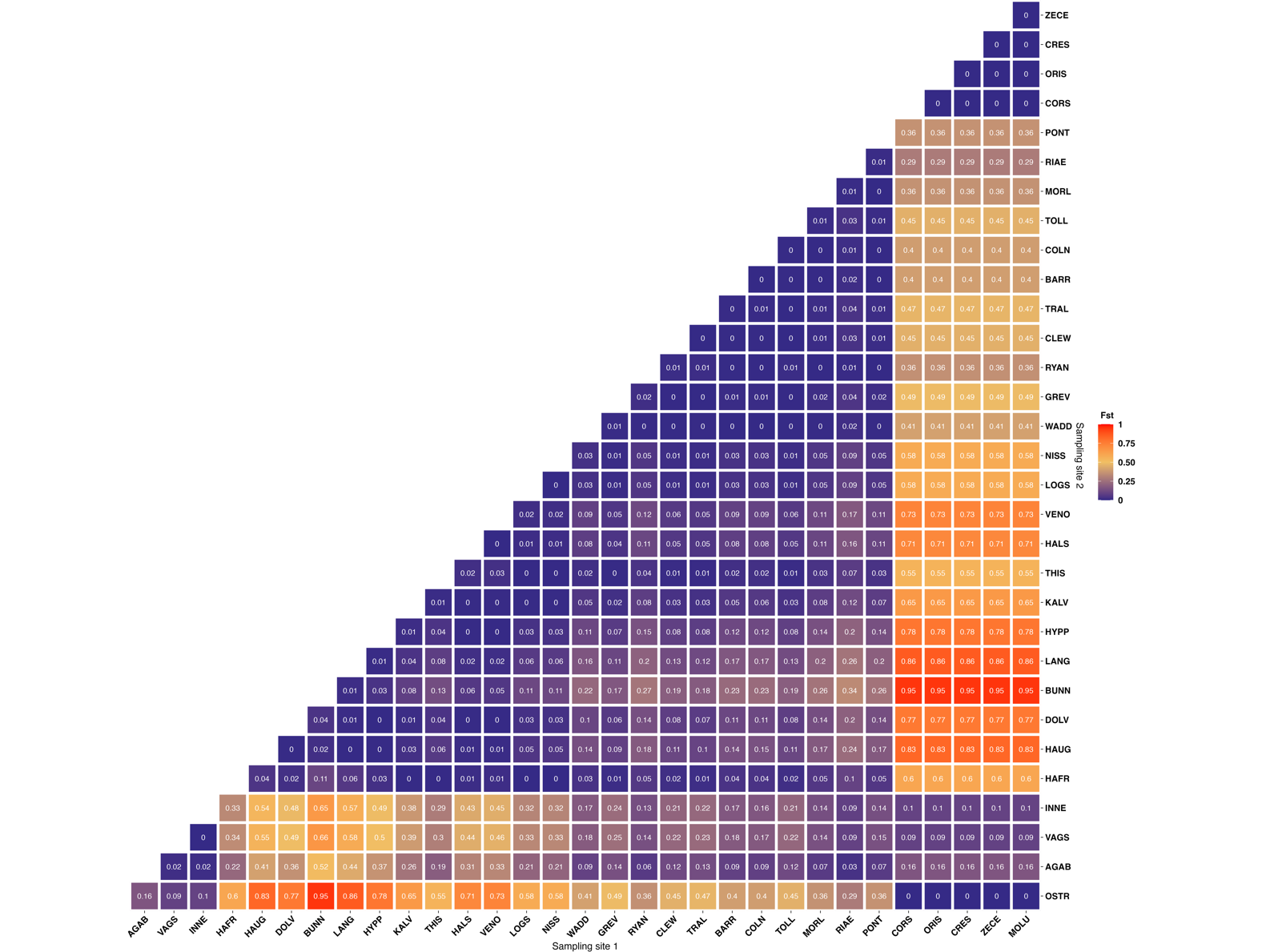


e.


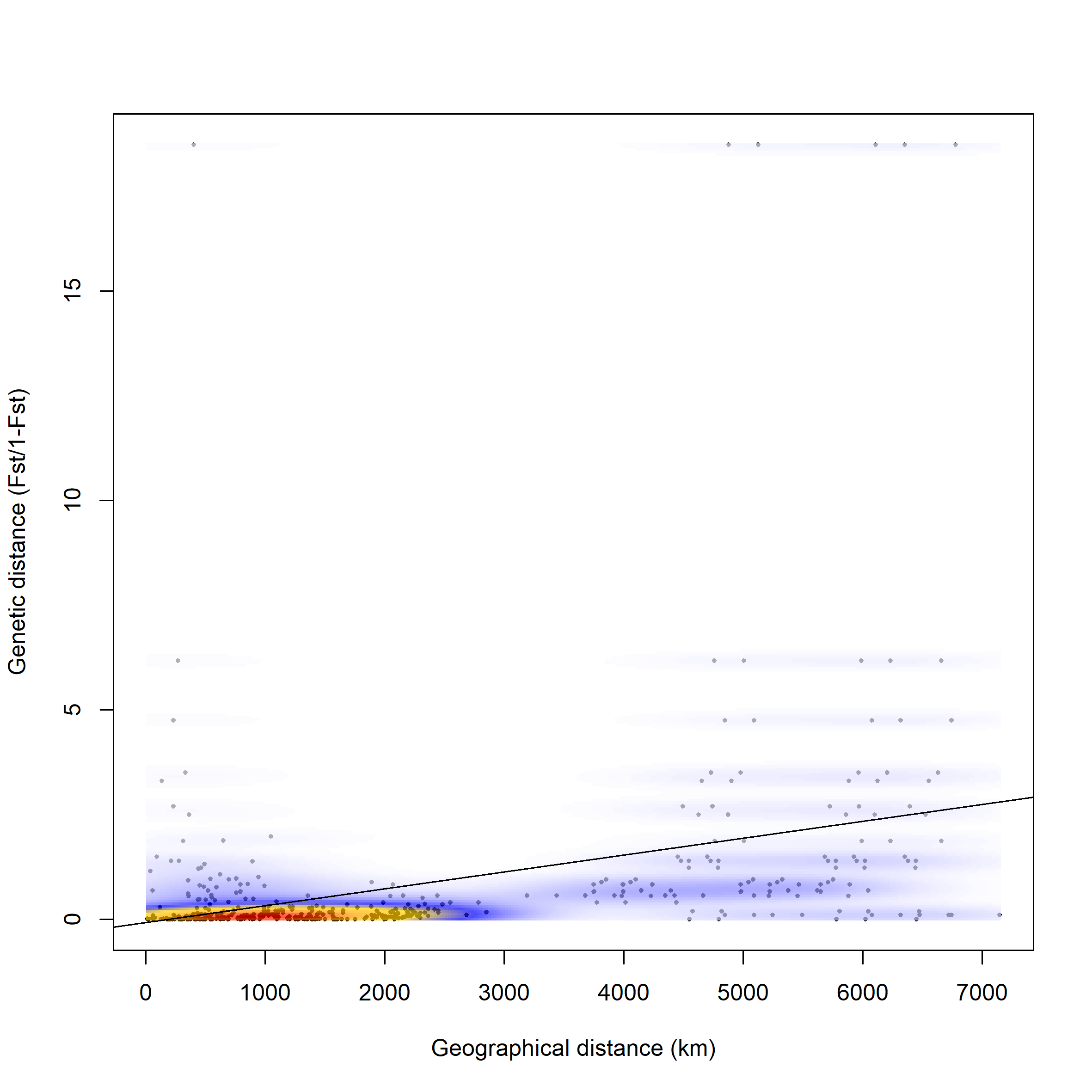


f.


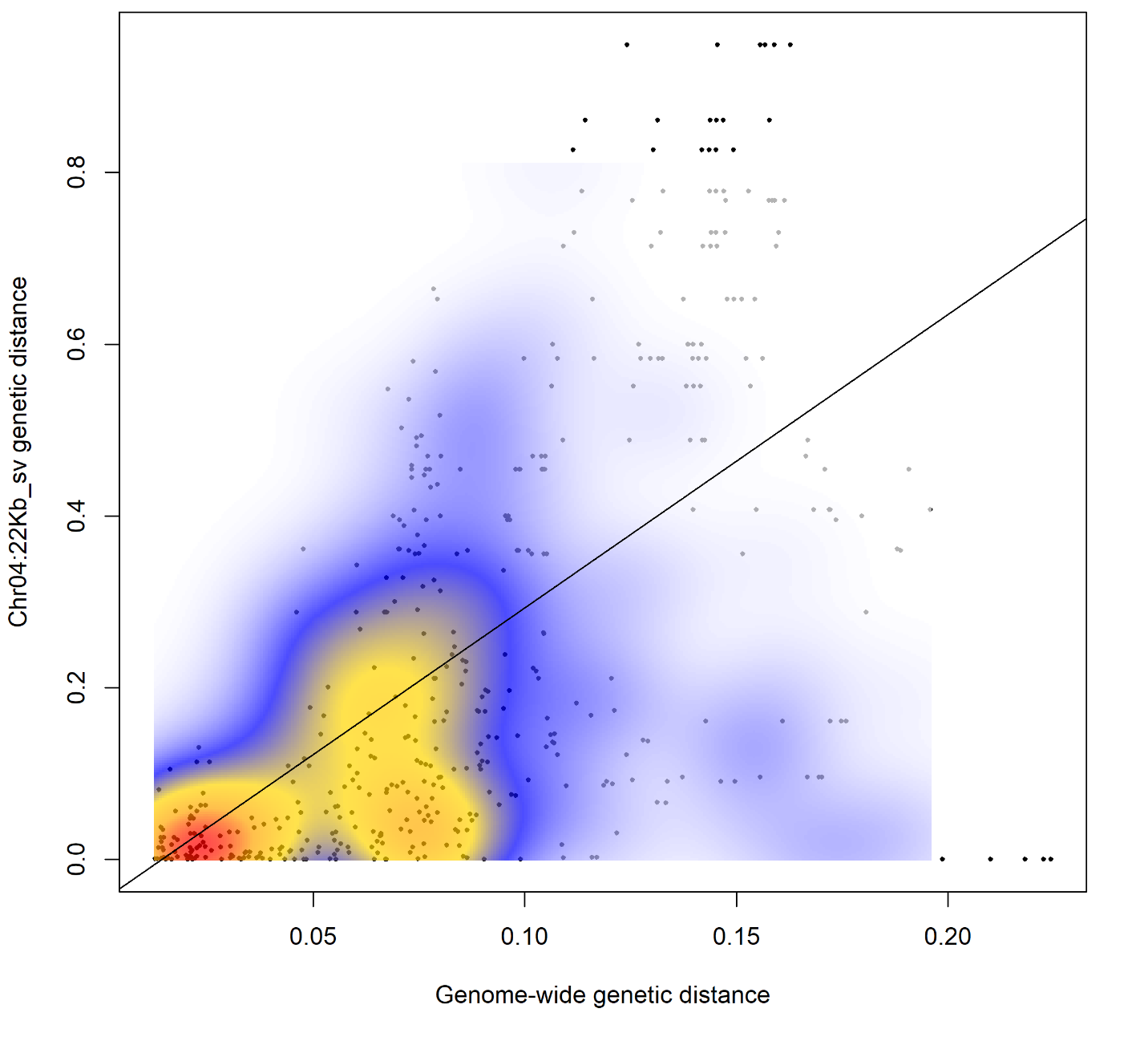


g.


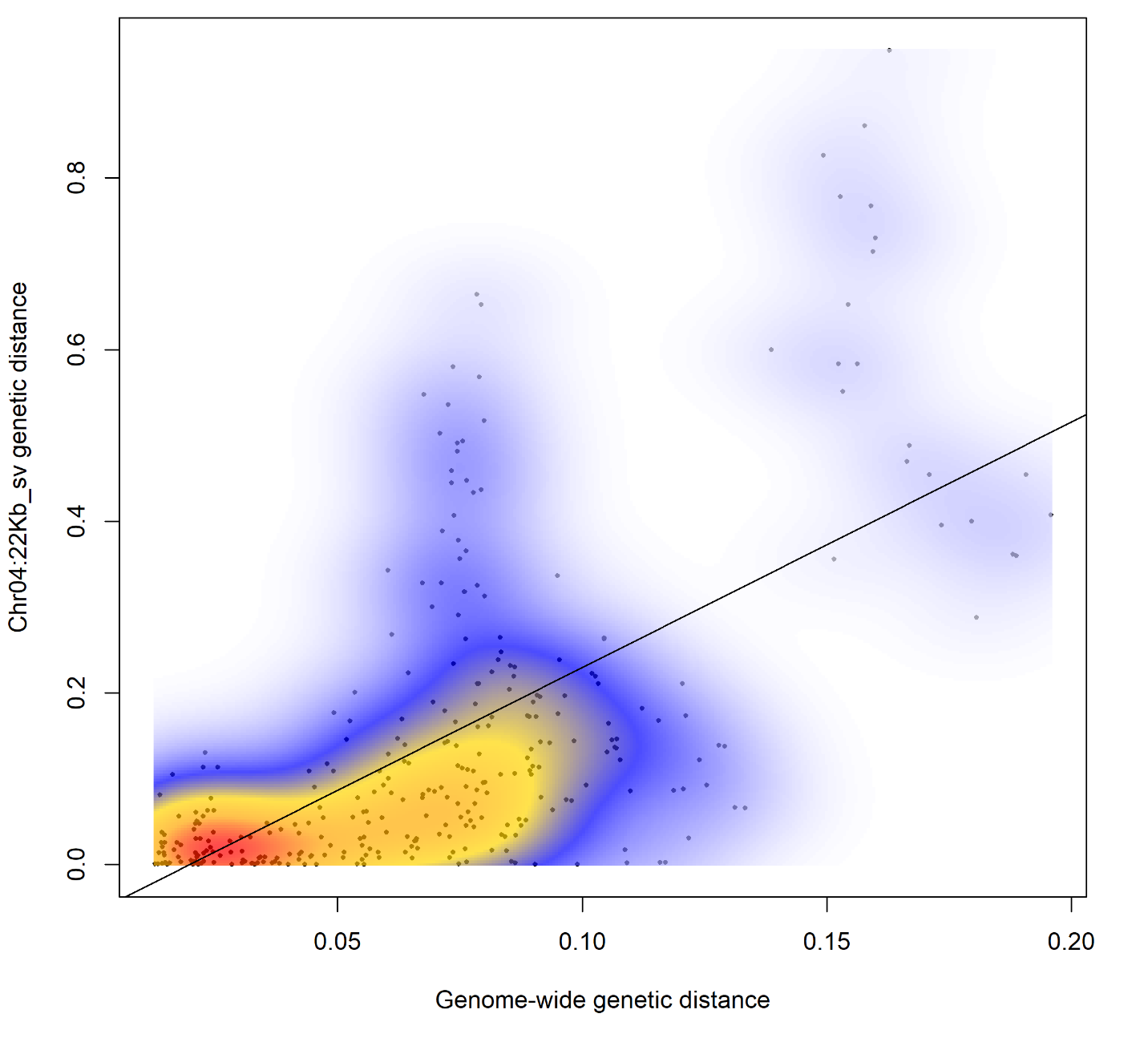


h.


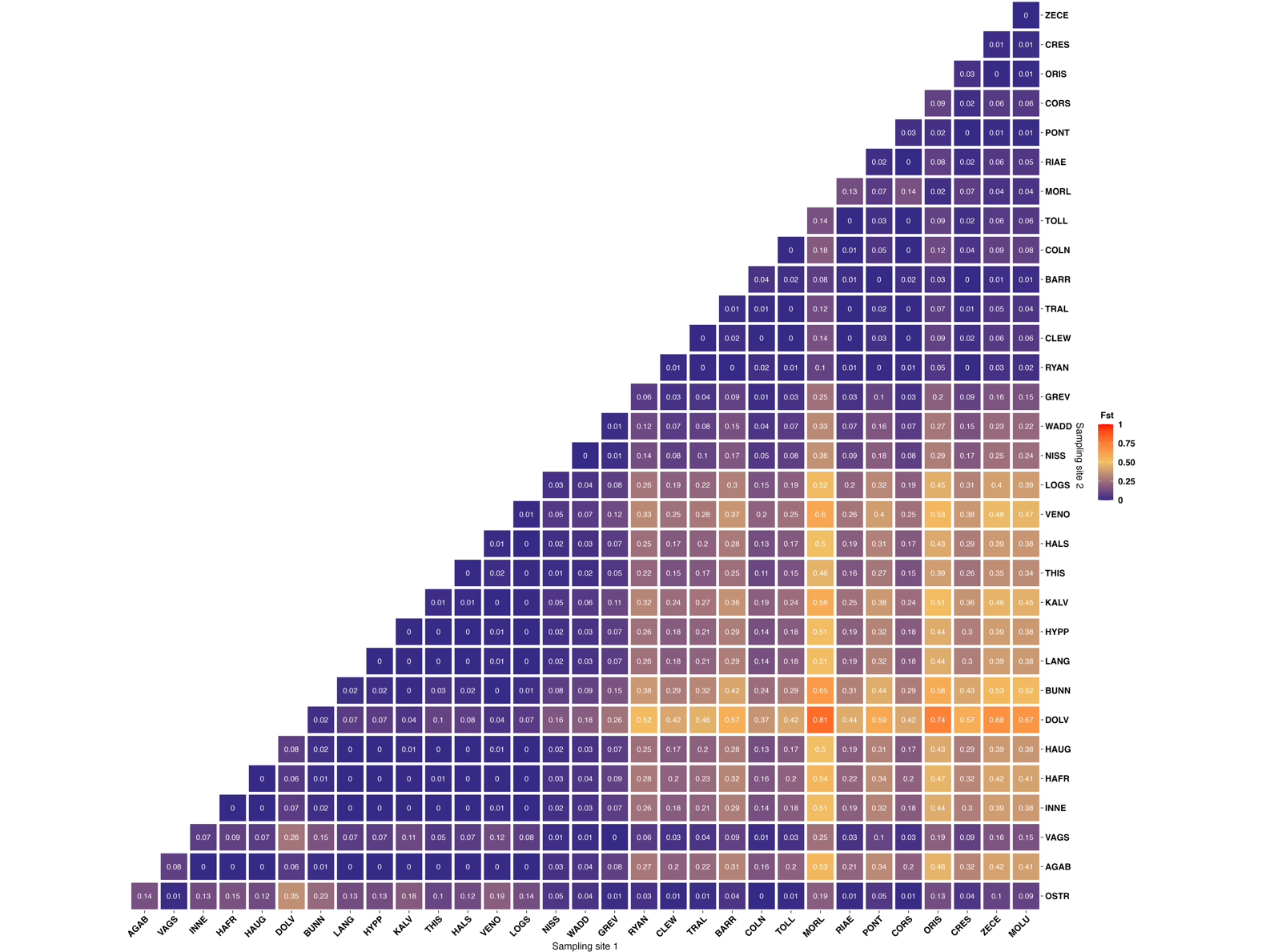


i.


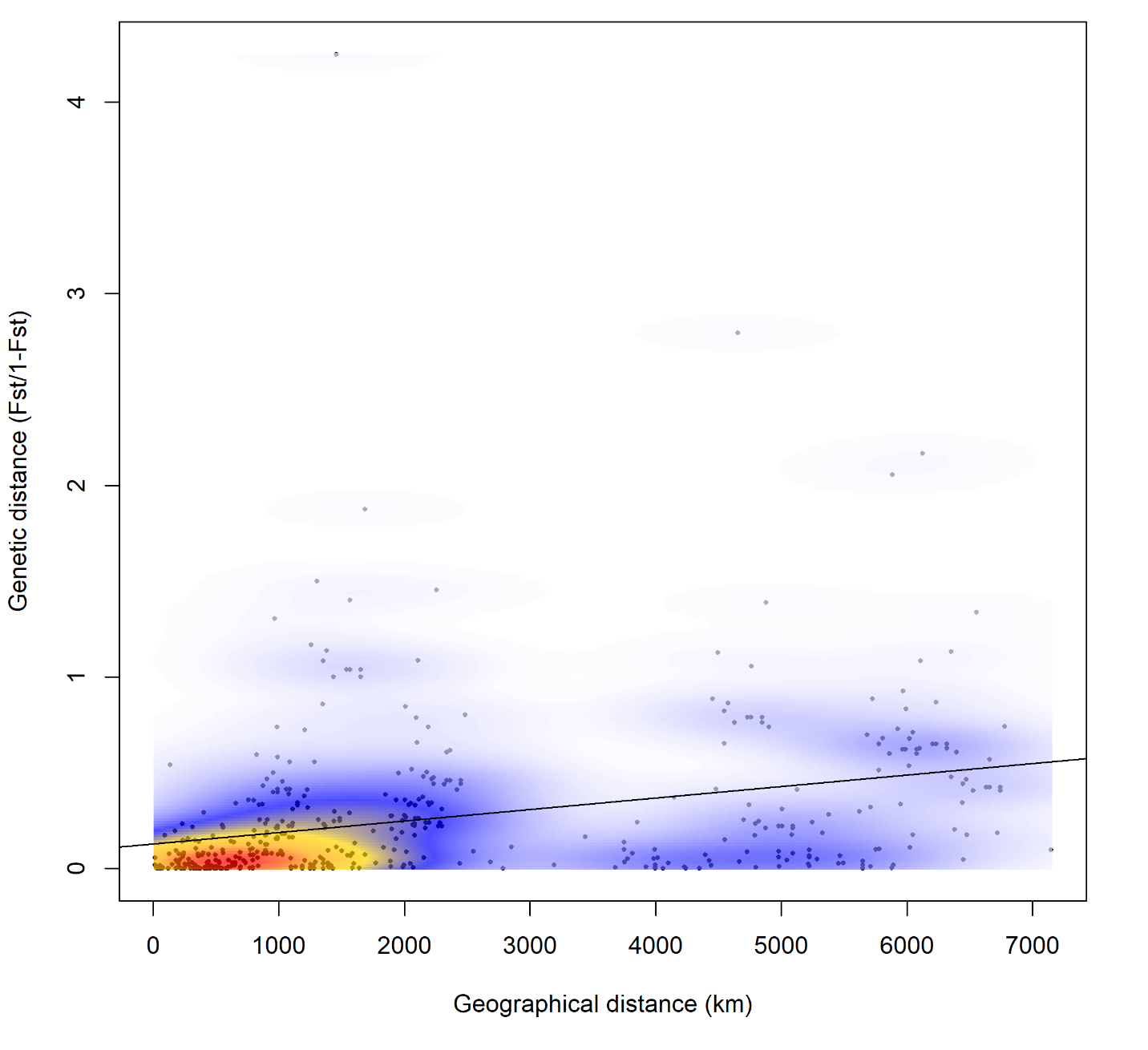


j.


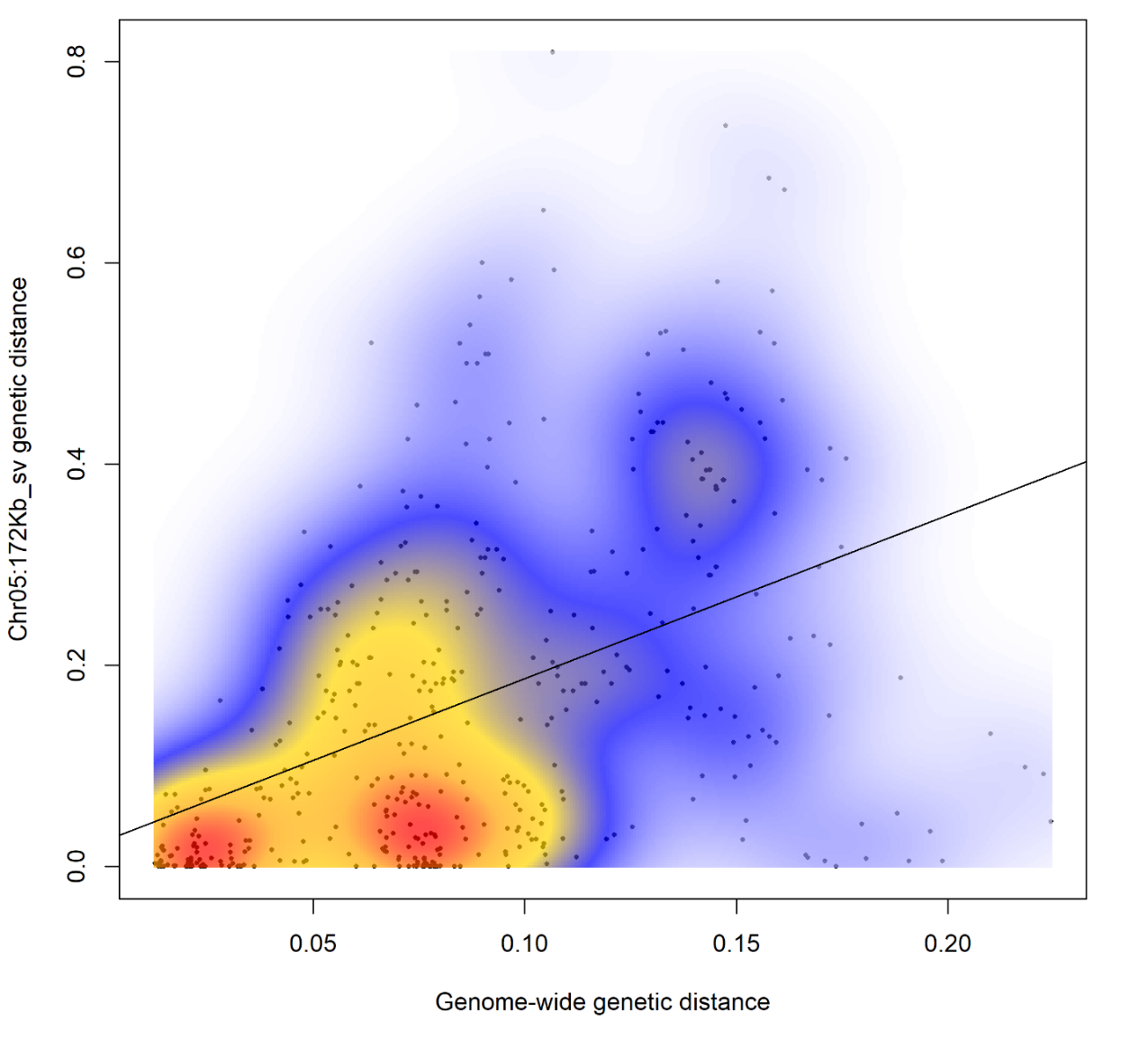


k.


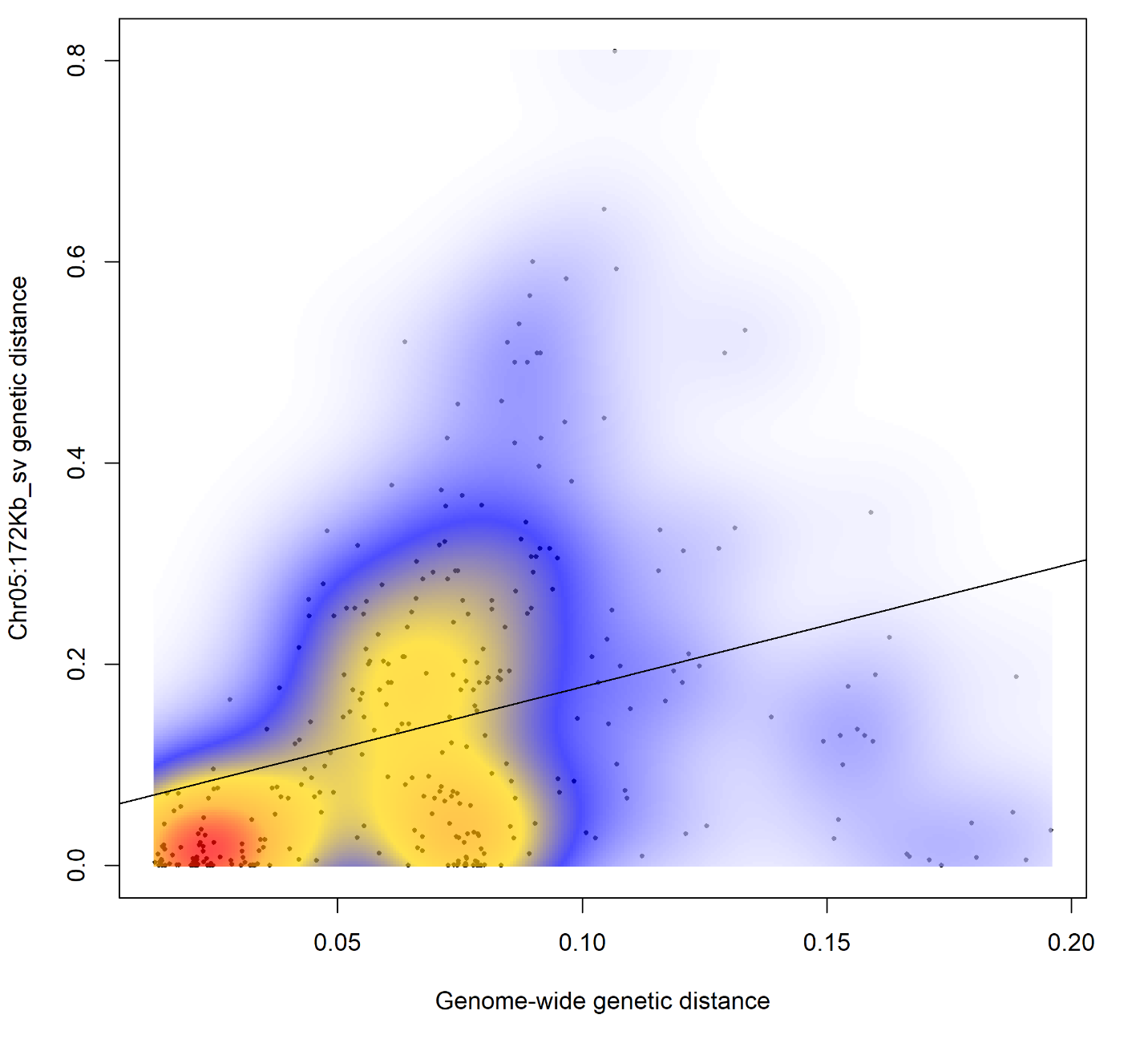


l.


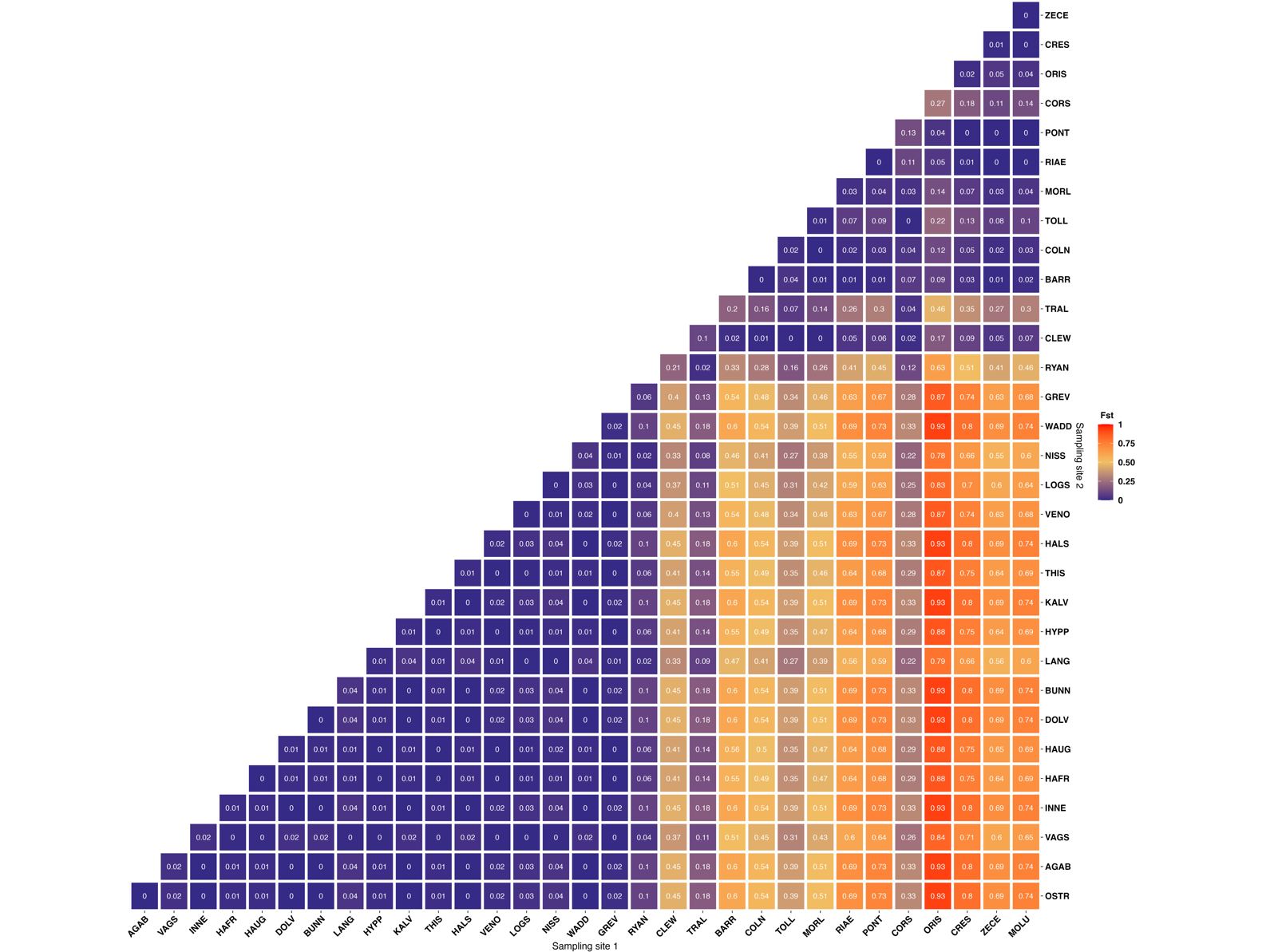


m.


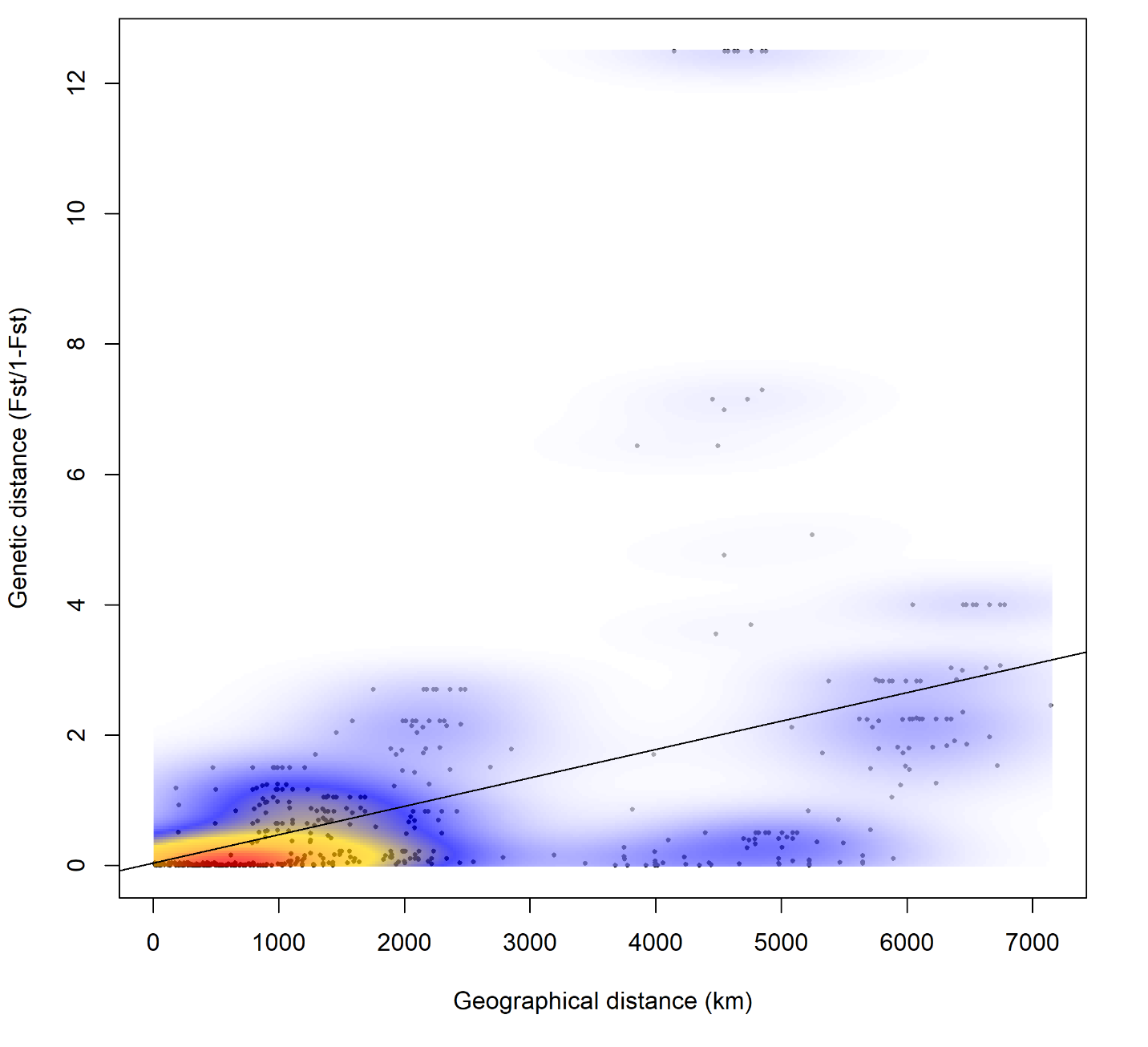


n.


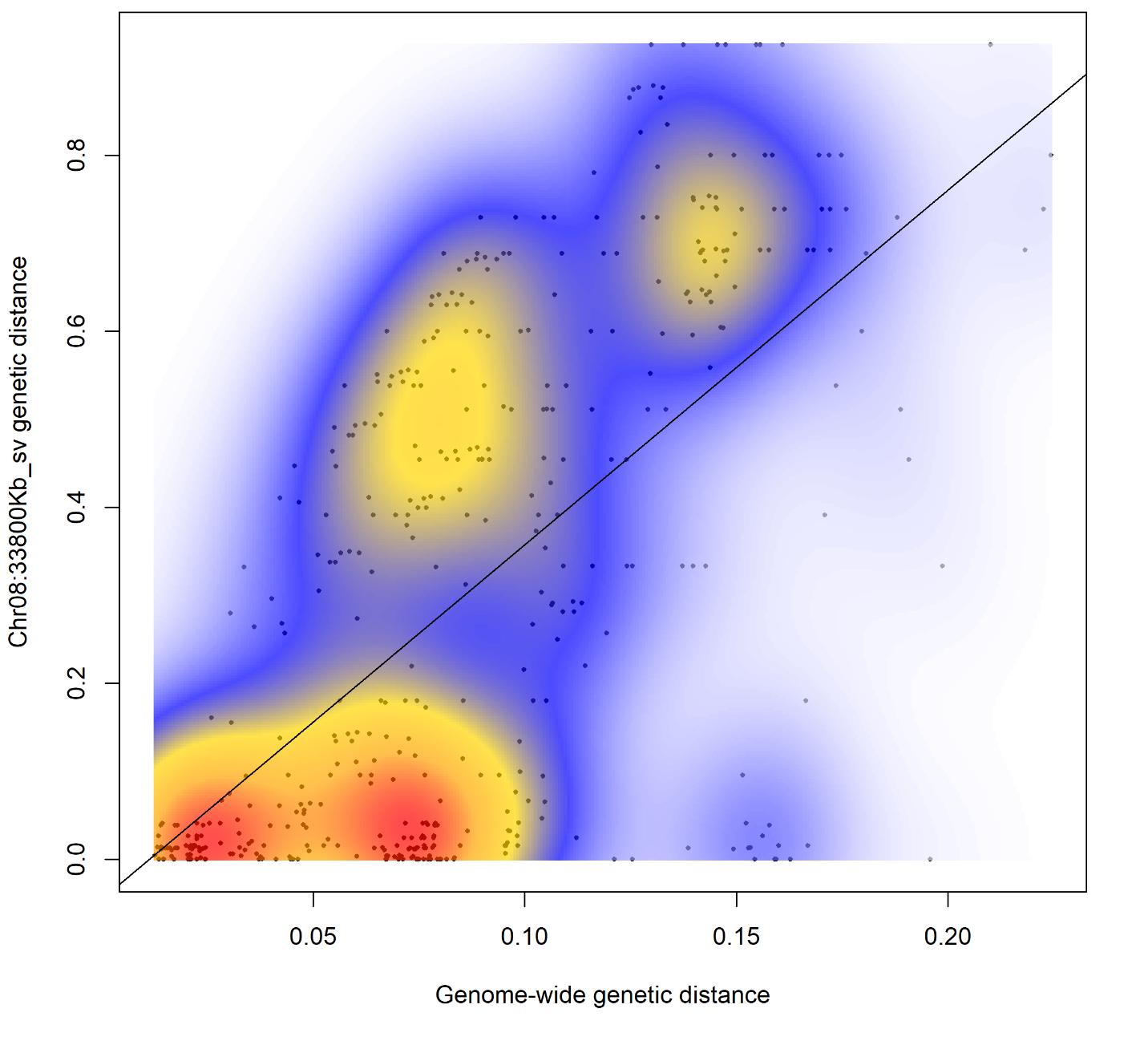


o.


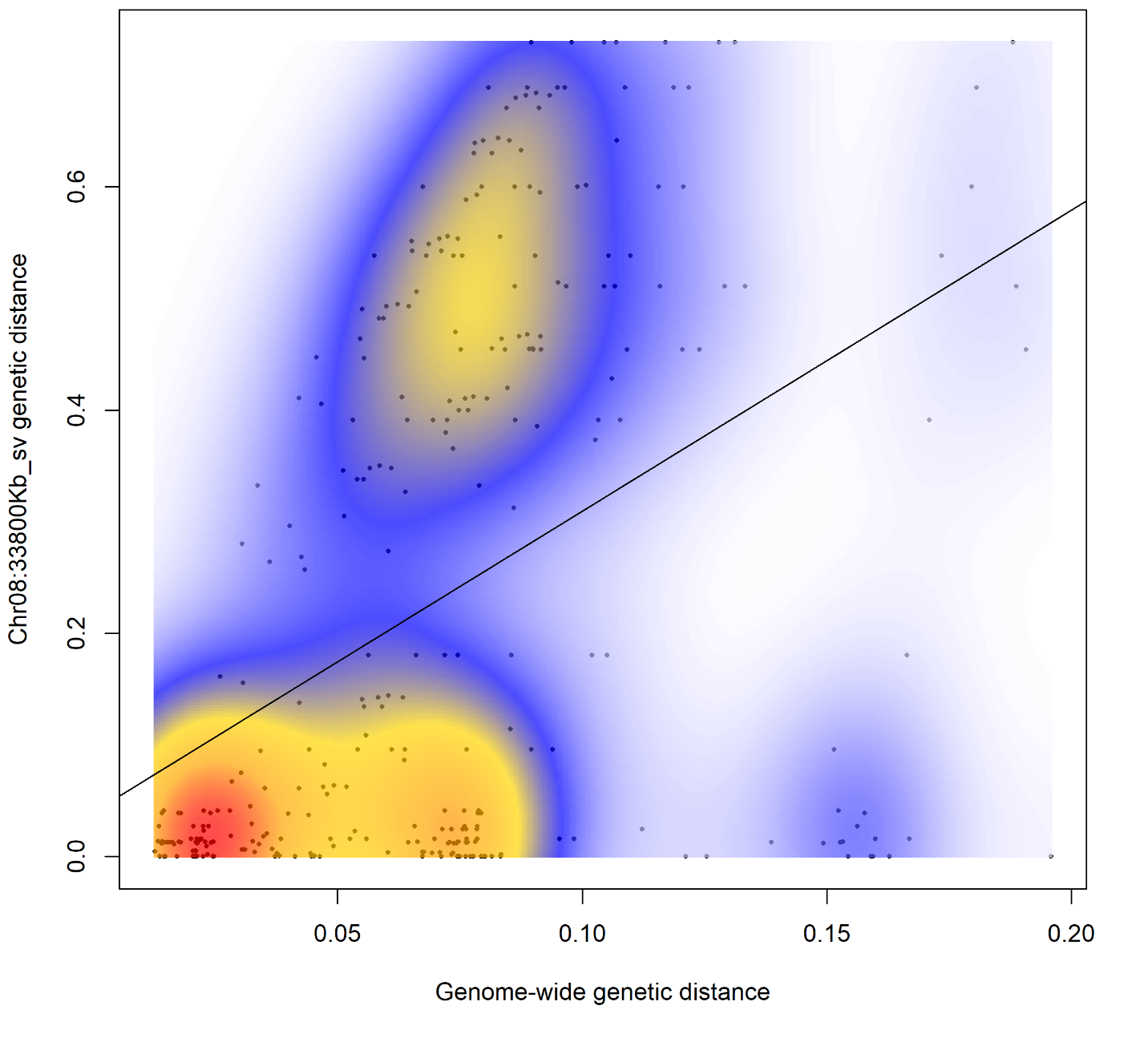


**Fig. S.8 Pairwise FST and relationship between geographic and genetic distances**

a. Genome-wide F_ST_ matrix between all pairs of sampling sites.

b. Pairwise genetic distance (estimated with F_ST_/(1-F_ST_)) in relation to least-waterway geographical distance (in km) for all pairs of population samples (r^2^=0.6415). Mantel randomisation test P<0.0001.

c. Pairwise genetic distance (estimated with F_ST_/(1-F_ST_)) in relation to least-waterway geographical distance (in km) for populations excluding Mediterranean Sea samples (r^2^=0.2895). Mantel randomisation test P=0.0245.

d. Pairwise F_ST_ between all pairs of sampling sites (North American sampling site “USAM” removed) for SV “Chr04:22Kb_sv”

e. Pairwise genetic distance (estimated with F_ST_/(1-F_ST_)) for ‘Chr04:22Kb_sv’ haplotypes in relation to least-waterway geographical distance (in km) for all pairs of population samples (r^2^=0.3580). Mantel randomisation test P<0.0001.

f. Pairwise genetic distance for ‘Chr04:22Kb_sv’ haplotypes (estimated with single-locus F_ST_) in relation to genome-wide genetic distance (estimated with F_ST_ over ~1.4 mil. SNPs) for all pairs of population samples (r^2^=0. 0.6085). Mantel randomisation test P<0.0001.

g. Pairwise genetic distance for ‘Chr04:22Kb_sv’ haplotypes (estimated with single-locus F_ST_) in relation to genome-wide genetic distance (estimated with F_ST_ over ~1.4 mil. SNPs) excluding five Mediterranean population samples (r^2^=0.6250). Mantel randomisation test P<0.0002.

h. Pairwise F_ST_ between all pairs of sampling sites (North American sampling site “USAM” removed) for SV “Chr05:172Kb_sv”,

i. Pairwise genetic distance (estimated with F_ST_/(1-F_ST_)) for ‘Chr05:172Kb_sv’ haplotypes in relation to least-waterway geographical distance (in km) for all pairs of population samples (r^2^=0.3077). Mantel randomisation test P<0.0004

j. Pairwise genetic distance for ‘Chr05:172Kb_sv’ haplotypes (estimated with single-locus F_ST_) in relation to genome-wide genetic distance (estimated with F_ST_ over ~1.4 mil. SNPs) for all pairs of population samples (r^2^=0.4511). Mantel randomisation test P<0.0001.

k. Pairwise genetic distance for ‘Chr05:172Kb_sv’ haplotypes (estimated with single-locus F_ST_) in relation to genome-wide genetic distance (estimated with F_ST_ over ~1.4 mil. SNPs) excluding five Mediterranean population samples (r^2^=0.3226). Mantel randomisation test P=0.0105.

l. Pairwise F_ST_ between all pairs of sampling sites (North American sampling site “USAM” removed) for SV “Chr08:33800Kb_sv”

m. Pairwise genetic distance (estimated with F_ST_/(1-F_ST_)) for ‘Chr08:33800Kb_sv’ haplotypes in relation to least-waterway geographical distance (in km) for all pairs of population samples (r^2^=0.4570). Mantel randomisation test P<0.0001

n. Pairwise genetic distance for ‘Chr08:33800Kb_sv’ haplotypes (estimated with single-locus F_ST_) in relation to genome-wide genetic distance (estimated with F_ST_ over ~1.4 mil. SNPs) for all pairs of population samples (r^2^=0.6287). Mantel randomisation test P<0.0001.

o. Pairwise genetic distance for ‘Chr08:33800Kb_sv’ haplotypes (estimated with single-locus F_ST_) in relation to genome-wide genetic distance (estimated with F_ST_ over ~1.4 mil. SNPs) excluding five Mediterranean population samples (r^2^=0.4243). Mantel randomisation test P=0.0011.

**Fig. S9. Estimate of relatedness per sampling site.**

*Rationale*: This figure illustrates relatedness values for pairs of individuals within the 33 sites. Different shades of grey represent the proportion of all pairwise comparisons within a single population corresponding to a specific relatedness class. Black bars represent the proportion of unrelated individuals within a population

a.


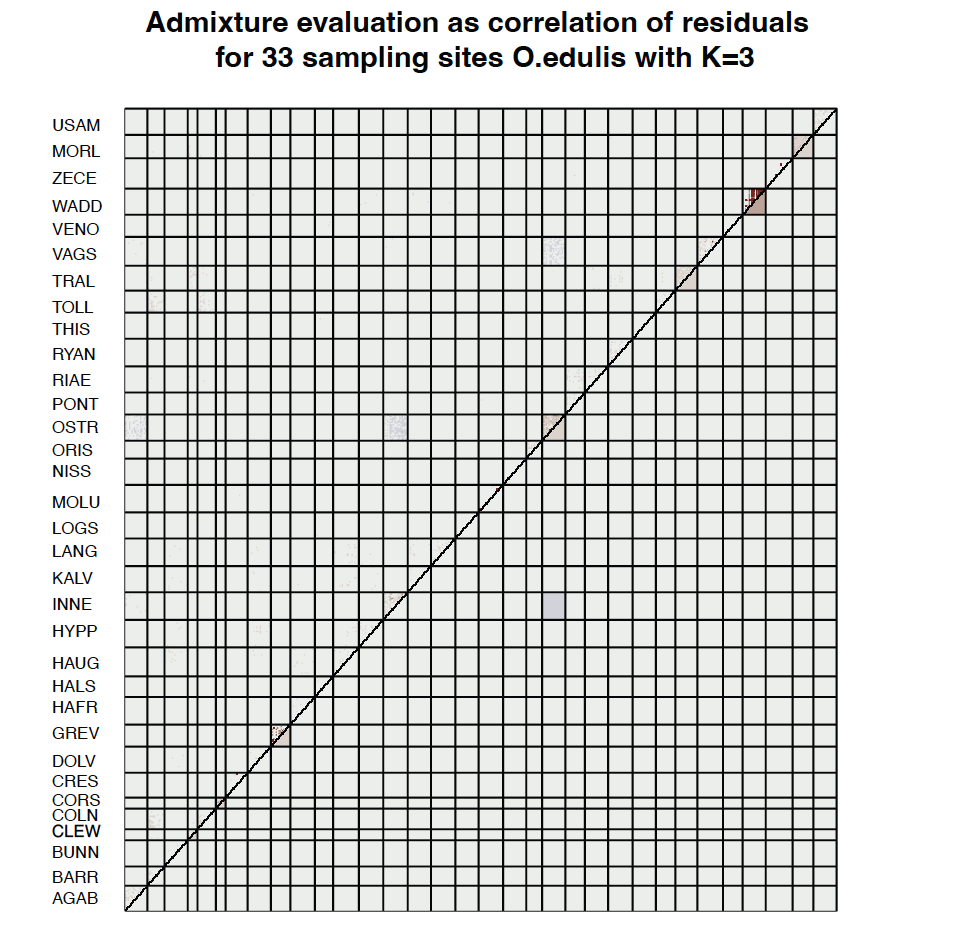


b.


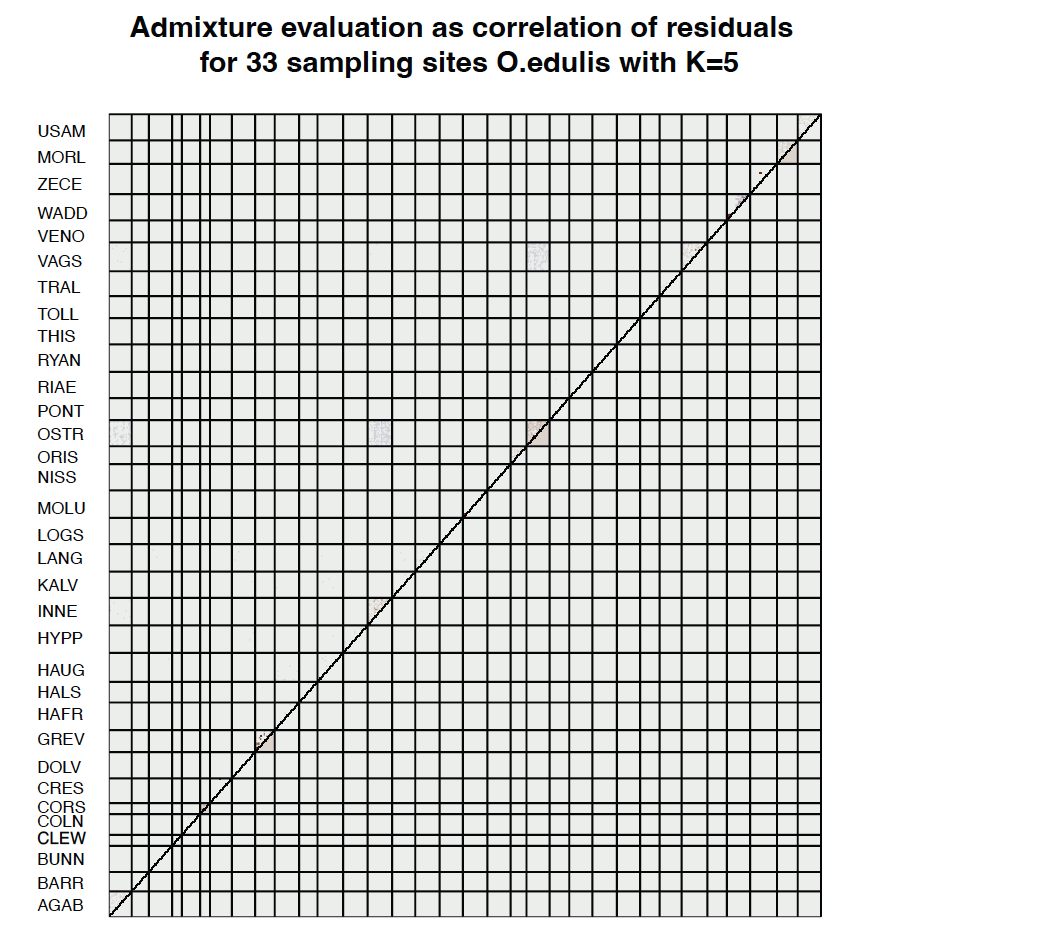


c.


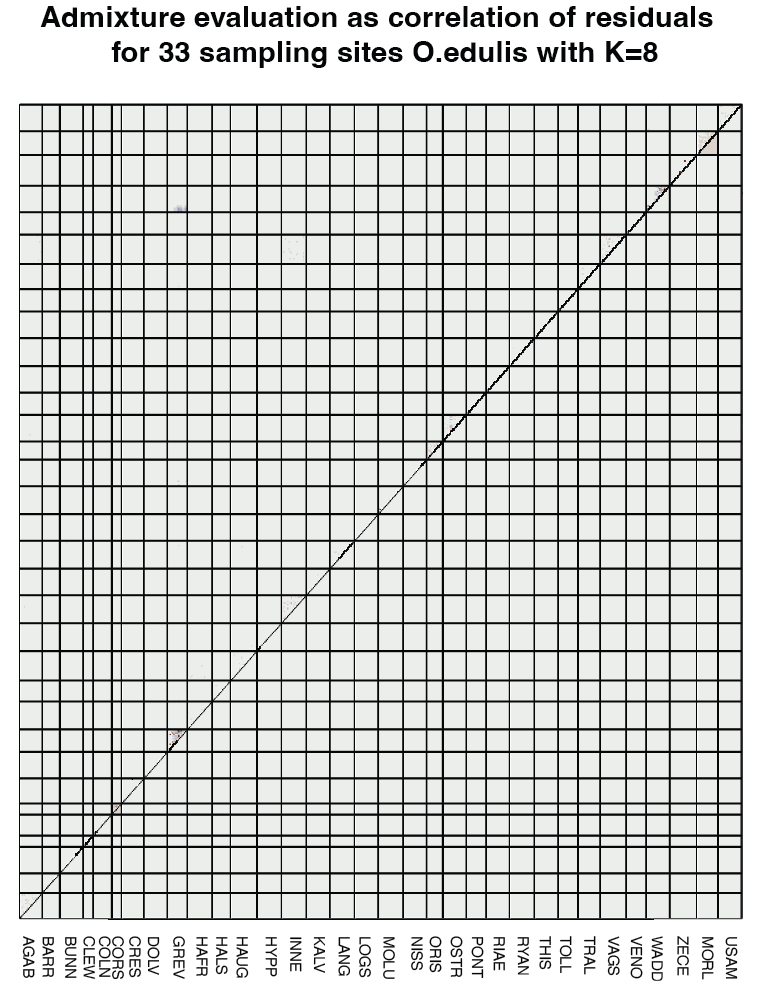


d.

**Fig. S.10 Correlation of residuals from admixture analysis with K=10.**

*Rationale*: Evaluation of NGSadmix results assuming K = 10 as the correlation of residuals obtained with evalAdmix. EvalAdmix is a tool designed to assess the results of an admixture analysis. It produces a pairwise correlation of residuals matrix, where correlations close to 0 indicate an adequate fit to the admixture model, positive correlations may suggest similar demographic histories or relatedness, and negative correlations may signify different histories but shared ancestral populations.

a. K=3

b. K=5

c. K=8

d. K=10


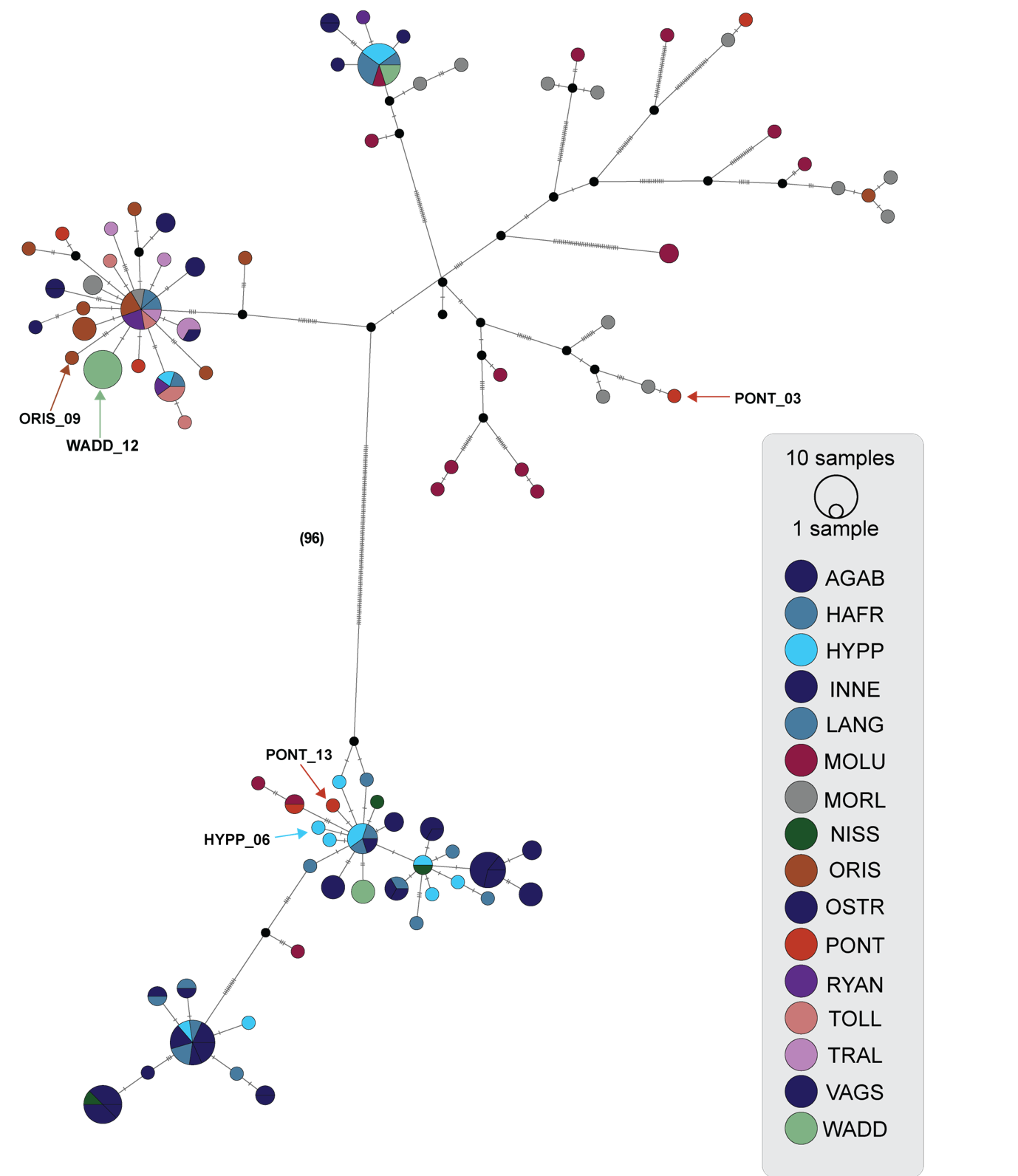


**Fig. S.11 Mitogenome haplotype network.**

*Rationale*: In addition to the analysis of variation across nuclear SNPs, we also examined patterns of sequence variation across the mitochondrial genome. Individuals included in the PSMC analysis are indicated by an arrow and a sample tag.

a.

b.

c.

**Fig. S.12 Genome-wide heterozygosity and genetic diversity estimates.**

a. Observed fraction of heterozygous sites (*H_o_*) per individuals for the 33 sampling sites.

b. Nucleotide diversity (*π*), Watterson's θ (*θw*), and Tajima's D.

c. Pearson correlation between average mean depth and diversity estimates across sampling sites. The number of sites, nucleotide diversity, and Watterson’s Theta show positive correlations with average mean depth, while Tajima’s D does not. Sampling sites with extreme values of nucleotide diversity are highlighted.

1. b. c.


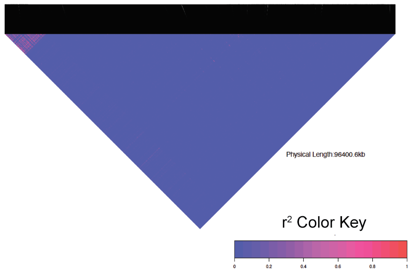

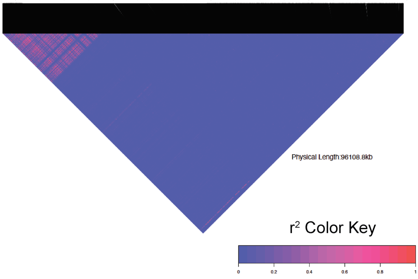

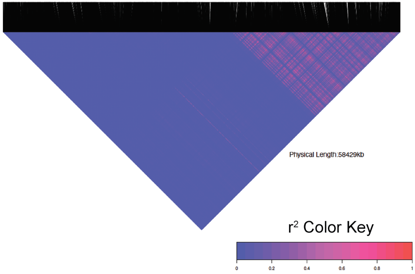


**Fig. S.13 Linkage disequilibrium at pseudo-chromosomes 4, 5 and 8.**

*Rationale*: from the global SNPs dataset (Dataset I), we selected one SNP from every 100 SNPs in the Beagle file to reduce the computational time of ngsLD (Fox et al., 2019). LD was estimated for each pseudo-chromosome using this subset of SNPs.

a. Pairwise SNP LD heatmap for pseudo-chromosome 4

b. Pairwise SNP LD heatmap for pseudo-chromosome 5

c. Pairwise SNP LD heatmap for pseudo-chromosome 8

a. **
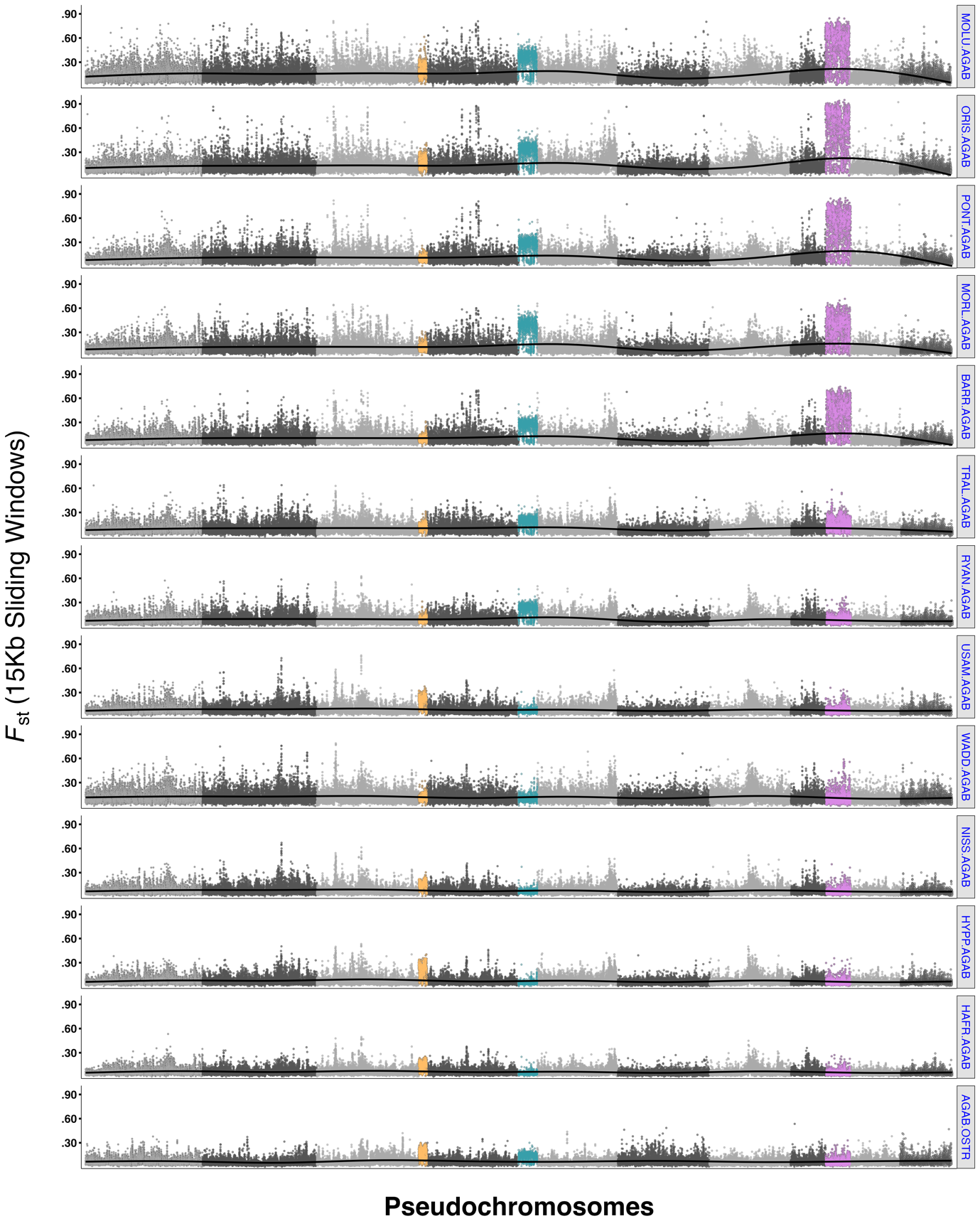
**

b.
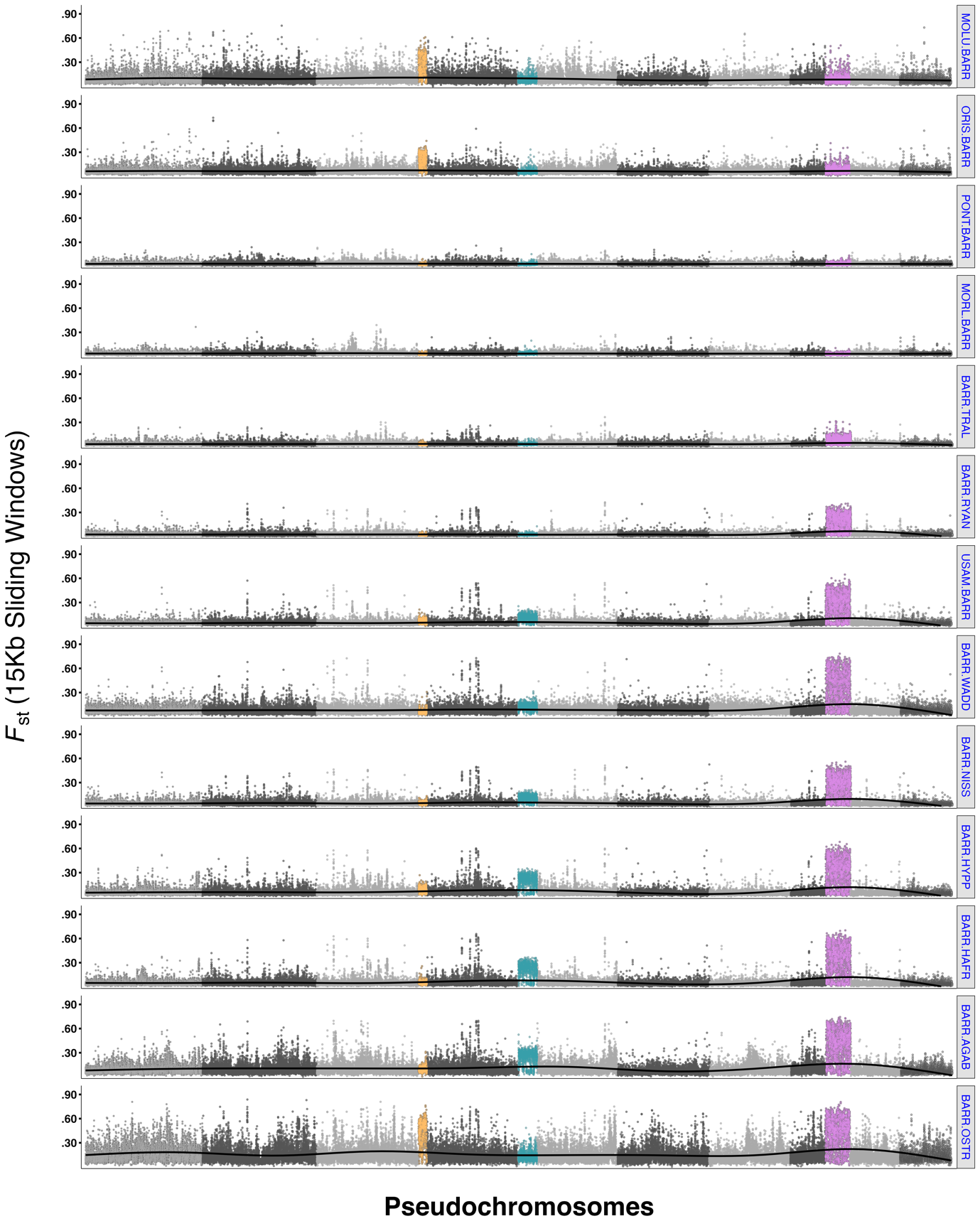


c.
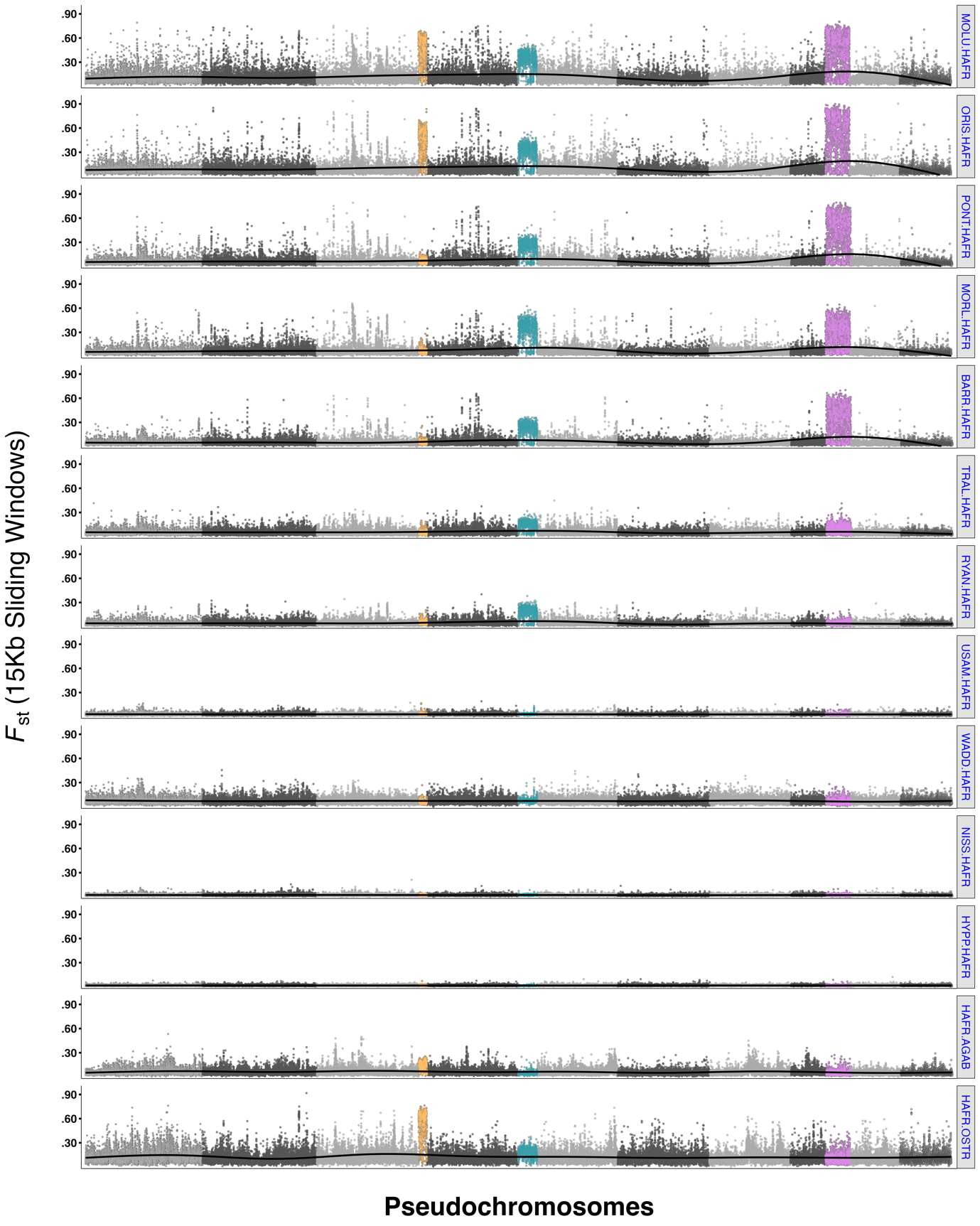


d.
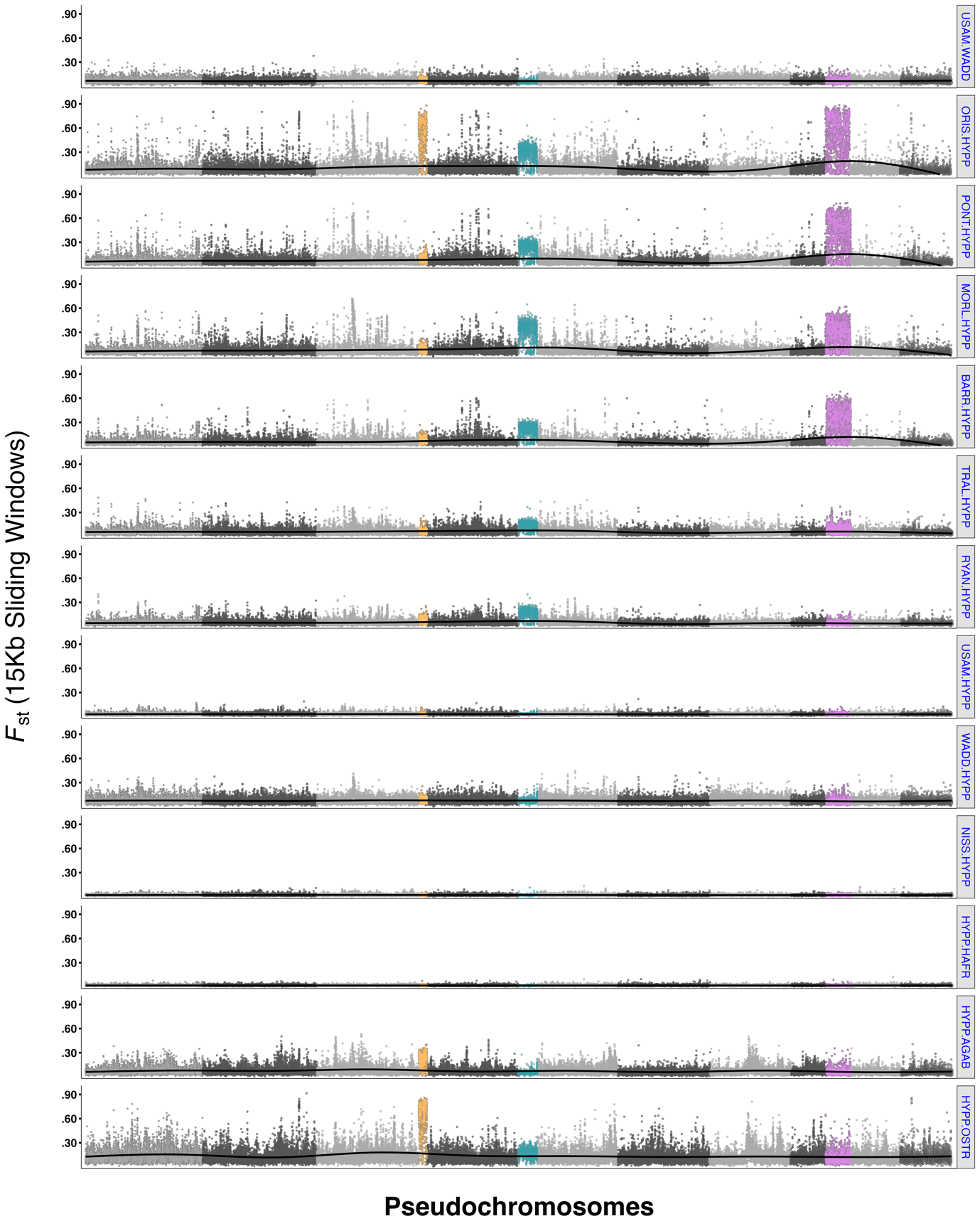


e.
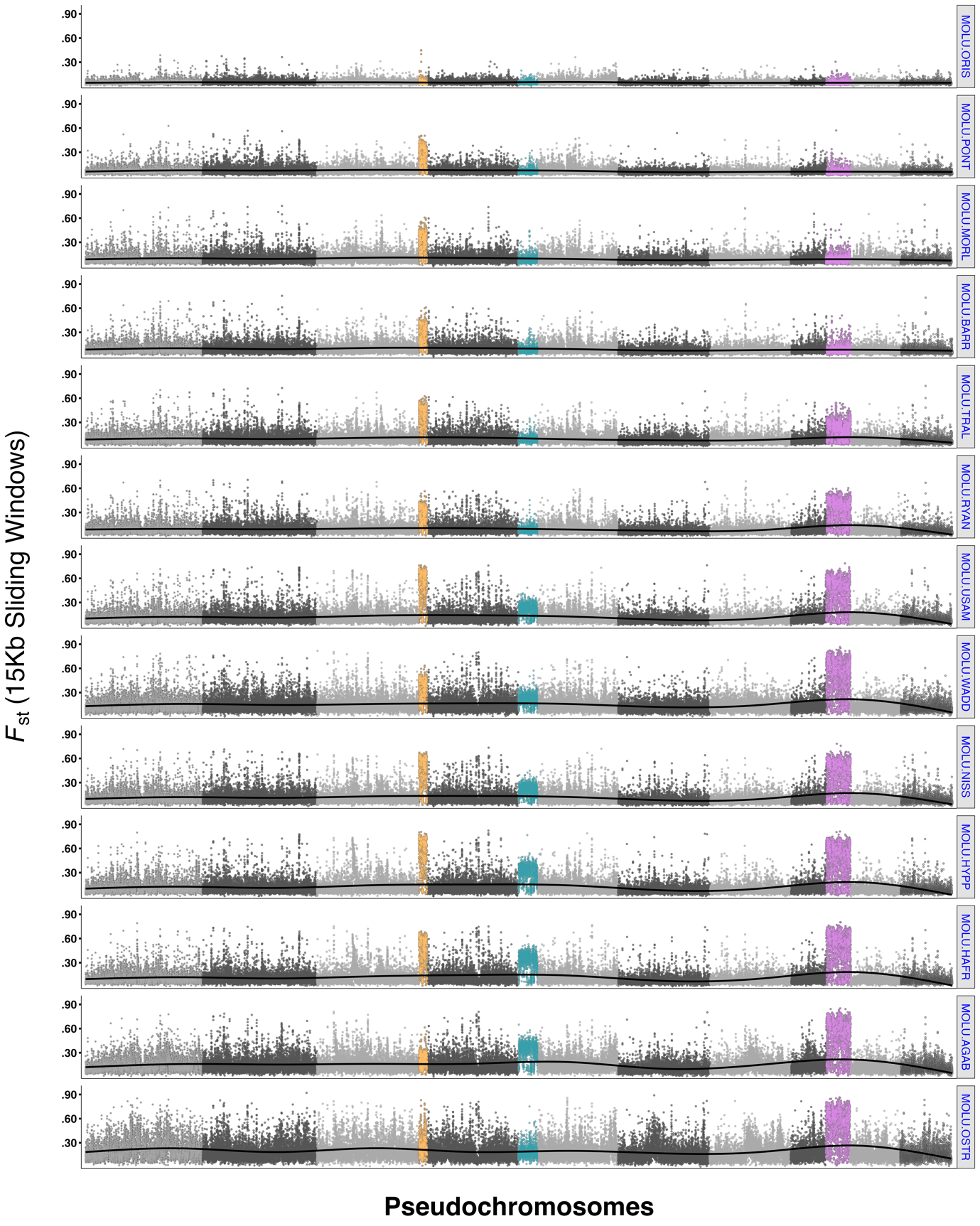


f.
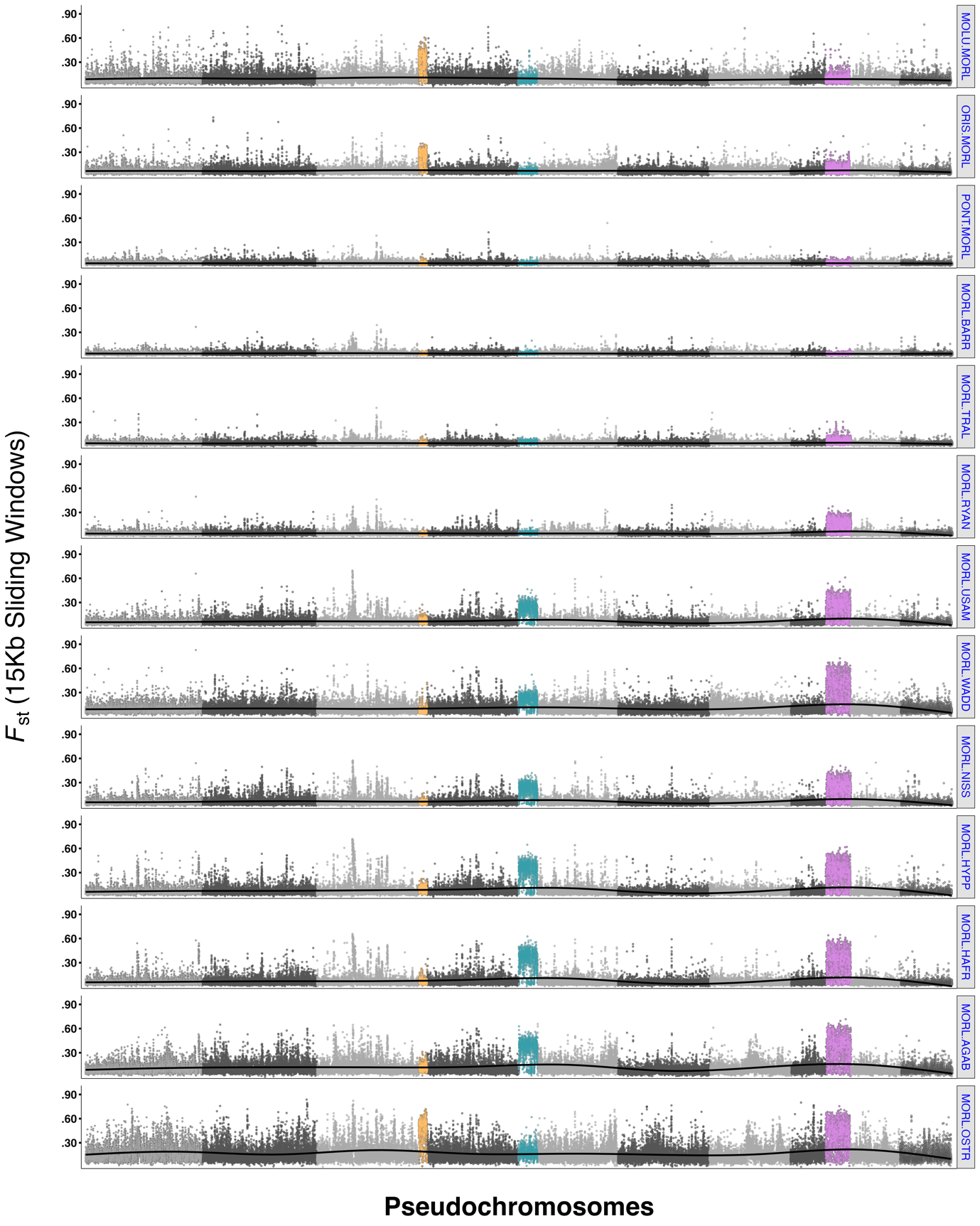


g.
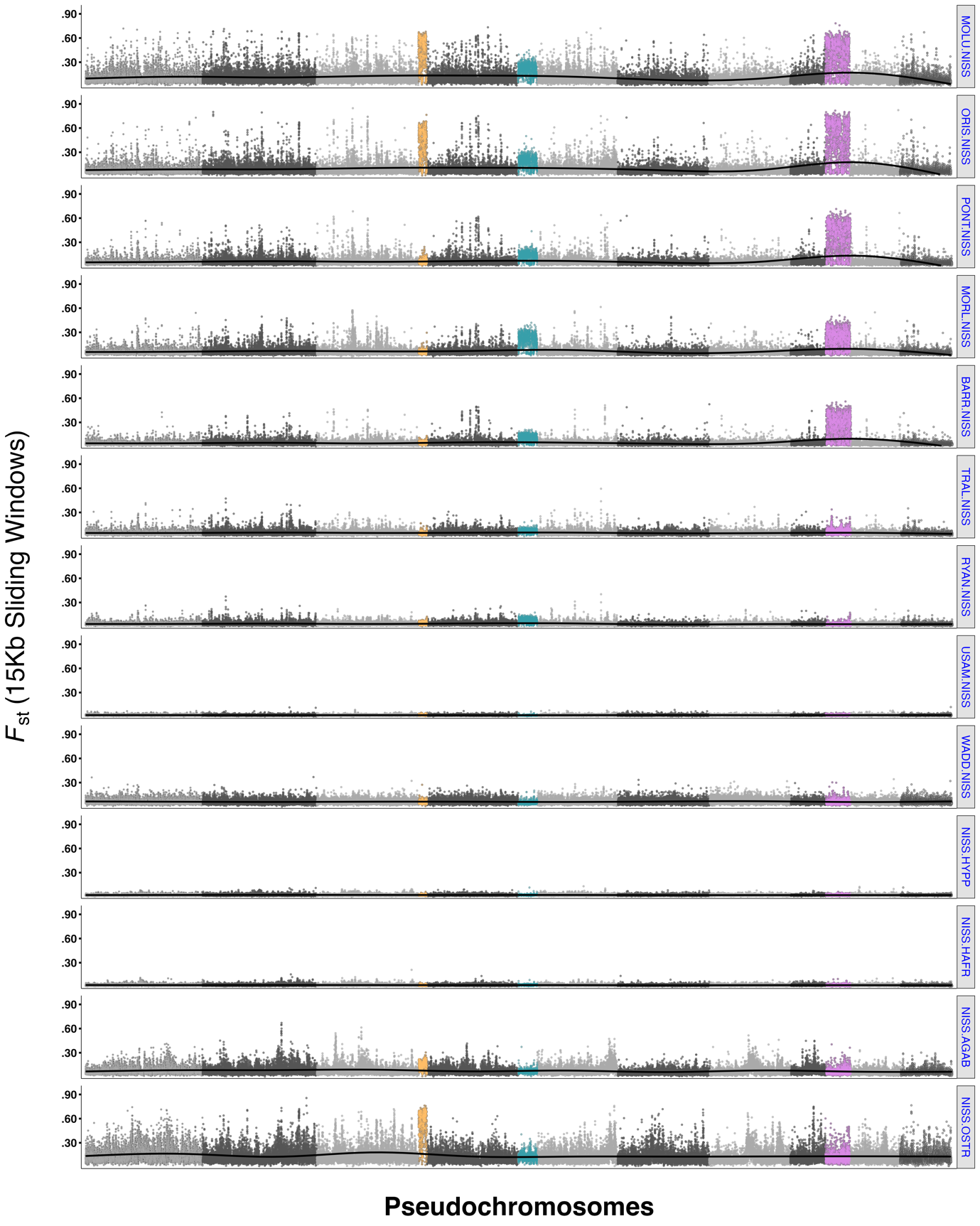


h.
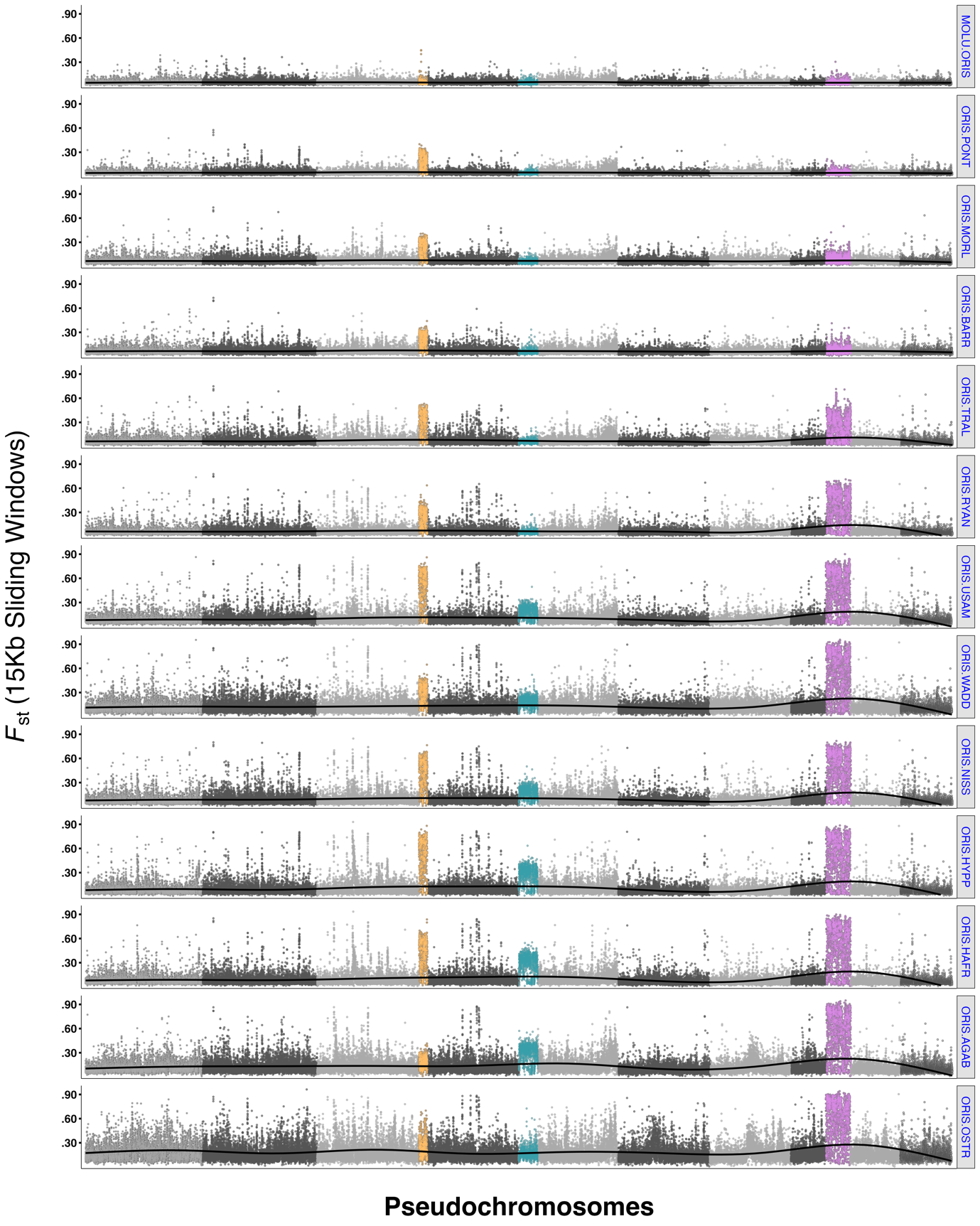


i.
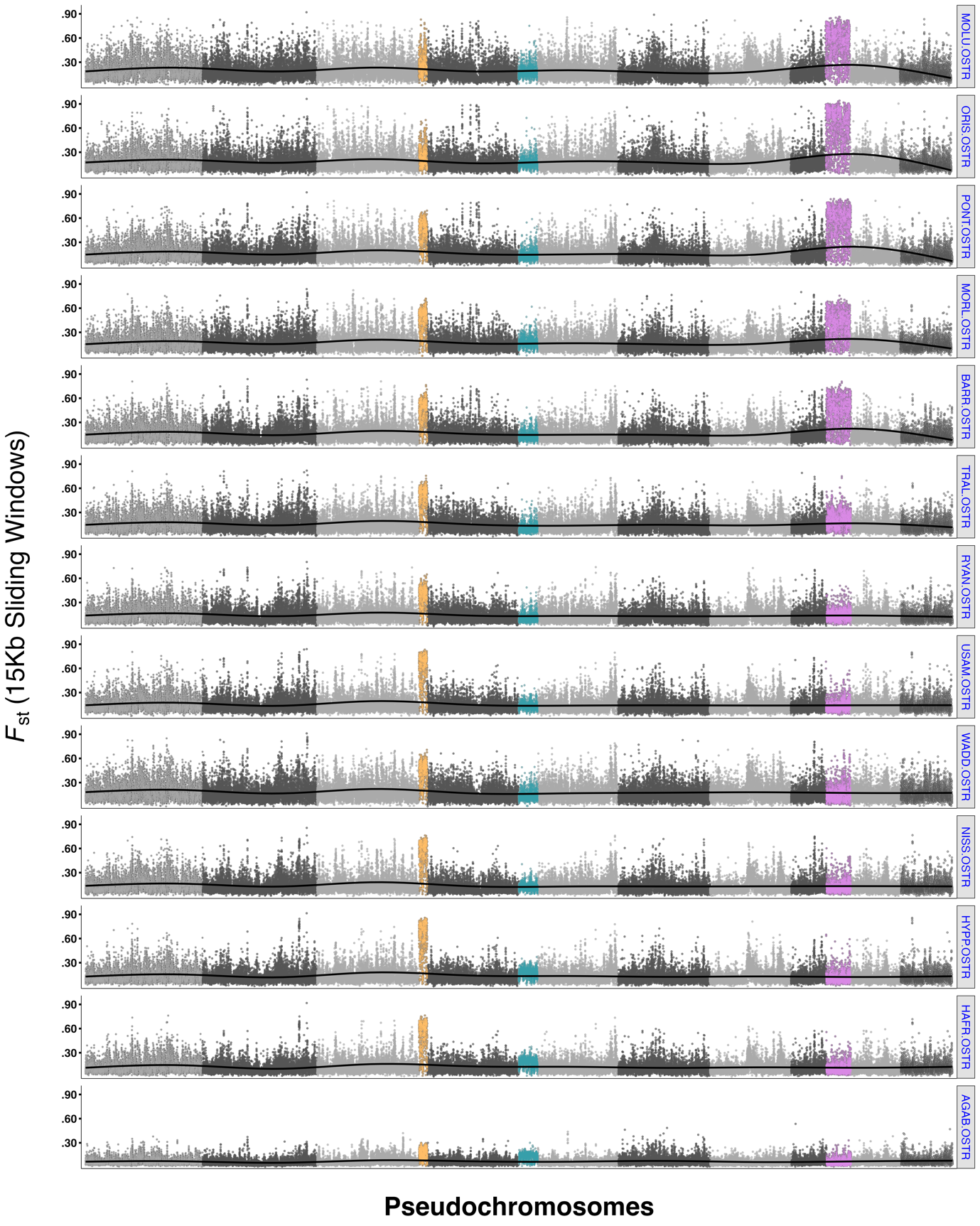


j.
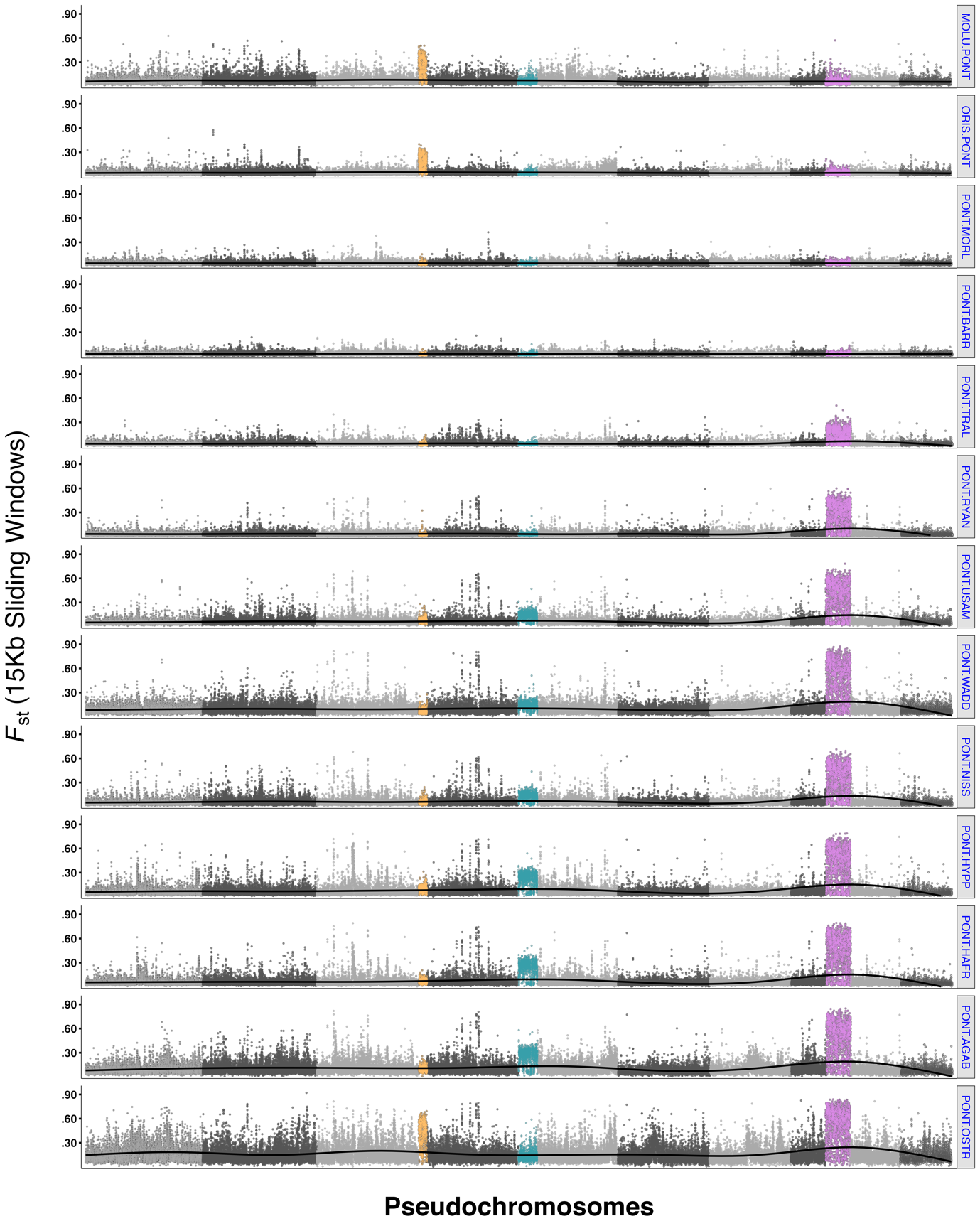


k.
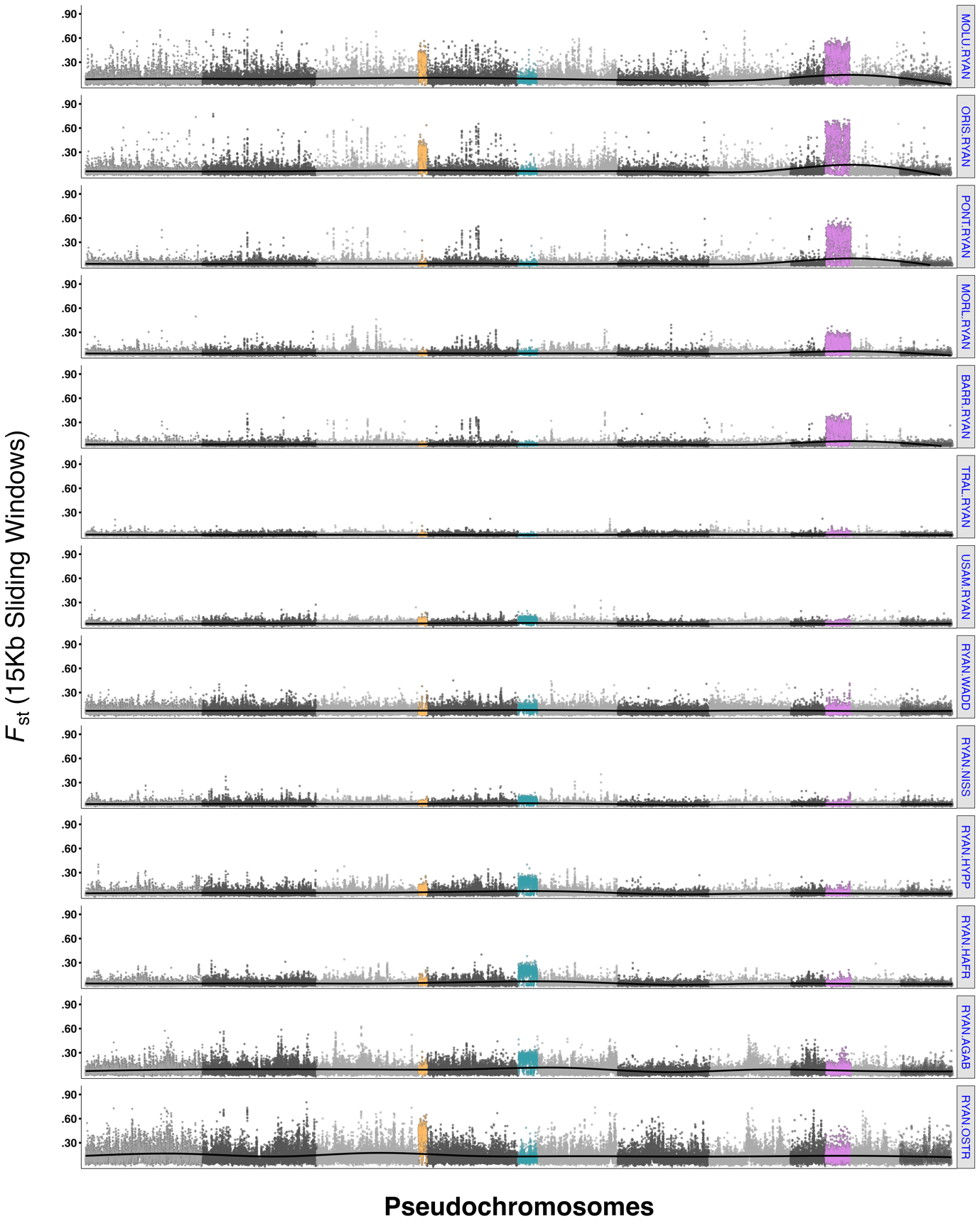


l.
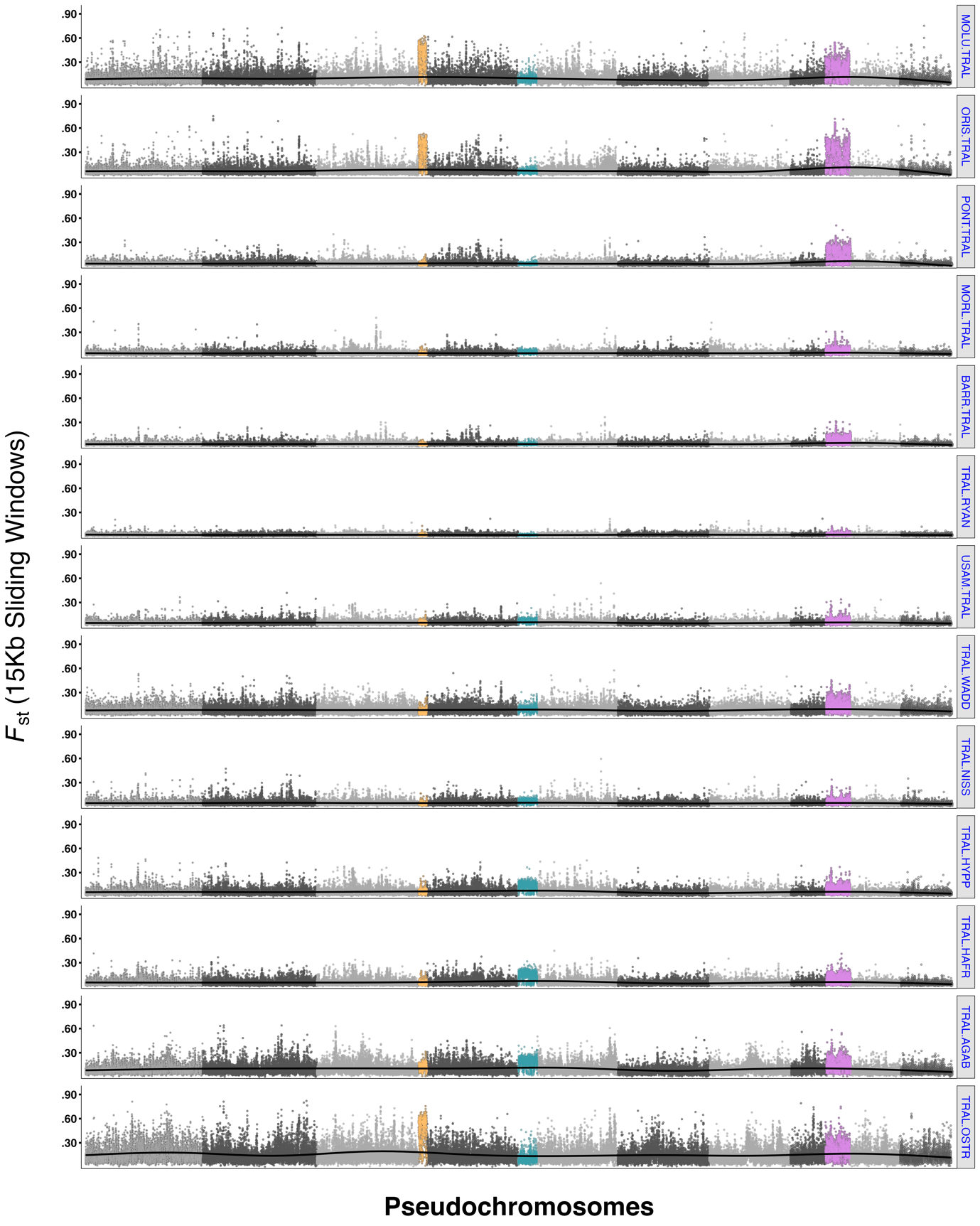


m.
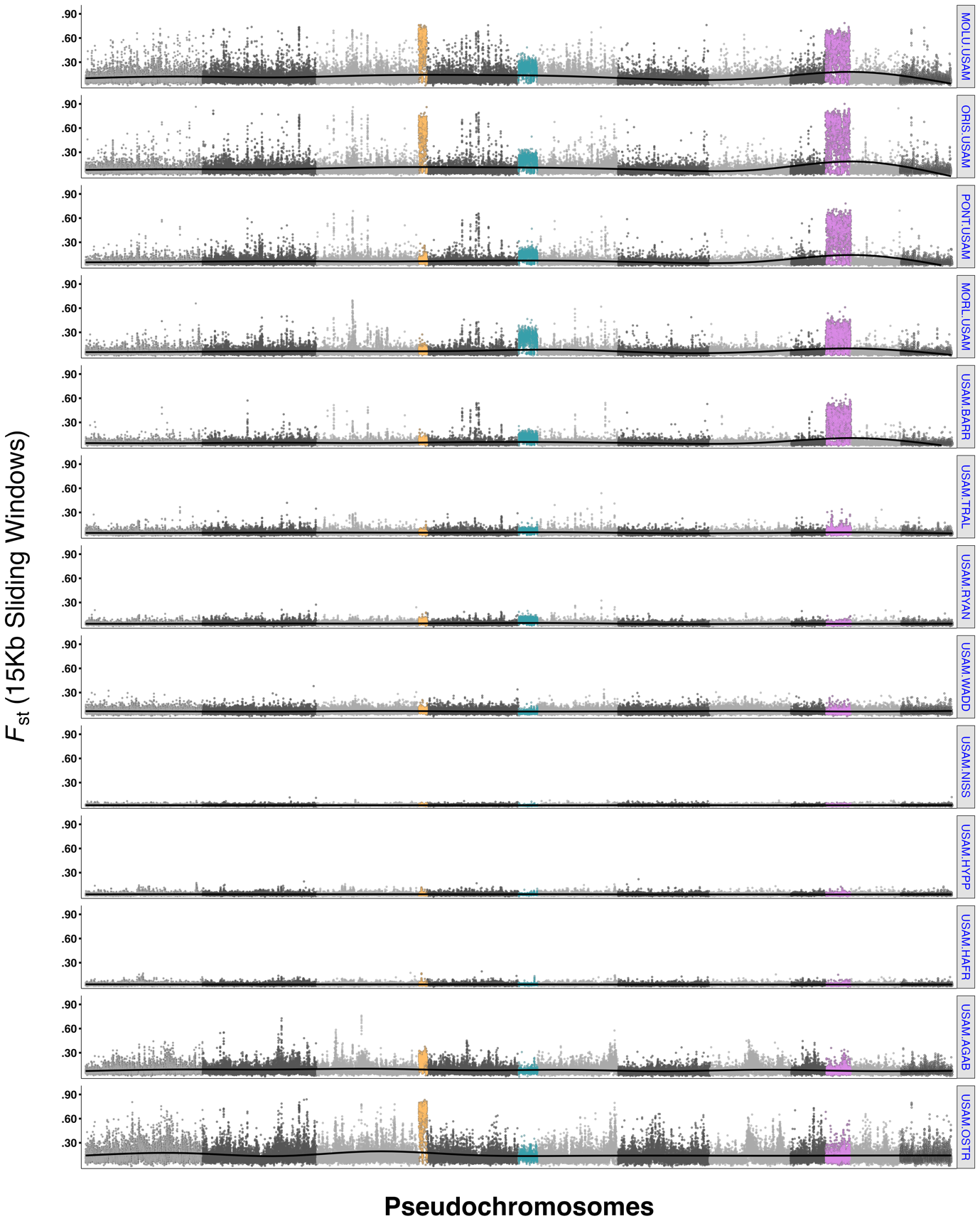


n.
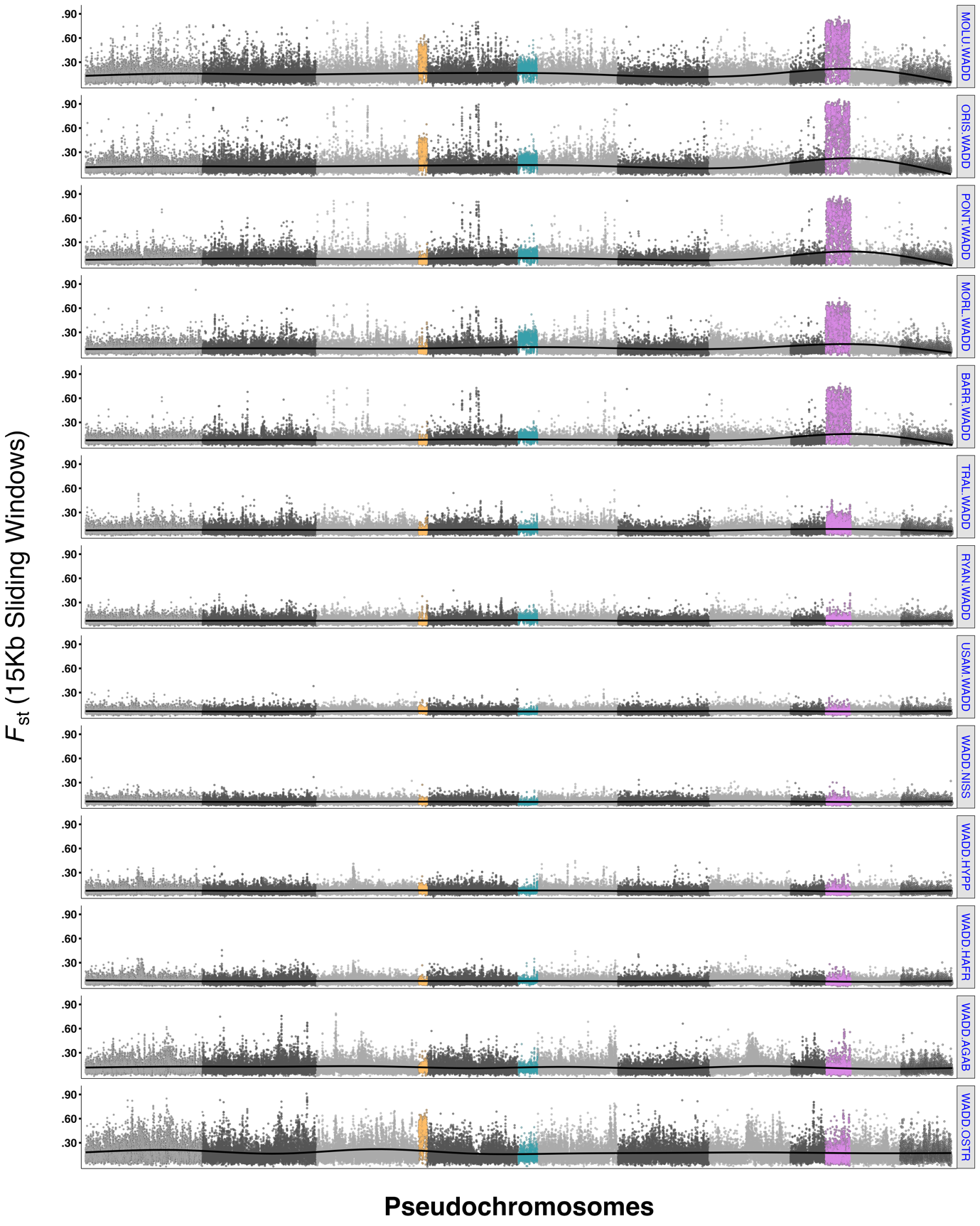


**Fig. S.14 Pairwise *F_ST_* manhattan plots.**

*Rationale*: Manhattan plots displaying pairwise F_ST_ comparisons, estimated using 15 kb sliding-windows across 13 pairs of sampling sites. Within each pairwise comparison (annotated on the y-axis strip box), pseudo-chromosomes 1, 2, 3, 4, 5, 6, 7, 8, 9, 10 are displayed from left to right with different colours (odd number pseudo-chromosomes: grey, even number pseudo-chromosomes: black). Structural variant positions are highlighted in colours (“Chr04:22Kb_sv”: orange, “Chr05:172Kb_sv”: blue, “Chr08:33800Kb_sv”=violet”).

1. Aga Bømlo, (“AGAB”), Norway.
2. Barrow Deep, (“BARR”), UK.
3. Hafrsjford, (“HAFR”), Norway.
4. Hyppeln Gothenburg Archipelago (“HYPP”), Sweden.
5. Molunat, (“MOLU”), Croatia.
6. Morlaix, (“MORL”), France.
7. Nissum, (“NISS”), Denmark.
8. Golfo di Oristano, (“ORIS”), Italy (Sardinia).
9. Ostretjønn Lake, (“OSTR”), Norway.
10. Pontedeume, (“PONT”), Spain.
11. Loch Ryan, (“RYAN”), UK (Scotland).
12. Tralee Bay, (“TRAL”), Ireland.
13. State of Maine, (“USAM”), U.S.A.
14. Wadden Sea, (“WADD”), The Netherlands.


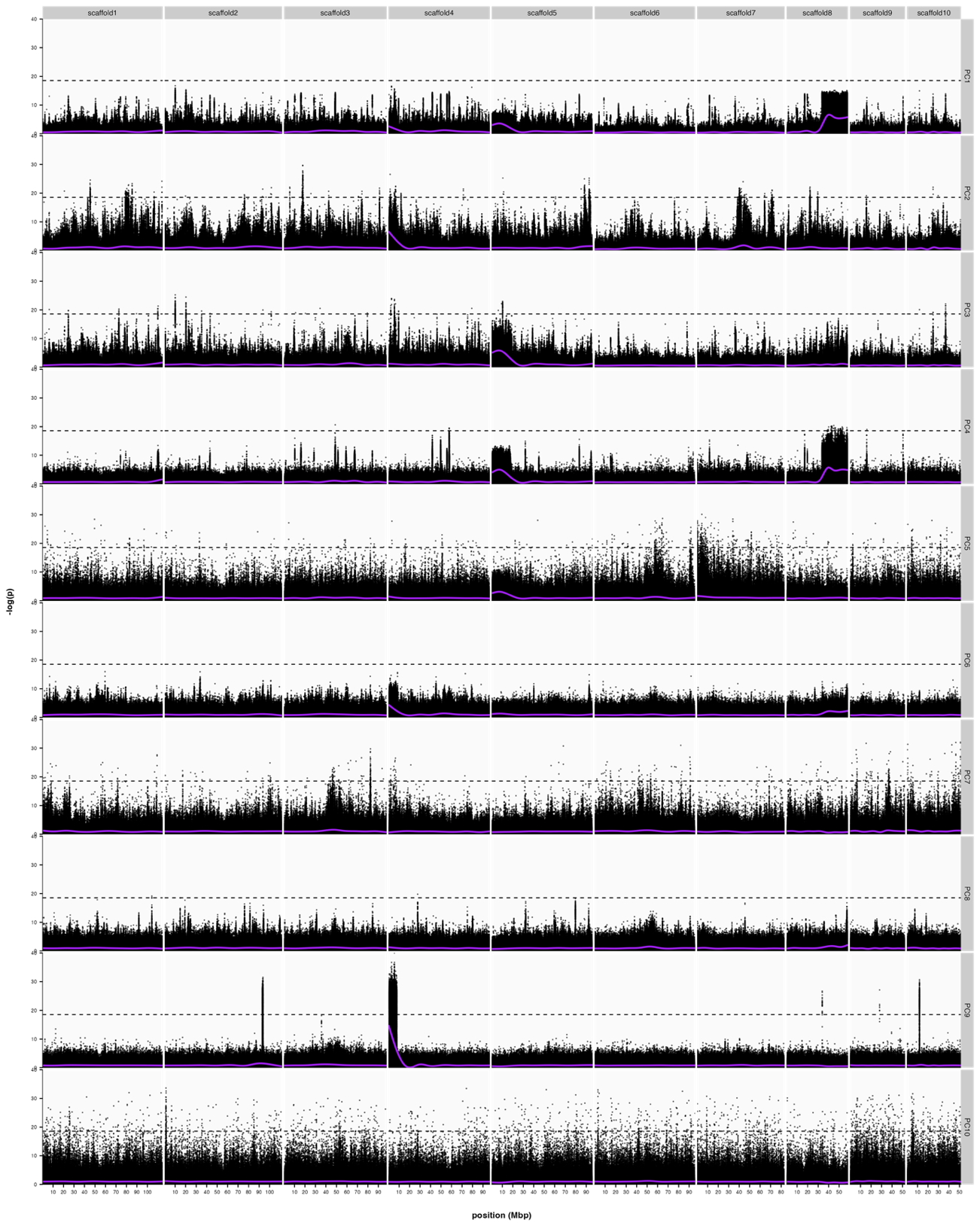


**Fig. S15. PCAngsd selection scan**

Genome-wide selection scan on Dataset I (Global Variant Calling SNPs) using principal component analysis across ten principal components (PC1 to PC10). Each point on the plots represents a SNP, where the x-axis indicates the position in megabase pairs (Mbp), and the y-axis shows the negative logarithm of the *p-value* (-log(p)). A higher -log(p) value suggests stronger evidence against the null hypothesis of no selection. Dashed purple horizontal line across each panel marks the Bonferroni-adjusted significance threshold corresponding to a *p-value* of 0.05.

a.


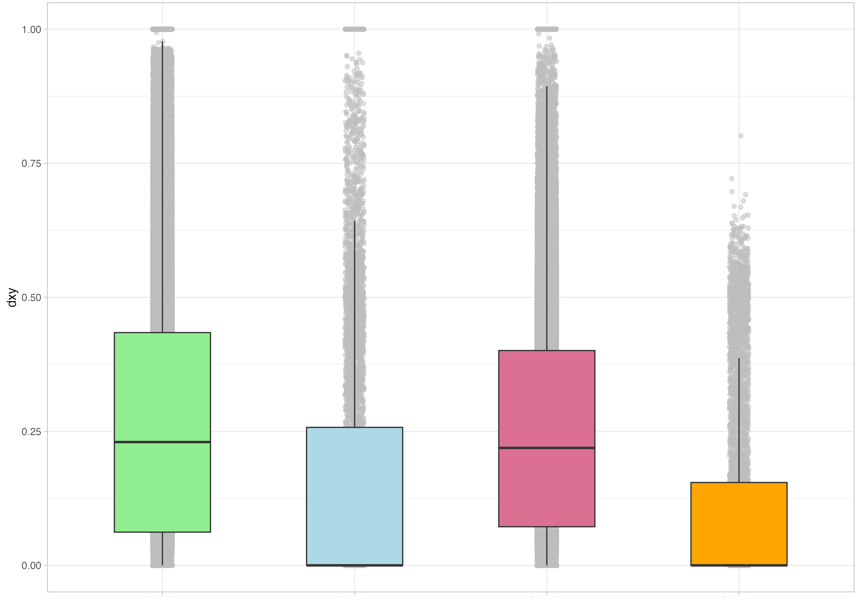


b.


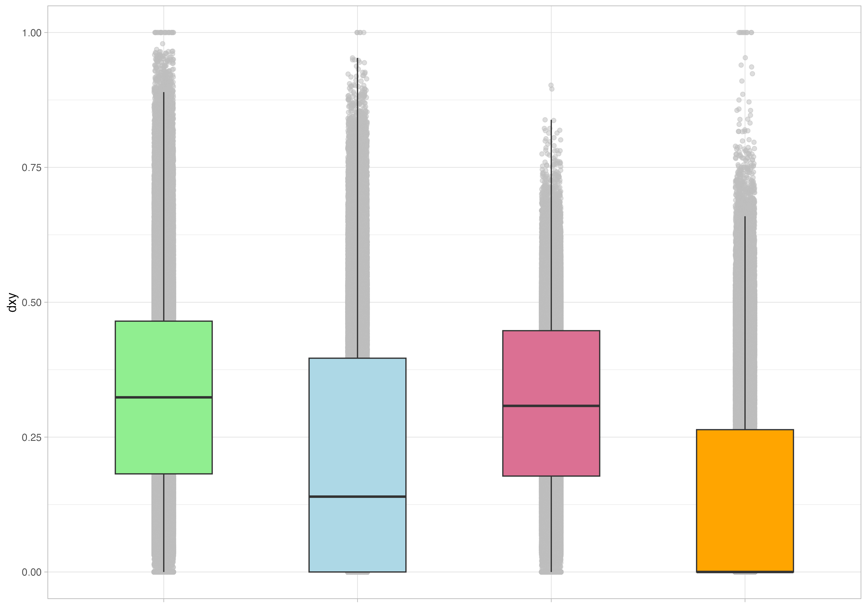


c.


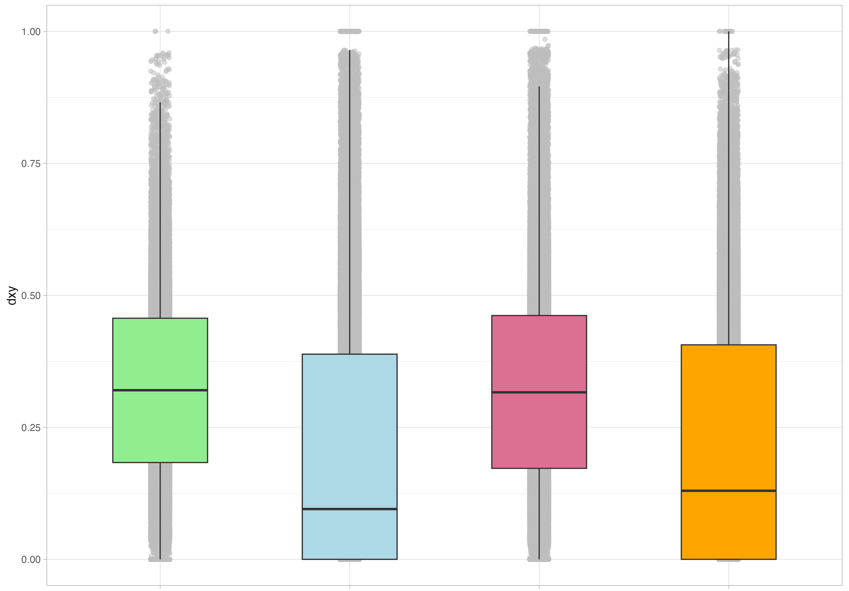


**Fig. S.16 d_xy_ inside and outside SVs**

*Rationale*: Using the PCA for each SV, we identified the top 20 and bottom 20 individuals for pairwise comparison of per-SNP **d_xy_** for the "α" and "β" homozygote genotypes, following PC2 (see illustration Fig S. 17.a). We aimed to measure whether absolute divergence (**d_xy_**) is reduced in the SV region compared to the rest of the pseudo-chromosome. We were also interested in comparing the mean **d_xy_** between “α” and “β” homozygotes group of comparisons.

For each figure, mean **d_xy_** is depicted for four sets of comparisons with a corresponding boxplot:

- Βlue box, pairwise per SNPs **d_xy_** comparisons for the top and bottom 20 individuals with the “α” homozygote genotype, within the SV genomic region.
- Οrange box, pairwise per SNPs **d_xy_** comparisons for the top and bottom 20 individuals with the “β” homozygote genotype, within the SV genomic region.
- Green box, pairwise per SNPs **d_xy_** comparisons for the top and bottom 20 individuals with the “α” homozygote genotype, outside the SV genomic region (excluding SNPs within the SV), but on the same pseudo-chromosome.
- Red box, pairwise per SNPs **d_xy_** comparisons for the top and bottom 20 individuals with the “β” homozygote genotype, outside the SV genomic region (excluding SNPs within the SV), but on the same pseudo-chromosome.

a. “Chr04:22Kb_sv” SV and pseudo-chromosome 4

b. “Chr05:172Kb_sv” SV and pseudo-chromosome 5

c. “Chr08:33800Kb_sv” SV and pseudo-chromosome 8

a.

b.

c.

d.

e.

| **Group** | **Type** | **Region** | **Genotype** | **Mean heterozygosity** |
| --- | --- | --- | --- | --- |
| **HighestPC2HomoAncReg08** | Non-SV | Chr08 |  | 0.019 |
| **HighestPC2HomoAncReg08** | SV | Reg08 | α | 0.018 |
| **HighestPC2HomoDerReg08** | Non-SV | Chr08 |  | 0.021 |
| **HighestPC2HomoDerReg08** | SV | Reg08 | β | 0.018 |
| **LowestPC1HomoDerReg08** | SV | Reg8 | β | 0.016 |
| **LowestPC2HomoAncReg08** | Non-SV | Chr08 |  | 0.022 |
| **LowestPC2HomoAncReg08** | SV | Reg08 | α | 0.021 |
| **LowestPC2HomoDerReg08** | Non-SV | Chr08 |  | 0.018 |
| **HighestPC2HomoAnc05** | SV | Reg05 | α | 0.02 |
| **HighestPC2HomoAnc05** | non-SV | Chr5 |  | 0.021 |
| **HighestPC2HomoDer05** | SV | Reg05 | β | 0.021 |
| **HighestPC2HomoDer05** | non-SV | Chr5 |  | 0.017 |
| **LowestPC2HomoAnc05** | SV | Reg05 | α | 0.016 |
| **LowestPC2HomoAnc05** | non-SV | Chr5 |  | 0.016 |
| **LowestPC2HomoDer05** | SV | Reg05 | β | 0.021 |
| **LowestPC2HomoDer05** | non-SV | Chr5 |  | 0.017 |
| **HighestPC2HomoAnc04** | SV | Reg04 | α | 0.030 |
| **HighestPC2HomoAnc04** | non-SV | Chr4 |  | 0.023 |
| **HighestPC2HomoDer04** | SV | Reg04 | β | 0.011 |
| **HighestPC2HomoDer04** | non-SV | Chr4 |  | 0.017 |
| **LowestPC2HomoAnc04** | SV | Reg04 | α | 0.018 |
| **LowestPC2HomoAnc04** | non-SV | Chr4 |  | 0.017 |
| **LowestPC2HomoDer04** | SV | Reg04 | β | 0.013 |
| **LowestPC2HomoDer04** | non-SV | Chr4 |  | 0.020 |

**Fig. S.17 Average heterozygosity inside and outside SVs, taking both polymorphic and invariable genomic positions into account.**

a. Illustration of procedure used to define groups of individuals within PCAs for the SV regions. Groups of individuals are shown for SV “Chr08:33800Kb_sv” PCA.

b. Individual heterozygosity estimated from each individual, outside (non-SV) and inside SV (SV) for SV “Chr04:22Kb_sv”.

c. Individual heterozygosity estimated from each individual, outside (non-SV) and inside SV (SV) for SV “Chr05:172Kb_sv”.

d. Individual heterozygosity estimated from each individual, outside (non-SV) and inside SV (SV) for SV “Chr08:33800Kb_sv”.

e. Average heterozygosity estimate per group of individuals in b-d.
