# Supplementary material for: Genome‐Wide Population Structure in a Marine Keystone Species, the European Flat Oyster (Ostrea edulis)
Source: Mol Ecol. 2024 Nov 12;34(23):e17573. doi: 10.1111/mec.17573 (PMC12684353; doi:10.1111/mec.17573)
Supplement: Supplementary file 2 — Data S1. [file MEC-34-e17573-s001.docx]

**Supplementary Materials and Methods**

Genome-wide Population Structure in a Marine Keystone Species, the European Flat oyster (*Ostrea edulis*).

Authors: Homère J. Alves Monteiro^1,2*^, Dorte Bekkevold^1^, George Pacheco^1,3^, Stein Mortensen^4^, R. Nicolas Lou^5,6^, Nina O. Therkildsen^5^, Arnaud Tanguy^7^, Chloé Robert^8^, Pierre De Wit^8, 9^, Dorte Meldrup^1^, Ane T. Laugen^10, 11^_,_ Philine S.E. zu Ermgassen^12^, Åsa Strand^13^_,_ Camille Saurel^14^, Jakob Hemmer-Hansen^1*^.

^1^National Institute of Aquatic Resources, Technical University of Denmark, Silkeborg, Denmark, Vejlsøvej 39, 8600, Silkeborg, Denmark

^2^ Section for Evolutionary Genomics, The Globe Institute, Faculty of Health and Medical Sciences, University of Copenhagen, Copenhagen K, Denmark

^3^Department of Biosciences, Centre for Ecological and Evolutionary Synthesis, University of Oslo, Oslo, Norway

^4^Institute of Marine Research, PO Box 1870 Nordnes, 5817 Bergen, Norway

^5^Department of Natural Resources and the Environment, Cornell University, Ithaca, New York, USA

^6^Department of Integrative Biology, University of California Berkeley, Berkeley, CA

^7^Sorbonne Université, CNRS, UMR 7144, Station Biologique de Roscoff, Roscoff, France

^8^Department of Marine Sciences, Tjärnö Marine Laboratory, University of Gothenburg, Laboratorievägen 10, 452 96 Strömstad, Sweden

^9^Department of Biological and Environmental Sciences, University of Gothenburg, Gothenburg, Sweden.

^10^Department of Ecology, Swedish University of Agricultural Sciences, Uppsala, Sweden

^11^Department of Natural Sciences, Centre for Coastal Research, University of Agder, Kristiansand, Norway

^12^Changing Oceans Group, School of Geosciences, University of Edinburgh, James Hutton Rd, King's Buildings, Edinburgh EH9 3FE, United Kingdom

^13^Department of Environmental Intelligence, IVL Swedish Environmental Research Institute, Kristineberg 566, 451 78 Fiskebäckskil, Sweden

^14^National Institute of Aquatic Resources, Technical University of Denmark, Danish Shellfish Centre, Øroddevej 80, 7900 Nykøbing Mors, Denmark

* Corresponding authors: jhh@aqua.dtu.dk; homerejalvesmonteiro@gmail.com

*2.3. Sequence filtering and alignment*

We used Trimmomatic v0.38 (Bolger *et al.*, 2014) to remove adapters using the ILLUMINACLIP mode allowing two mismatches, with a palindrome clip threshold of 30, a simple clip threshold of ten, a minimum adapter length of four, and keeping both reads after clipping.

We indexed the *O. edulis* chromosome-level genome assembly *Roscoff_O.edulis-V1* (Boutet *et al.*, 2022) using the Burrows-Wheeler Alignment tool (Li & Durbin, 2009).

*2.5. Linkage disequilibrium pruning*

Estimates of correlation between loci were calculated directly from genotype likelihoods using an expectation–maximization (EM) algorithm (Fox *et al.*, 2019). The squared Pearson correlation between expected genotypes, r^2^, was calculated from genotype posterior probabilities (Fox *et al.*, 2019). The *prune_ngsLD.py* script was used with the following parameters: --*max_dist* **100000** --*min_weight* **0.5** to prune the highly linked SNPs.

*2.7. Population genetic summary statistics, relatedness and demographic history*

Information on both variant and invariant sites is needed for the estimation of the summary statistics. Therefore the set of filters used to generate per-population SFS slightly differed from the one used in the SNP calling. The depth filters used for the genotype likelihood estimation and SNP identification steps were replaced by the option -*minInd* with the value of **¼** of the total number of individuals in each given population, which specifies the minimum number of individuals that must have mapped reads for a site to be included in the analysis. We ran ANGSD under -*doSaf* to estimate the site allele frequency likelihood for each population.

To estimate relatedness with NgsRelate (Korneliussen & Moltke, 2015), we generated allele frequencies (*-doMaf* **1**) and genotype likelihood files (*-doGlf* **3**) for each population using ANGSD, applying a new set of filters to estimate sampling site alleles frequencies (*-dosnpstat* **1**, *-hwe_pval* **1e-6**, *-SNP_pval* **1e-6**, and the flag *-minInd* at **⅔** of the number of individuals in the sampling sites).

*2.8. FST-based analysis (with* ***Dataset I*** *as primary input)*

To estimate pairwise F_ST_, we first estimated site allele frequency likelihood for each of the sampling sites (-*doSaf* **1**), setting the minimum number of individuals threshold (-*minInd*) to **¼** of the number of individuals from the sampling site. We then estimated the 2D site frequency spectrum for each pair of sampling sites (*realSFS*) and calculated the average pairwise weighted F_ST_ (*realSFS fst*).

*2.9. Genome scans and identification of large structural variants (with* ***Dataset I*** *as primary input)*

For the local PCA analyses, we first applied principal component analyses (PCA) to non-overlapping windows of 1000 SNPs using the Beagle file generated from **Dataset I** using PCAngsd. The Euclidean distance between PCA results at every pair of windows was calculated with *lostruct*. This approach provides a measure of how similar patterns of population structure are across the windows. A multidimensional scaling (MDS) was then applied to the distance matrix, and clusters of outlier windows with a z-score **> 4** along each MDS dimension were identified (see Huang *et al.*, 2020; Mérot *et al.*, 2021). These outlier windows present similar PCA patterns among themselves that are distinct from the genome-wide pattern and thus are potential candidates for structural variants or targets of selection.

*References*
Boutet, I., Alves Monteiro, H. J., Baudry, L., Takeuchi, T., Bonnivard, E., Billoud, B., Farhat, S., Gonzales‐Araya, R., Salaun, B., Andersen, A. C., Toullec, J., Lallier, F. H., Flot, J., Guiglielmoni, N., Guo, X., Li, C., Allam, B., Pales‐Espinosa, E., Hemmer‐Hansen, J., Moreau, P., Marbouty, M., Koszul. R., Tanguy, A. (2022). Chromosomal assembly of the flat oyster (*Ostrea edulis L*.) genome as a new genetic resource for aquaculture. *Evolutionary Applications, 15(11)*, 1730–1748. https://doi.org/10.1111/eva.13462

Fox, E. A., Wright, A. E., Fumagalli, M., & Vieira, F. G. (2019). ngsLD: evaluating linkage disequilibrium using genotype likelihoods. *Bioinformatics, 35(19)*, 3855–3856. https://doi.org/10.1093/bioinformatics/btz200

Huang, K., Andrew, R. L., Owens, G. L., Ostevik, K. L., & Rieseberg, L. H. (2020). Multiple chromosomal inversions contribute to adaptive divergence of a dune sunflower ecotype. *Molecular Ecology, 29(14)*, 2535–2549. https://doi.org/10.1111/mec.15428

Korneliussen, T. S., & Moltke, I. (2015). NgsRelate: A software tool for estimating pairwise relatedness from next-generation sequencing data. *Bioinformatics, 31(24)*, 4009–4011. https://doi.org/10.1093/bioinformatics/btv509

Li, H., & Durbin, R. (2009). Fast and accurate short read alignment with Burrows–Wheeler transform. *Bioinformatics, 25(14)*, 1754–1760. https://doi.org/10.1093/bioinformatics/btp324

Mérot, C., Berdan, E. L., Cayuela, H., Djambazian, H., Ferchaud, A.-L., Laporte, M., Normandeau, E., Ragoussis, J., Wellenreuther, M., & Bernatchez, L. (2021). Locally Adaptive Inversions Modulate Genetic Variation at Different Geographic Scales in a Seaweed Fly. *Molecular Biology and Evolution, 38(9*), 3953–3971. https://doi.org/10.1093/molbev/msab143
