# Supplementary material for: Genome‐Wide Population Structure in a Marine Keystone Species, the European Flat Oyster (Ostrea edulis)
Source: Mol Ecol. 2024 Nov 12;34(23):e17573. doi: 10.1111/mec.17573 (PMC12684353; doi:10.1111/mec.17573)
Supplement: Supplementary file 3 — Tables S1. Tables S2. Tables S3. [file MEC-34-e17573-s003.docx]

**Supplementary tables**

Genome-wide Population Structure in a Marine Keystone Species, the European Flat oyster (*Ostrea edulis*).

Authors: Homère J. Alves Monteiro^1,2*^, Dorte Bekkevold^1^, George Pacheco^1,3^, Stein Mortensen^4^, R. Nicolas Lou^5,6^, Nina O. Therkildsen^5^, Arnaud Tanguy^7^, Chloé Robert^8^, Pierre De Wit^8, 9^, Dorte Meldrup^1^, Ane T. Laugen^10, 11^_,_ Philine S.E. zu Ermgassen^12^, Åsa Strand^13^_,_ Camille Saurel^14^, Jakob Hemmer-Hansen^1*^.

^1^National Institute of Aquatic Resources, Technical University of Denmark, Silkeborg, Denmark, Vejlsøvej 39, 8600, Silkeborg, Denmark

^2^ Section for Evolutionary Genomics, The Globe Institute, Faculty of Health and Medical Sciences, University of Copenhagen, Copenhagen K, Denmark

^3^Department of Biosciences, Centre for Ecological and Evolutionary Synthesis, University of Oslo, Oslo, Norway

^4^Institute of Marine Research, PO Box 1870 Nordnes, 5817 Bergen, Norway

^5^Department of Natural Resources and the Environment, Cornell University, Ithaca, New York, USA

^6^Department of Integrative Biology, University of California Berkeley, Berkeley, CA

^7^Sorbonne Université, CNRS, UMR 7144, Station Biologique de Roscoff, Roscoff, France

^8^Department of Marine Sciences, Tjärnö Marine Laboratory, University of Gothenburg, Laboratorievägen 10, 452 96 Strömstad, Sweden

^9^Department of Biological and Environmental Sciences, University of Gothenburg, Gothenburg, Sweden.

^10^Department of Ecology, Swedish University of Agricultural Sciences, Uppsala, Sweden

^11^Department of Natural Sciences, Centre for Coastal Research, University of Agder, Kristiansand, Norway

^12^Changing Oceans Group, School of Geosciences, University of Edinburgh, James Hutton Rd, King's Buildings, Edinburgh EH9 3FE, United Kingdom

^13^Department of Environmental Intelligence, IVL Swedish Environmental Research Institute, Kristineberg 566, 451 78 Fiskebäckskil, Sweden

^14^National Institute of Aquatic Resources, Technical University of Denmark, Danish Shellfish Centre, Øroddevej 80, 7900 Nykøbing Mors, Denmark

* Corresponding authors: jhh@aqua.dtu.dk; homerejalvesmonteiro@gmail.com

**Supplementary Table S1. LcWGS Datasets***.*

LcWGS datasets used in the study, each with a specific analytical purpose. The table includes the number of individuals (N) in each dataset, the total number of sites or single nucleotide polymorphisms (SNPs) analyzed, and the software used to generate or process the dataset. Additionally, the reasoning behind the use of each dataset is explained, including the specific analyses conducted and their objectives.

| Dataset | No. individuals | No. sampling locations | No. of SNPs | Generated by (software) | Reasoning |
| --- | --- | --- | --- | --- | --- |
| Dataset I  (*Global Variant Calling SNPs)* | 582 | 33 | 5,684,643 SNPs | ANGSD | Investigate linkage disequilibrium patterns, average pairwise weighted *F_ST_*, and pairwise *F_ST_* in non overlapping sliding windows |
| Dataset II  *(LD pruned SNPs)* | 582 | 33 | 1,404,180 SNPs | ngsLD | Result of Linkage Disequilibrium (LD) estimation and pruning on Dataset I; used to identify population structure (PCA) and natural admixture (Admixture plot) |
| Dataset III *(SFS for each population)* | 582 | 33 | Varies among populations.  Mean= 434,311,511 sites | ANGSD | Obtain population genetic estimates using the Site Frequency Spectrum (SFS) analysis for each population |
| Dataset IV *(Allele frequency and Site Frequency Spectrum for all the individuals at all sites)* | 582 | 33 | 484,110,305 | ANGSD | Obtain per-individual heterozygosity estimation using as inputs the allele frequencies inferred on the genotypes likelihoods. |
| Dataset V *(Allele frequencies per individual for each sampling location for relatedness calculation)* | 582 | 33 | 1,404,180 SNPs | ANGSD | Obtain per-sampling location relatedness calculation using as inputs the allele frequencies inferred on genotypes likelihoods |
| Dataset VI *(F_ST_ for each pair of sampling locations)* | 582 | 33 | Depends on the population | ANGSD | Calculate *F_ST_* averaged over Dataset I (Global Variant Calling SNPs) with global minimum allele frequency >= 0.05 |
| Dataset VII Higher Coverage | 7 | 6 | - | - | Historical demography analysis |

**Supplementary Table S2. Genomic locations of the 202 SNPs analyzed by Lapègue *et al.* (2022).**

Genomic positions in the reference genome used in the current study of genetic markers analyzed in Lapègue *et al.* (2022). Markers in bold were located in linkage blocks identified in Lapègue *et al.* (2022), and have a significant match in the reference genome.

| Locus | Pseudo-chromosome (Boutet *et al.*, 2022 | Position in the pseudo-chromosome | Lapègue *et al.* 2022 (LDG) |
| --- | --- | --- | --- |
| SNP_1 | scaffold4 | 80770475 |  |
| SNP_2 | No_hits |  |  |
| SNP_3 | Multiple_hits |  |  |
| SNP_4 | scaffold8 | 10311338 |  |
| **SNP_5** | **scaffold5** | **6844113** | **LDG_202** |
| **SNP_6** | **scaffold5** | **13870815** | **LDG_202** |
| SNP_7 | scaffold5 | 90641000 |  |
| **SNP_8** | **scaffold8** | **56574939** | **LDG_182** |
| SNP_9 | scaffold5 | 69820293 |  |
| SNP_10 | scaffold10 | 9393483 |  |
| SNP_11 | No_hits |  | LDG_182 |
| SNP_12 | scaffold2 | 23661650 |  |
| SNP_13 | scaffold2 | 20392259 |  |
| SNP_14 | scaffold8 | 13736531 |  |
| SNP_15 | scaffold7 | 46753672 |  |
| SNP_16 | scaffold4 | 22367965 |  |
| SNP_17 | scaffold3 | 94077032 |  |
| SNP_18 | scaffold5 | 57652822 |  |
| SNP_19 | No_hits |  |  |
| SNP_20 | scaffold2 | 90176641 |  |
| SNP_21 | scaffold2 | 67920131 |  |
| SNP_22 | scaffold1 | 29504892 |  |
| SNP_23 | scaffold2 | 22852495 |  |
| SNP_24 | scaffold4 | 4359864 |  |
| SNP_25 | scaffold4 | 28625497 |  |
| SNP_26 | scaffold5 | 54987535 |  |
| SNP_27 | scaffold5 | 12418483 |  |
| **SNP_28** | **scaffold8** | **41727763** | **LDG_182** |
| SNP_29 | scaffold1 | 68768148 |  |
| SNP_30 | scaffold8 | 33205774 |  |
| SNP_31 | scaffold6 | 53108708 |  |
| SNP_32 | scaffold5 | 35973476 |  |
| SNP_33 | scaffold4 | 79319874 |  |
| SNP_34 | scaffold3 | 84121536 |  |
| SNP_35 | scaffold2 | 89214474 |  |
| SNP_36 | scaffold7 | 17946999 |  |
| SNP_37 | No_hits |  |  |
| **SNP_38** | **scaffold8** | **48262304** | **LDG_182** |
| SNP_39 | Multiple_hits |  | LDG_182 |
| SNP_40 | scaffold5 | 54857331 |  |
| SNP_41 | scaffold1 | 50092758 |  |
| SNP_42 | scaffold8 | 8668605 |  |
| SNP_43 | scaffold7 | 57043734 |  |
| SNP_44 | scaffold7 | 48098238 |  |
| SNP_45 | scaffold3 | 5920916 |  |
| SNP_46 | scaffold3 | 61789230 |  |
| SNP_47 | scaffold5 | 44008184 |  |
| SNP_48 | scaffold2 | 60431656 |  |
| SNP_49 | scaffold10 | 39946179 |  |
| SNP_50 | scaffold4 | 6053161 |  |
| SNP_51 | Multiple_hits |  |  |
| SNP_52 | scaffold6 | 69662103 |  |
| SNP_53 | scaffold3 | 70813410 |  |
| **SNP_54** | **scaffold8** | **37028110** | **LDG_182** |
| SNP_55 | scaffold4 | 92571821 |  |
| SNP_56 | scaffold3 | 4457380 |  |
| **SNP_57** | **scaffold8** | **52512017** | **LDG_182** |
| SNP_58 | scaffold3 | 68861674 |  |
| SNP_59 | scaffold3 | 21898485 |  |
| SNP_60 | scaffold4 | 9912731 |  |
| SNP_61 | scaffold4 | 61781906 |  |
| SNP_62 | scaffold7 | 26385229 |  |
| SNP_63 | No_hits |  |  |
| SNP_64 | scaffold3 | 11248239 |  |
| **SNP_65** | **scaffold5** | **16259987** | **LDG_202** |
| SNP_66 | scaffold3 | 27638611 |  |
| SNP_67 | No_hits |  |  |
| SNP_68 | No_hits |  | LDG_182 |
| SNP_69 | scaffold2 | 100682819 |  |
| SNP_70 | No_hits |  |  |
| SNP_71 | scaffold6 | 83172735 |  |
| SNP_72 | scaffold1 | 84710810 |  |
| SNP_73 | scaffold8 | 43416493 |  |
| SNP_74 | scaffold1 | 63776043 |  |
| SNP_75 | scaffold2 | 65843593 |  |
| SNP_76 | scaffold1 | 44440916 |  |
| SNP_77 | scaffold6 | 54543439 |  |
| SNP_78 | scaffold3 | 66272226 |  |
| SNP_79 | scaffold4 | 53816433 |  |
| SNP_80 | Multiple_hits |  |  |
| SNP_81 | scaffold4 | 66578264 |  |
| SNP_82 | scaffold1 | 43709496 |  |
| SNP_83 | scaffold5 | 20078368 |  |
| SNP_84 | scaffold2 | 28789819 |  |
| SNP_85 | scaffold10 | 14309835 |  |
| SNP_86 | scaffold3 | 16661540 |  |
| SNP_87 | scaffold5 | 79354960 |  |
| SNP_88 | scaffold5 | 28343518 |  |
| SNP_89 | Multiple_hits |  | LDG_182 |
| SNP_90 | scaffold1 | 58812845 |  |
| SNP_91 | scaffold1 | 70103933 |  |
| SNP_92 | scaffold4 | 60931604 |  |
| SNP_93 | Multiple_hits |  |  |
| SNP_94 | No_hits |  |  |
| SNP_95 | No_hits |  |  |
| SNP_96 | scaffold5 | 41423781 |  |
| SNP_97 | scaffold2 | 16933191 |  |
| SNP_98 | scaffold5 | 19532180 |  |
| SNP_99 | scaffold1 | 106504540 |  |
| SNP_100 | scaffold4 | 21545843 |  |
| SNP_101 | scaffold1 | 107618638 |  |
| SNP_102 | scaffold5 | 86230654 |  |
| SNP_103 | scaffold5 | 12990526 |  |
| SNP_104 | scaffold1 | 981125 |  |
| SNP_105 | scaffold1 | 85846677 |  |
| SNP_106 | Multiple_hits |  |  |
| SNP_107 | scaffold1 | 62522281 |  |
| SNP_108 | scaffold5 | 19501462 |  |
| SNP_109 | scaffold4 | 15393294 |  |
| SNP_110 | scaffold2 | 90844847 |  |
| SNP_111 | scaffold3 | 64888552 |  |
| SNP_112 | scaffold6 | 35555238 |  |
| SNP_113 | scaffold1 | 99397160 |  |
| **SNP_114** | **scaffold5** | **13340931** | **LDG_202** |
| SNP_115 | scaffold5 | 20116694 |  |
| **SNP_116** | **scaffold5** | **3662530** | **LDG_202** |
| SNP_117 | scaffold4 | 44885254 |  |
| SNP_118 | scaffold2 | 53454775 |  |
| SNP_119 | scaffold6 | 27483417 |  |
| SNP_120 | scaffold1 | 32563531 |  |
| SNP_121 | scaffold4 | 85281891 |  |
| SNP_122 | Multiple_hits |  |  |
| SNP_123 | scaffold7 | 32314992 |  |
| SNP_124 | scaffold5 | 3901756 |  |
| SNP_125 | scaffold5 | 12410936 |  |
| SNP_126 | scaffold8 | 38307220 |  |
| SNP_127 | scaffold2 | 74628252 |  |
| SNP_128 | scaffold3 | 33714860 |  |
| SNP_129 | scaffold4 | 53330190 |  |
| SNP_130 | scaffold3 | 90953310 |  |
| SNP_131 | scaffold10 | 12681801 |  |
| **SNP_132** | **scaffold8** | **56906476** | **LDG_182** |
| SNP_133 | scaffold1 | 106611832 |  |
| SNP_134 | Multiple_hits |  |  |
| SNP_135 | scaffold4 | 66340142 |  |
| SNP_136 | scaffold2 | 47779283 |  |
| SNP_137 | scaffold2 | 22106484 |  |
| SNP_138 | scaffold3 | 50724811 |  |
| SNP_139 | scaffold1 | 53238616 |  |
| SNP_140 | scaffold4 | 33092607 |  |
| SNP_141 | scaffold6 | 25966363 |  |
| SNP_142 | scaffold5 | 80099726 |  |
| SNP_143 | scaffold10 | 41121881 |  |
| SNP_144 | scaffold3 | 59728798 |  |
| SNP_145 | No_hits |  | LDG_182 |
| SNP_146 | scaffold3 | 90215049 |  |
| SNP_147 | scaffold1 | 46744790 |  |
| SNP_148 | scaffold5 | 21579753 |  |
| SNP_149 | scaffold2 | 48270288 |  |
| SNP_150 | scaffold1 | 77563251 |  |
| SNP_151 | scaffold1 | 71411088 |  |
| SNP_152 | scaffold2 | 36418916 |  |
| SNP_153 | scaffold7 | 10319728 |  |
| SNP_154 | scaffold2 | 39750269 |  |
| SNP_155 | Multiple_hits |  | LDG_182 |
| **SNP_156** | **scaffold8** | **40463172** | **LDG_182** |
| SNP_157 | scaffold5 | 10107737 |  |
| SNP_158 | scaffold1 | 66925154 |  |
| SNP_159 | scaffold4 | 90641557 |  |
| SNP_160 | scaffold4 | 29977135 |  |
| SNP_161 | scaffold8 | 30708122 |  |
| SNP_162 | scaffold5 | 71378843 |  |
| SNP_163 | scaffold5 | 43208142 |  |
| **SNP_164** | **scaffold5** | **13350379** | **LDG_202** |
| SNP_165 | scaffold4 | 31746443 |  |
| SNP_166 | Multiple_hits |  |  |
| **SNP_167** | **scaffold5** | **73601822** | **LDG_202** |
| SNP_168 | scaffold3 | 5372665 |  |
| SNP_169 | scaffold3 | 68866188 |  |
| SNP_170 | scaffold7 | 58439252 |  |
| SNP_171 | scaffold4 | 24440551 |  |
| SNP_172 | scaffold9 | 26642496 |  |
| SNP_173 | scaffold6 | 6065199 |  |
| SNP_174 | scaffold2 | 43464371 |  |
| SNP_175 | scaffold8 | 5575465 |  |
| SNP_176 | scaffold10 | 18274788 |  |
| SNP_177 | scaffold3 | 77467879 |  |
| SNP_178 | scaffold3 | 53476073 |  |
| SNP_179 | scaffold5 | 52398003 |  |
| SNP_180 | scaffold1 | 50037338 |  |
| SNP_181 | scaffold4 | 24189702 |  |
| **SNP_182** | **scaffold8** | **37868843** | **LDG_182** |
| SNP_183 | scaffold4 | 83586235 |  |
| SNP_184 | scaffold3 | 72132387 |  |
| SNP_185 | scaffold7 | 18564372 |  |
| **SNP_186** | **scaffold5** | **17011493** | **LDG_202** |
| SNP_187 | scaffold5 | 49621339 |  |
| SNP_188 | scaffold2 | 89597844 |  |
| SNP_189 | scaffold9 | 4022587 |  |
| SNP_190 | scaffold6 | 40881768 |  |
| SNP_191 | scaffold4 | 17985987 |  |
| SNP_192 | scaffold2 | 93560385 |  |
| SNP_193 | scaffold10 | 35013454 |  |
| SNP_194 | scaffold6 | 83064362 |  |
| SNP_195 | scaffold1 | 75067426 |  |
| SNP_196 | scaffold7 | 58289764 |  |
| SNP_197 | scaffold1 | 74225591 |  |
| SNP_198 | scaffold10 | 30600092 |  |
| SNP_199 | scaffold3 | 91349897 |  |
| SNP_200 | scaffold5 | 43556626 |  |
| SNP_201 | scaffold5 | 68160159 |  |
| **SNP_202** | **scaffold5** | **11038087** | **LDG_202** |
| SNP_203 | scaffold2 | 63477059 |  |

**Supplementary Table. S.3: HWE departure test for SV genotypes**

*P-values* for exact tests for departures from HWE expectations per sampling site and SV.

| Sampling site | Reg04 (P-value) | Reg05 (P-value) | Reg08 (P-value) |
| --- | --- | --- | --- |
| AGAB | 0.2515 | 1.0000 | - |
| BARR | 0.1312 | 1.0000 | 0.5122 |
| BUNN | - | 0.5392 | - |
| CLEW | 0.4393 | 1.0000 | 1.0000 |
| COLN | 0.1259 | 1.0000 | 1.0000 |
| CORS | - | 1.0000 | 0.4767 |
| CRES | - | 1.0000 | 1.0000 |
| DOLV | 1.0000 | 1.0000 | - |
| GREV | 0.5904 | 1.0000 | - |
| HAFR | 0.2791 | 0.6193 | - |
| HALS | 0.3263 | 1.0000 | - |
| HAUG | 0.1448 | 0.1376 | - |
| HYPP | 1.0000 | 1.0000 | - |
| INNE | 1.0000 | 0.6068 | - |
| KALV | 0.5390 | 0.2624 | - |
| LANG | 1.0000 | 1.0000 | 1.0000 |
| LOGS | 0.2638 | 0.6064 | 1.0000 |
| MOLU | - | 1.0000 | 1.0000 |
| MORL | 0.1619 | - | 0.2600 |
| NISS | 1.0000 | 0.3796 | 1.0000 |
| ORIS | - | - | - |
| OSTR | - | 0.1080 | - |
| PONT | 0.0388 | 1.0000 | 1.0000 |
| RIAE | 0.3531 | 1.0000 | 0.4897 |
| RYAN | 0.6689 | 1.0000 | 1.0000 |
| THIS | 0.2545 | 1.0000 | - |
| TOLL | 1.0000 | 0.5117 | 0.6081 |
| TRAL | 0.3204 | 0.1595 | 1.0000 |
| USAM | 0.4888 | 0.6541 | 1.0000 |
| VAGS | 1.0000 | 0.3679 | 1.0000 |
| VENO | 1.0000 | 0.2004 | - |
| WADD | 0.1620 | 0.0013 | - |
| ZECE | - | 1.0000 | 1.0000 |
